# Effectiveness and impact of the 2-component acellular pertussis vaccine as a preschool booster in Finland – A register-based study

**DOI:** 10.1371/journal.pgph.0006800

**Published:** 2026-07-29

**Authors:** Jussi A. Halme, Ritva K. Syrjänen, Hanna Rinta-Kokko, Kimmo Niinimäki, Anu Soininen, Sorin Abrudan, Juan Camilo Vargas-Zambrano, Arto A. Palmu

**Affiliations:** 1 FVR-Finnish Vaccine Research, RWE-Unit, Tampere, Finland; 2 Sanofi Finland, Espoo, Finland; 3 Sanofi Global Vaccines Medical Affairs, Lyon, France; University of Alabama at Birmingham, UNITED STATES OF AMERICA

## Abstract

In 2005, Finland replaced the whole-cell pertussis (wP) 3 + 1 schedule with an acellular pertussis (aP) 2 + 1 infant schedule supplemented by a DTaP-IPV preschool booster at age 4 in the National Immunization Program (NIP). Recent pertussis resurgence and uncertainty about the duration of vaccine-induced protection have raised concerns. We assessed the effectiveness, duration of protection and population-level impact of the preschool booster. We conducted a nationwide population-based register study in Finland (1995–2019). Vaccine effectiveness (VE) of a two-component (2aP) preschool booster against laboratory-confirmed pertussis was estimated by comparing children with and without the booster, followed from age of 4.25 years during 2011–2019 using a covariate-adjusted Cox proportional hazards model. Population-level impact was assessed by comparing pertussis incidence before (1995–2002; wP primary series without preschool booster) and after (2012–2019; aP primary series with the booster) its introduction. The primary cohort included 466 240 children (94% vaccination coverage). Among 225 cases, 88% were diagnosed serologically and 12% by culture or PCR. VE against any laboratory-confirmed pertussis during 5-year follow-up was 51% (95% CI, 27–67), with VE of 86% (95% CI, 67–94) against PCR/culture-confirmed and 37% (95% CI, −1–60) against serology-confirmed cases. VE remained stable up to 8 years post-vaccination without clear waning. Following booster introduction in the NIP, pertussis incidence decreased by 65% (95% CI, 60–69) among children ≥4.25 years and by 39% (95% CI, 30–48) among those ≤3.75 years, indicating both direct and indirect effects. The 2aP preschool booster seems to provide moderate and sustained protection against pertussis compared to children without the booster. Effectiveness was notably higher against PCR/culture-confirmed cases throughout the follow-up, likely due to lower specificity of serology and differences in diagnostic practices by disease manifestations. These findings support continued use of the preschool booster in Finland.

## Introduction

Pertussis, caused by *Bordetella pertussis,* remains a leading cause of vaccine-preventable childhood morbidity and mortality worldwide, despite widespread vaccination [1]. Most high-income countries transitioned from whole-cell (wP) to acellular (aP) pertussis vaccines in the late 1990s and early 2000s, mainly driven by safety and tolerability considerations. Although pertussis vaccines provide good short-term protection against disease [2], *B. pertussis* continues to circulate, and epidemics still occur. During the last decades, even countries with high vaccine coverage have experienced a resurgence of pertussis incidence [3]. Previous studies have suggested that immunity following aP vaccination wanes over time and that aP vaccines have limited ability in preventing transmission [2]. Nevertheless, as more evidence is generated in this area, more recent empirical and modeling studies have challenged this view, suggesting a longer duration of protection [4].

In Finland, a combined diphtheria–tetanus–whole-cell pertussis (DTwP) vaccine produced by the National Public Health Institute (KTL), the predecessor to Finnish Institute for Health and Welfare (THL), was introduced in the National Immunization Program (NIP) in the 1970s.) It was offered free of charge to all children at 3, 4, 5, and 24 months of age in the public well-baby clinic network, and no additional boosters were used. In 2005, acellular pertussis vaccines (aP), and a vaccination schedule of 3, 5, and 12 months was introduced in the NIP. The aP vaccine was a part of a pentavalent combination vaccine with diphtheria, tetanus, inactivated polio, and *Haemophilus influenzae* type B antigens (DTaP-IPV-Hib). After a transitional phase (starting in 2003) with a preschool booster at 6 or 11–13 years of age, the fourth dose was scheduled at 4 years of age (starting in 2008) as a tetravalent combination with diphtheria, tetanus, and inactivated polio vaccines (DTaP-IPV) [5,6], (Table A in S1 File). A low-dose dTap vaccine (fifth dose) was added for adolescents 14–15 years of age in 2005 as well. Subsequent program updates included booster doses for conscripts (2012), young adults aged 25 years, and healthcare workers caring for infants (2018). Vaccinations recommended to pregnant women were not included in the NIP until 2024 [7].

Following the introduction of aP vaccines, pertussis incidence in Finland remained relatively stable until the COVID-19 pandemic, during which case numbers dropped sharply. However, in 2024–2025, Finland experienced its largest pertussis epidemic since the establishment of the National Infectious Disease Register (NIDR) in 1995 [8]. Despite long-term use in Finland, there is no existing research on the direct effectiveness of aP vaccines, although epidemiological data from various Scandinavian countries show that the 2-component aP (2aP) vaccine as a preschool booster provides adequate protection at the population level [9].

Understanding the effectiveness and duration of protection conferred by pertussis vaccines is essential for optimizing vaccination strategies, including timing of booster doses and other measures to protect the most vulnerable to the disease. Hence, the primary objective of this study was to evaluate the effectiveness of a 2aP vaccine administered as a preschool booster at 4 years of age against laboratory-confirmed pertussis during a 5-year follow-up. We also aimed to determine the duration of protection and the population-level impact after implementation in the NIP. All objectives are detailed in S1 File.

## Materials and methods

This was a nationwide population-based register study in Finland. The study population comprised all permanent residents aged under 14 years between 1995 and 2019, as identified from the Finnish Population Information System. We used routinely collected data from several national health and administrative registers linked via a unique personal identity code assigned to each resident. Detailed descriptions of the data sources are provided in S1 File. Data was extracted and then delivered to the study team in 2024 by the Finnish Social and Health Data Permit Authority, Findata

### Cohort study

**Fig 1:**
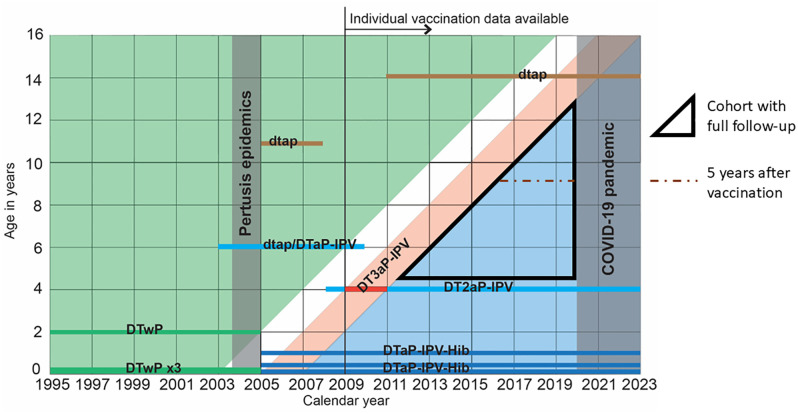
Cohort design for effectiveness of the 2-component acellular pertussis preschool booster dose in 2011–2019. The triangle represents the children followed from the age of 4.25 years to December 31, 2019, in the cohort analysis. For details of the vaccination programs, see Table A in S1 File. Abbreviations: DTwP, combination vaccine containing whole-cell pertussis component; DTaP-IPV (+/- Hib), combination vaccine containing acellular pertussis components; 3aP, 3-component acellular pertussis vaccine; 2aP, 2-component acellular pertussis vaccine; dTap, low-dose combination vaccine containing acellular pertussis components.

**Cohort design** was used to estimate the effectiveness of 2aP preschool pertussis booster vaccine against pertussis outcomes. We followed up children turning 4.25 years old during 2011–2019. (Fig 1)

#### Study setting and population for cohort design.

The population eligible for the cohort analysis consisted of children born from January 2007 through September 2015, recorded by the Digital and population data services agency (DVV) as permanent residents in Finland. Permanent residency in Finland was defined as having no record of living abroad between the age of 3 years and the start age of the follow-up. Each subject had to be alive at the start age of the follow-up to be included in the analysis cohort.

Excluded from all cohort analyses were children who lived in municipalities with inadequate vaccination reporting to the National vaccination register (NVR) for the study years, received any pertussis vaccination other than 2aP between the age 3 years and the start age of the follow-up, had an ambiguous birth date in the data available, or had a record of vaccination dated before the birth date.

For analyses by previous vaccination status, only children born between January 2011 and September 2015 were included to ensure sufficient information on the primary (infant) pertussis vaccination doses, as reliable vaccination data from NVR is only available from 2011 onwards. Additionally excluded were children who a) had missing residency data for a period of 29 days or longer in DVV municipality history data either between two consecutive records or between birth date and the date of the first record; b) received any pertussis vaccination other than primary series dose before the age of 3 years; c) received an additional primary series dose after the third one and before the age of 3 years; or d) had insufficient break between doses (<28 days between the first and the second primary series doses, < 181 days between the second and the third primary series doses or <730 days between the third primary series dose and booster dose).

The primary effectiveness analysis of the cohort study comprised the years 2011–2019, between the first availability of reliable vaccination data and the COVID-19 pandemic. The follow-up period for each child started from the age of 4.25 years and lasted until the first outcome event of interest, any pertussis vaccination other than 2aP, any pertussis vaccination after the 2aP preschool booster dose, or after 6 years of age, emigration from Finland, death, or December 31, 2019, whichever came first. Competing risks were not considered.

For the primary effectiveness analysis, follow-up was additionally censored at the age of 9.25 years. For time since vaccination analysis, follow-up was stratified into periods: under 1 year, 1−<2 years, 2−<4 years, 4−<6 years, 6−<8 years, and 8 years or more from the booster vaccination to the end of follow-up. The adolescent booster is scheduled at the age of 14 years, but that did not affect this analysis, as by the end of the study period, the oldest children in our cohort were under 13 years of age.

For the analysis by previous vaccinations, follow-up for children contributing as booster-unvaccinated was additionally censored at the time of booster dose, and follow-up for children contributing as booster-vaccinated started on the date of their exposure (booster dose).

#### Exposure definition for the cohort design.

To define the exposure, information was retrieved from NVR, which has recorded all vaccinations administered in public primary health care and, increasingly, in private health care since 2009 and data is considered reliable since 2011. NVR was used to collect all individual pertussis vaccination records (administration date, vaccine, trade name) for the study children born in 2007 or later, through December 31, 2019.

Exposure in the cohort analyses was the preschool 2aP pertussis vaccination in combination with diphtheria, tetanus, and inactivated poliomyelitis vaccines, DT2aP-IPV (Tetravac), and occasionally, also with the Hib-component (Pentavac), used as a time-dependent variable. Children started the follow-up according to their vaccination status for the booster dose. If a vaccination occurred during follow-up, the child was then switched to the vaccinated cohort. Study subjects contributed to the follow-up time (i.e., time to event) according to their status. A child was considered vaccinated if more than 14 days had passed since their NVR record of the pertussis vaccine of interest.

The definition for the preschool vaccination was that a 2aP vaccine had been administered between 3 and 6 (less than 7) years of age. If a record of 2aP vaccination was followed by another record of 2aP vaccination within the first 31 days, the latter record was dropped. This was executed iteratively, starting from each child’s first 2aP vaccination record. If a child had received more than one 2aP vaccination between the age of 3 years and the start age of the follow-up, the last of the vaccinations was defined as the booster dose, and in this case, the child was considered vaccinated on the day of the latter vaccination dose rather than 14 days after it. If a child received no 2aP vaccinations between the age of 3 years and the start age of the follow-up, the first 2aP vaccination after the start age of the follow-up (4.25 years) was defined as the booster dose.

Previous (primary) vaccinations (doses 1–3) were defined as any full-strength (not low-dose) pertussis vaccine administered before 3 years of age. In addition, the second dose had to be administered ≥28 days after the first dose and the third dose ≥181 days after the second. However, if a record of a primary dose vaccination was immediately followed by another record of primary dose vaccination within 1 day, the latter record was dropped. Six subsets were analyzed: children with 0, 1–3, and 3 primary vaccinations with and without a booster. In each analysis, booster-vaccinated and booster-unvaccinated children, with different numbers of primary doses, were compared (relative effectiveness). Absolute effectiveness was defined as 3 primary vaccinations and a booster vaccination compared with those who received no pertussis vaccinations.

### Before-and-after study

**Fig 2:**
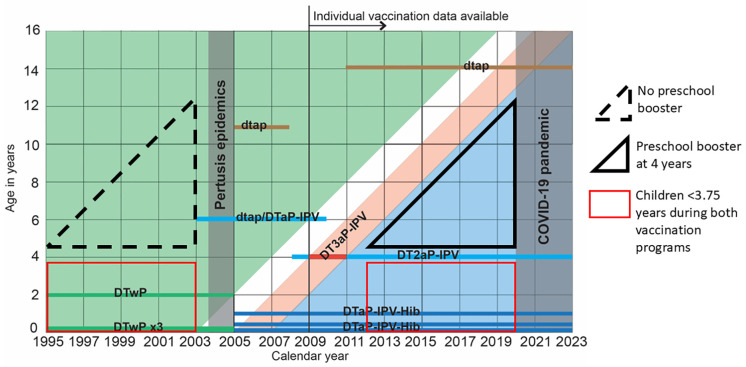
Before-and-after comparison for the impact of the current vaccination program including a 2-component aP preschool booster. The black triangles represent children aged 4.25 years, followed through to the end of each period and included in the assessment of the overall effect of the vaccination program. The red squares represent the children aged <3.75 years during both periods, included in the assessment of the indirect effect of the vaccination program. For details of the vaccination programs, see Table A in S1 File. Abbreviations: DTwP, combination vaccine containing whole-cell pertussis component; DTaP-IPV (+/- Hib), combination vaccine containing acellular pertussis components; 3aP, 3-component acellular pertussis vaccine; 2aP, 2-component acellular pertussis vaccine; dTap, low-dose combination vaccine containing acellular pertussis components.

**A before-and-after design** was used to estimate the impact of the 2aP preschool pertussis booster vaccine by comparing pertussis incidence rates between the old wP vaccination program without a preschool booster (1995–2002) and the current aP vaccination program with the preschool booster (2012–2019). Overall (direct and indirect) impact was assessed among individuals eligible for the 2aP booster dose, while indirect impact was assessed among individuals too young to receive the booster dose. (Fig 2)

#### Study setting and population for before-after design.

The before-and-after comparison comprised two periods, 2012–2019 and 1995–2002, representing vaccination programs with (“target cohort”) and without (“reference cohort”) the preschool booster dose, respectively. The population for the before-and-after analysis consisted of four separate Finnish child populations. For estimation of overall impact, the target cohort (children born on October 1, 2007 – December 31, 2015) were followed from the age of 4.25 years until December 31, 2019, and the reference cohort (children born on October 1, 1990 – December 31, 1998) were followed from the age of 4.25 years until December 31, 2002. For the estimation of indirect impact, the target cohort (children born on April 1, 2008 – December 31, 2019) were followed from January 1, 2012, until the age of 3.75 years or until the end of follow-up on December 31, 2019, and the reference cohort (children born on April 1, 1991 – December 31, 2002) were followed from January 1, 1995, until the age of 3.75 years or until the end of follow-up in December 2002. No censoring was considered in these analyses except for the end of follow-up.

Follow-up time was estimated using open aggregated population data from Statistics Finland. Since the data are end-of-year snapshots of population by birth year, the follow-up time over the relevant time periods and age groups could not be directly calculated. To obtain an estimate, each year each individual in a birth year cohort was assumed to reside in Finland for at least 1 full year, until their next birthday, with population changes between years assumed to occur evenly throughout the year.

Years 2003–2008 were excluded from the before-and-after analysis because of a transitional stage between the two vaccination programs with catch-up pertussis vaccinations at different ages. Specifically, an extra low-dose acellular pertussis booster dose was offered to children at 6 years of age since 2003 or at 11–13 years of age since 2005. In addition, exceptional pertussis epidemics occurred in 2003–2004. Years 2009–2010 were excluded because a 3-component acellular pertussis vaccine was used in the NIP instead of a 2-component vaccine. The year 2011 was excluded to make the two cohorts comparable in duration. Furthermore, years from 2020 onwards were excluded because of the COVID-19 pandemic mitigation measures, which strongly affected the occurrence of respiratory tract infections.

#### Exposures for the before-and-after design.

In before-and-after analyses, no individual vaccination data were used. Children belonging to the target cohort were defined as exposed to the 2aP booster vaccination program, while those in the reference cohort were classified as unexposed. The vaccine coverages for the three first pertussis vaccine doses were 89–99% and 98–100% in 2012–2019 and 1995–2002, respectively [10]. The coverages of the DTaP-IPV administered between 2–7 years of age in the birth cohorts 2009–2015 were 88–97% [11]. The coverages of the whole primary vaccination series by 2 years of age in the birth cohorts of 1995, 1997, 1999, and 2001 were 95–98% [12].

### Outcomes

Primary outcome: laboratory-confirmed pertussis was defined as a case of pertussis registered in the National Infectious Disease Register (NIDR) based on detection of *B. pertussis*, by polymerase chain reaction (PCR), culture, serology, or other methods.

During the time of our study, pertussis diagnostics in Finland mainly were based on serology, especially in children over 4 years of age. During the early years of the study period, serological tests mostly detected IgA and IgM with whole bacteria sonicates. Transition to pertussis toxin (PT)-IgG based serology took place during the 2010s. [6,13] There are recommendations, also applied in Finland, to not use serology for children vaccinated <1 year ago due to vaccine-induced antibodies, but in practice, the decision to test is at the discretion of the clinician, and adherence to this recommendation is inconsistent. [14] Reporting of positive laboratory findings of *B. pertussis* is mandatory for all laboratories in Finland by law. In PT-based methods, a single-sample serology is usually considered positive and notified to NIDR if IgG is high (>100 IU/ml) or moderately high IgG (40–100 IU/ml) coupled with a high titer for IgA antibodies. Paired-sample serology is usually considered positive and reported if there is a significant increase in IgG antibodies (e.g., 100%). [13] However, there might be some variation in the assay thresholds and notification practices by the laboratory.

#### Secondary outcomes.

A pertussis detection temporally related to hospitalization was defined as a laboratory confirmation date registered in NIDR within 14 days before or 7 days after an inpatient hospital admission recorded in the Care register for health care (HILMO).

Clinically diagnosed pertussis was defined as a record of ICD-9 code 0330A/B, or 0339A/B, ICD-10 code A37.0 or A37.9, or ICPC-2 code R71 in HILMO or the Register of primary health care visits (AvoHILMO), irrespective of laboratory confirmation.

Laboratory-confirmed adenovirus was defined as a case of adenovirus registered in the NIDR based on detection of adenovirus by antigen detection, PCR, serology, culture, or other methods.

### Demographics and covariates

Definitions of demographic and covariate variables are depicted in Table 1.

**Table 1:** Definitions of demographic and covariate variables related to a study subject.

Variable	Definition	Data source	Analysis/role
Year	Integer. Year of entering the study cohort	DVV	Covariate in cohort analyses
Sex	Factor with levels: male (reference), female, other. In cases of discrepant information in the registers, data from DVV was used.	DVV, NIDR, HILMO, AvoHILMO	Covariate in cohort analyses, strata variable in descriptive analyses
Region of residence	Factor with Finnish regions as levels: Ahvenanmaa, Etelä-Karjala, Etelä-Pohjanmaa, Etelä-Savo, Kainuu, Kanta-Häme, Keski-Pohjanmaa, Keski-Suomi, Kymenlaakso, Lappi, Pirkanmaa, Pohjanmaa, Pohjois-Karjala, Pohjois-Pohjanmaa, Pohjois-Savo, Päijät-Häme, Satakunta, Uusimaa ja Varsinais-Suomi. Determined at the index date for cohort analysis and at case occurrence for descriptive analysis.	DVV, NIDR, AvoHILMO, HILMO	Covariate in cohort analyses, strata variable in descriptive analyses
Number of chronic diseases	Number of relevant chronic diseases. Factor with levels 0 (reference), 1, ≥ 2. Determined at index date and daily during follow-up: reimbursement decisions effective at and/or after index date were considered. Thirteen different categories were defined based on reimbursement codes and attached diagnoses codes (S1 Appendix): lung disease, severe disorder of the immune system, diabetes, undernutrition, actively treated cancer, severe kidney disease, inflammatory bowel disease, adrenal insufficiency, other endocrinological disease, severe heart disease, neurological condition, severe mental disorders, and Down syndrome and severe disability	The Social Insurance Institution (KELA) Benefits register	Covariate in cohort analyses
Number of inpatient hospitalizations	Number of inpatient hospitalizations between 14 and 14 + 365 days before the index date and daily with a lag of 14 days during follow-up. Categories for the number of inpatient hospitalizations: 0 (reference), 1–4, ≥ 5.	HILMO	Covariate in cohort analyses
Foreign background	Boolean. TRUE if the country of birth of both parents or the only known parent was other than Finland. Children without parental data were considered foreign if they had no Finnish birth municipality, or if their native language was either missing or not an official language of Finland (Finnish, Swedish or Sami). Determined at index date.	DVV	Covariate in cohort analyses

### Statistical methods

R version 4.4.1 (R Foundation for Statistical Computing) [15] was used in all final data analysis. IBM SPSS Statistics, version 29.0, 2.0 was used for data exploration.

#### Study size.

This was a population-based study; the number of children eligible during our study period determined the sample size. Sample size calculations for the primary endpoint, laboratory-confirmed pertussis, are provided in S1 File.

#### Univariate (descriptive) analyses.

The distributions of the categorical variable values used as covariates in the regression models were compared between the unvaccinated and vaccinated members of the primary cohort at the start of the follow-up using the G-test.

Annual incidence was calculated by dividing the age-group and year-specific case numbers by the corresponding mid-year population, using Statistics Finland’s end-of-year population numbers [16] for two consecutive years, and expressed per 100 000 individuals.

#### Multivariable (comparative) analyses.

1. Vaccine effectiveness using a cohort design

The effect measure of interest was VE quantified as (1 - hazard ratio (HR)) * 100%. Cox proportional hazards model was used for estimation, treating vaccination as a time-dependent variable. The regression model adjusted for sex, region of residence, foreign background, number of chronic diseases, number of inpatient hospitalizations, and cohort entry year, using age as the time scale. Precision was expressed by 95% confidence intervals. Z-test was used when testing the equality of coefficients from two different models; statistical significance was deemed at p < 0.05. [17]

1. Vaccine impact using the before-and-after design

The effect measures of interest were the overall and indirect impact of the preschool booster program, quantified as (1 - incidence rate ratio (IRR)) * 100%. The incidences of the outcome of interest (all occurrences of an individual) between the target and reference cohorts were compared. Poisson regression was used for estimation with the log of follow-up time as an offset in the model. Precision was expressed as 95% profile-likelihood CIs. No covariate adjustments were used in the model.

1. Analysis by time since vaccination

Follow-up among vaccinated children was stratified by time since receipt of the booster vaccination: < 1, 1– < 2, 2– < 4, 4– < 6, 6– < 8, and ≥8 years. For each interval, the hazard of laboratory-confirmed pertussis was compared between children of the same age who received the preschool booster and those who did not.

#### Missing values.

Missing data are usually not considered in the Finnish register data. If a person lacked a record, such as a positive laboratory finding, diagnosis, prescription, or visit, he/she was considered not to have the laboratory-confirmed infection, exposure, outcome, comorbidity, treatment, or contact with a professional in question. Some variables occasionally had missing values.

Children residing in municipalities during a period when the number of reported infant DTaP-IPV-Hib vaccinations per capita was temporally lower than expected, likely due to issues in data transfer to NVR, were excluded from the cohort analysis. The adequacy of vaccination reporting was evaluated by reviewing the monthly pertussis vaccination counts for children under 2 years old in each municipality. Per capita doses were calculated using annual population figures from Statistics Finland. The periods of inadequate vaccination reporting were identified by observing atypically low vaccination numbers in consecutive months, based on visual inspection and considering the calendar time and the overall municipal reporting rate.

When missing data occurred, no imputation was typically performed, and all descriptive statistics were calculated using only non-missing values. In the DVV municipality history data, missing start dates were replaced with birth dates, if the birth municipality was in Finland. In case the birth municipality information was missing, the individual was considered not to have been born in Finland. The few and short gaps in municipality history were filled by extending the end of the previous period until the day before the start of the next period. If there was a gap between an individual’s birth date and the start date of their first record in the municipality’s history, they were interpreted to have lived abroad for that period.

#### Sensitivity analyses.

Several preplanned sensitivity analyses were conducted to validate the study results. First, the adenovirus infection recorded in the NIDR was used as a negative control outcome, both in a cohort and before–and–after comparisons, to assess residual confounding.

Second, we repeated the cohort analyses after excluding children with laboratory-confirmed pertussis and those with either laboratory-confirmed or clinically diagnosed pertussis before follow-up, to evaluate whether prior natural immunity influenced effectiveness estimates.

Third, a cohort analysis was performed on children born between March 1, 2007, and September 30, 2015, with follow-up starting at 3.75 years of age and redefining the booster dose accordingly, to test the effect of an earlier follow-up start.

Finally, vaccine effectiveness by prior vaccination status was re-estimated among children born in 2009 or later to assess whether potentially incomplete primary series reporting in the early years of the NVR influenced the results.

#### Additional analyses.

Unplanned additional analyses were performed to address further questions inspired by the results from the preplanned analyses. First, for all analyses of laboratory-confirmed pertussis, follow-up was split into the first year after vaccination and the rest of follow-up to explore and isolate the potential effect of vaccine-induced antibodies on serological tests within one year after vaccination and the apparent excess of false positives during that time period. [14]

Second, two approaches of the primary cohort analysis were conducted using pertussis confirmed by (a) culture/PCR only and (b) serology only. The same stratification by time since vaccination was repeated for these outcomes to explore the impact of misclassification in serological tests and potential differences in estimates across diagnostic methods.

Third, absolute effectiveness of the full 3-dose (2 + 1) primary vaccination series against laboratory-confirmed pertussis and adenovirus compared with no primary doses. Exposure was 3 primary doses, as defined in the section ‘Exposures for the cohort analysis’. Follow-up was from the age of 30 days until December 31, 2019, or censoring event. An additional exclusion criterion was receiving any pertussis vaccination other than the primary series dose before the age of 30 days. Additional censoring events were a primary series dose (1–3) vaccination with an insufficient break after the previous dose (as defined for the analysis by previous vaccinations) and any pertussis vaccination other than the first, second, or third primary dose. In addition, the follow-up of a child who received at least one primary dose was suspended on the day of their first primary dose vaccination and continued once they became vaccinated with the third primary dose, if applicable. Three sub-analyses were conducted: VE against pertussis by age and by time since vaccination and VE against adenovirus by age as a negative control, to explore the effects of the primary series itself and aid interpretation of the main results.

Fourth, cohort analysis on vaccine effectiveness against any laboratory confirmed pertussis was conducted with the follow-up stratified based on age in years, as a sensitivity analysis for effectiveness by time since vaccination.

Finally, the primary cohort analysis was repeated without excluding children from areas with inadequate vaccine reporting. In the protocol this exclusion criterion was intended only in the primary analysis, but because removing the exclusion criterion seemed to result in underestimation of the VE, it was used in all cohort analyses.

## Results

### Descriptive data

**Fig 3:**
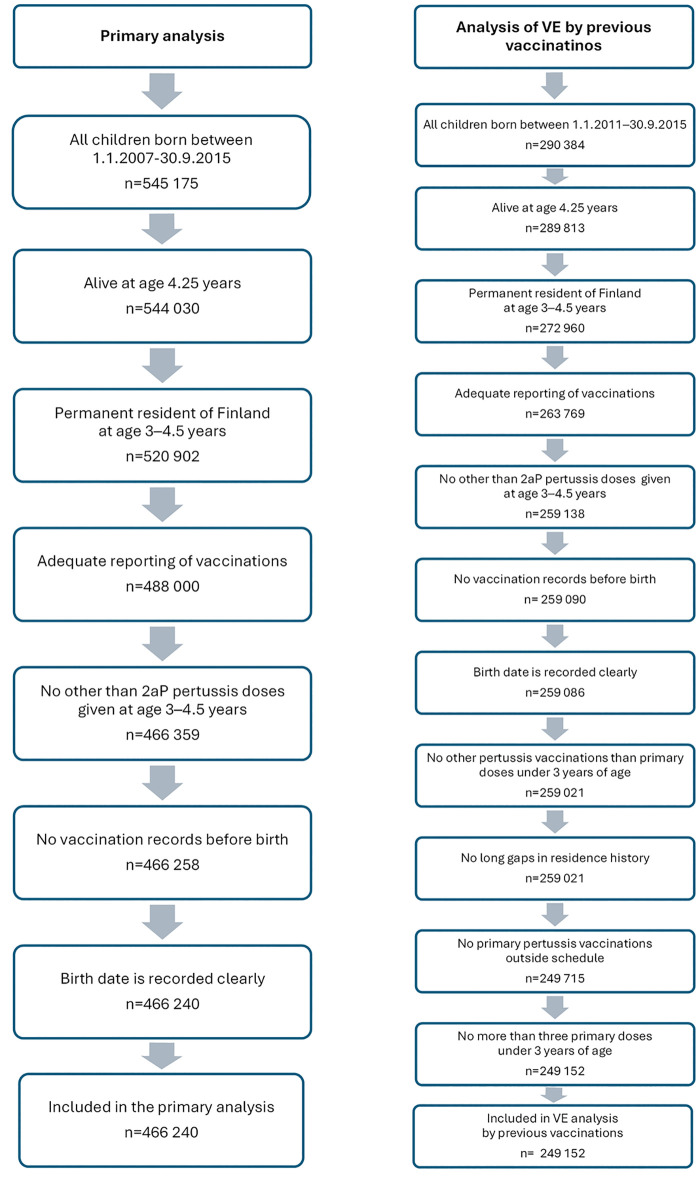
Selection of children into the primary analysis and effectiveness analysis by previous vaccination status, after exclusions.

The selection of the study population for the primary cohort analysis and analysis by previous vaccinations after applying exclusion criteria is depicted below in Fig 3. Of all the children eligible based on birth cohort, 85.5% were included in the primary cohort analysis, and 86.0% in the analysis by previous vaccinations after applying exclusion criteria. The numbers of subjects included in other analyses are presented in the result tables.

In the total study population during the years 1995–2019, there were 6 980 laboratory-confirmed pertussis cases and 12 444 laboratory-confirmed adenovirus cases. During the 1 615 237 person-years of follow-up (median 3.5, maximum 5 years) in the primary cohort analysis, 225 laboratory-confirmed pertussis cases were identified. Of cases, 88% (196 − 197/225) were diagnosed with serological tests, while only 12% (27/225) were diagnosed with culture or PCR tests.

**Fig 4:**
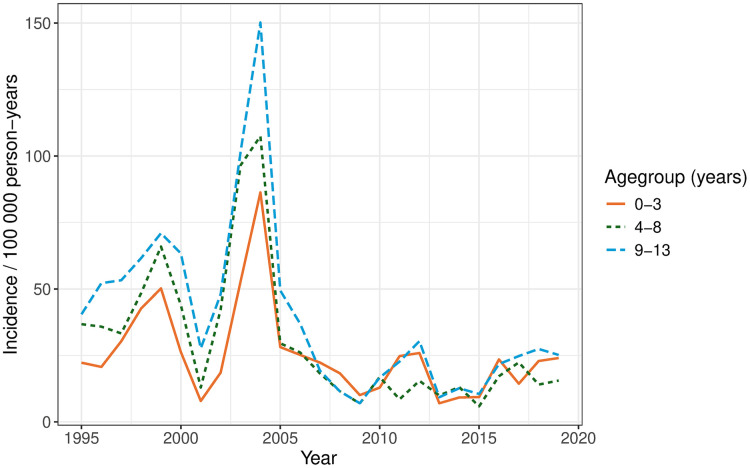
Incidence of laboratory-confirmed pertussis among all study children during 1995–2019 by age-group (years). Data sources: Digital and Population Data Services Agency, National Infectious Disease Register.

The incidence rates of laboratory-confirmed pertussis for all age groups during 1995–2019 are depicted in Fig 4. The findings were based on PCR or culture in 16% (454/2905) of cases in the study period 1995–2002 and in 25% (317/1290) of cases in the study period 2011 − 2019. Other cases were diagnosed mainly by serology (Table A in S2 Appendix).

In addition to *B. pertussis,* laboratory findings of *B. parapertussis* were extracted from the NIDR to explore the potential misdiagnosis of pertussis and the overall epidemiology of *B. parapertussis*. Only 28 laboratory-confirmed cases were found during the study period of 1995 − 2019. The analysis was not performed due to low number of records.

### Cohort sociodemographic data

In the primary cohort analysis, the children with foreign background were overrepresented among those without the preschool booster (Table 2). There were some temporal and spatial differences in the vaccination rates. The low proportion of vaccinated participants entering the study in 2011 was partly explained by the fact that some children had received a 3aP booster vaccine, used in the NIP until 2010. These children were excluded from the analyses.

**Table 2:** Demographics by preschool booster status for children aged 4.25–9.25 years and born between January 1, 2007– September 30, 2015 (Cohort study).

Characteristic	Distribution of regression model covariates presented as a proportion (%) of total follow-up		G-test of independence (test value)
	Without 2aP preschool booster (103 751 follow-up years)	With 2aP preschool booster (1 511 486 follow-up years)	
**Number of chronic conditions**			<0.001
0	97.2	97.2	
1	2.6	2.7	
>1	0.3	0.1	
**Number of hospitalizations**			<0.001
0	96.2	97.1	
1–4	3.4	2.8	
>4	0.3	0.1	
Sex			<0.001
Female	48.5	48.9	
Male	51.5	51.1	
**Region of residence**			<0.001
Ahvenanmaa (Åland)	0.1	0.03	
Etelä-Karjala	1.8	2.2	
Etelä-Pohjanmaa	2.5	3.5	
Etelä-Savo	1.6	2.2	
Kainuu	0.8	1.0	
Kanta-Häme	2.2	2.6	
Keski-Pohjanmaa	1.4	1.7	
Keski-Suomi	4.4	5.4	
Kymenlaakso	2.3	2.7	
Lappi	2.9	3.3	
Pirkanmaa	10.8	8.7	
Pohjanmaa	4.8	3.5	
Pohjois-Karjala	2.8	2.5	
Pohjois-Pohjanmaa	8.3	9.1	
Pohjois-Savo	2.7	4.2	
Päijät-Häme	5.5	3.7	
Satakunta	3.0	4.1	
Uusimaa	34.3	31.3	
Varsinais-Suomi	7.7	8.4	
**Foreign background**			<0.001
No	84.1	94.0	
Yes	15.9	6.0	
**Year of entering the study**			<0.001
2011	10.3	7.6	
2012	12.7	16.2	
2013	12.9	16.7	
2014	11.9	17.4	
2015	15.3	15.5	
2016	18.4	11.7	
2017	9.1	8.4	
2018	6.8	4.9	
2019	2.7	1.6	

For G-test, the covariate values were measured at the start of follow-up.

Data sources: Digital and Population Data Services Agency, National Vaccination Register, Care Register for Health Care, The Social Insurance Institution (KELA) Benefits register.

Primary and booster vaccinations were mostly administered according to the NIP schedule (Table C in S2 Appendix). The vaccination coverage was high: 92% of children received the complete primary series, and 93% were booster vaccinated (irrespective of the previous doses) among the children eligible for all doses during the study period. In the primary analysis cohort, 94% (436 845/466 240) were booster vaccinated.

### Vaccine effectiveness

The vaccine effectiveness estimates reported in the text are adjusted for age, sex, region of residence, foreign background, number of chronic diseases, number of inpatient hospitalizations, and year of entering the study cohort, unless otherwise stated. These potential confounders available in the national registers were chosen because they were expected to reflect the children’s susceptibility to illness and affect their level of infection pressure. The unadjusted estimates can be found in the tables.

#### Effectiveness in children 4.25 − 9.25 years of age and time since vaccination.

In the primary analysis, the effectiveness of the preschool booster was 51% (95% CI 27–67%) against any laboratory-confirmed pertussis among children aged 4.25–9.25 years (Table 3).

**Table 3:** Effectiveness of 2aP preschool booster against pertussis and adenovirus among children over 4.25 years old and born between January 1, 2007 – September 1, 2015, in 2011–2019 — Cohort study.

Outcome	Without 2aP preschool booster (reference)			With 2aP preschool booster			VE (95% Cl)	
	N	Follow-up (years)	Cases, n (incidence per 100 000 person-years)	N	Follow-up (years)	Cases, n (incidence per 100 000 person-years)	Unadjusted	Adjusted
Primary analysis: Laboratory-confirmed pertussis among children aged 4.25–9.25 years	85 421	103 751	31 (29.9)	436 845	1 511 486	194 (12.8)	55 (34–70)	51 (27–67)
Pertussis detected only with culture/PCR	85 421	103 791	9 (8.7)	436 852	1 511 859	18 (1.2)	89 (74–95)	86 (67–94)
Pertussis detected only with serology	85 421	103 759	22 (21.2)	436 845	1 511 502	175 (11.6)	41 (7–62)	37 (-1–60)
								
Laboratory-confirmed pertussis* by time since vaccination	85 421	116 370	38 (32.7)	–	–	–	–	–
<1 year	–	–		436 604	354 538	59 (16.6)	41 (-10–68)	37 (-15–66)
1 – < 2 years	–	–		390 708	362 892	23 (6.3)	73 (42–87)	71 (38–86)
2 – < 4 years	–	–		337 547	570 401	78 (13.7)	61 (34–76)	58 (30–75)
4 – < 6 years	–	–		234 414	360 343	56 (15.5)	58 (19–78)	55 (13–77)
6 – < 8 years	–	–		127 837	152 938	25 (16.4)	75 (41–89)	72 (34–88)
≥8 years	–	–		27 491	9 490	5 (52.7)	–337 (-1111 to -58)	–383 (-1292 to -68)
Pertussis detected only with culture/PCR	85 421	116 428	10 (8.6)	–	–	–	–	–
<1 year	–	–	–	436 611	354 571	3 (0.9)	73 (15–92)	70 (1–91)
1 – < 2 years	–	–	–	390 764	–	1–2 (0.3)	96 (67–99)	95(59–99)
2 – < 4 years	–	–	–	337 613	570 573	10 (1.8)	88 (58–96)	85 (50–95)
4 – < 6 years	–	–	–	234 515	360 540	5 (1.4)	86 (14–98)	84 (4–97)
6 – < 8 years	–	–	–	127 932	153 065	3 (2.0)	91 (53–98)	91 (46–98)
≥8 years	–	–	–	27 523	–	1–2 (10.5)	–69 (-1008–74)	–79 (-1283–77)
Pertussis detected only with serology	85 421	116 383	28 (24.1)	–	–	–	–	–
<1 year	–	–	–	436 604	354 539	56 (15.8)	35 (-30–68)	32 (-35–66)
1 – < 2 years	–	–	–	390 710	362 893	22 (6.1)	64 (16–85)	62 (12–84)
2 – < 4 years	–	–	–	337 548	570 409	68 (11.9)	45 (6–68)	42 (1–66)
4 – < 6 years	–	–	–	234 419	360 358	50 (13.9)	47 (-13–75)	43 (-21–73)
6 – < 8 years	–	–	–	127 844	152 948	22 (14.4)	67 (10–88)	62 (-2–86)
≥8 years	–	–	–	27 493	9 490	4 (42.2)	–494 (-1825 to -83)	–571 (-2168 to -99)
								
Laboratory-confirmed pertussis temporally related with hospitalization*	85 421	–	1–2 (0.9)	436 852	1 811 227	4 (0.2)	78 (-125–98)	80 (-88–98)
Clinically diagnosed pertussis*	85 421	116 426	16 (13.7)	436 844	1 810 861	148 (8.2)	41 (2–64)	40 (-1–64)
Laboratory-confirmed adenovirus among children aged 4.25–9.25 years (negative control)	85 421	103 674	76 (73.3)	436 838	1 510 300	758 (50.2)	24 (4–40)	15 (-8–33)

*Follow-up from age 4.25 to the end of the follow-up. Frequencies of 1 or 2 are masked following the data privacy policy of Findata.

Data sources: Digital and Population Data Services Agency, National Infectious Disease Register, National Vaccination Register, Care Register for Health Care, Register of Primary Health Care Visits, The Social Insurance Institution (KELA) Benefits register.

When stratified by time since vaccination, VE was 37% (95% CI −15–66) within the first year and ranged from 58% to 75% during years 1–8 post-vaccination, with overlapping confidence intervals. The unexpectedly low estimate in the first year likely reflects false-positive serology results due to vaccine-induced antibodies, prompting unplanned additional analyses. In these analyses, the effect of potential serology-related false positives was evident as VE calculated using only PCR or culture diagnoses over a 5-year follow-up was significantly higher at 86% (95% CI 67–94%), compared to 37% (95% CI −1–60%) for diagnoses based solely on serology (p = 0.019, z-test for difference). Across time intervals, VE estimates based on serology were consistently lower than those based on PCR/culture (Table 3). Regardless of diagnostic method, no waning of effectiveness was found by 8 years post-vaccination.

Against clinically diagnosed pertussis, VE was 40% (95% CI −1–60%), just below the threshold for statistical significance. Effectiveness against adenovirus infection, used as a negative control, was low and not statistically significant (15%, 95% CI −8–33%).

**Fig 5:**
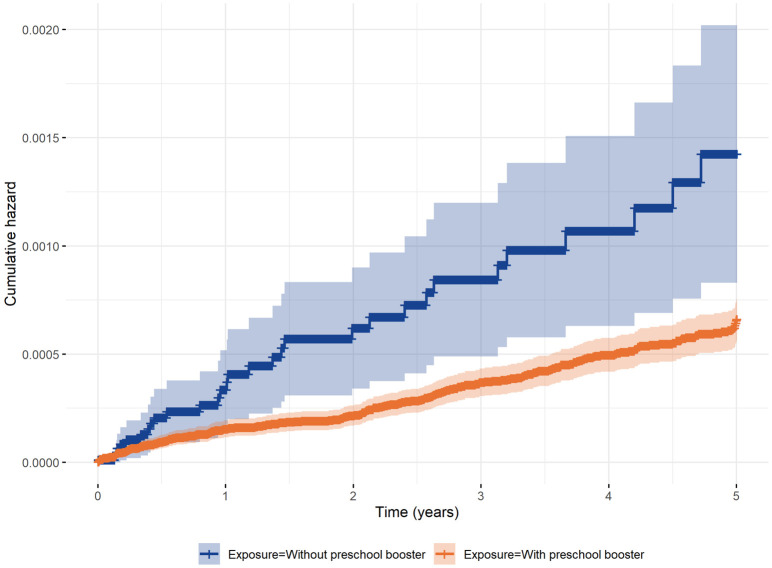
Cumulative hazard of laboratory confirmed pertussis ─ primary cohort analysis.

For the primary analysis, the proportional hazards assumption between the children with and without the preschool booster was assessed by visually inspecting the cumulative hazard curve (Fig 5) and by testing the scaled Schoenfeld residuals (Fig B in S2 Appendix). The assumption of proportionality was deemed to hold.

#### Results by previous vaccination status.

In the analysis by previous vaccination status (Table D in S2 Appendix), the absolute VE for the full series (3 primary doses and the preschool booster dose) vs. fully unvaccinated (no primary doses and no booster) was 88% (95% CI 74–95%). After the first year since vaccination (VE 79%, 95% CI 41–92%), the point estimate was even higher at 93% (95% CI 83–97%). Relative effectiveness of the full series (3 primary doses and the preschool booster dose) compared to only 3-dose primary series (3 primary doses and no preschool booster dose) was lower at 11% (95% CI −103–61%), but 51% (95% CI −28–81%) when limited to more than one year since vaccination. This analysis was limited due to a very small number of cases, low number of children without the preschool booster and shorter follow-up.

### Impact of booster vaccination

Overall (combined direct and indirect) impact of 2aP booster vaccination on laboratory-confirmed pertussis cases was estimated as a reduction of 65% (95% CI 60–69%) in incidence rates among children over 4.25 years of age between the target and reference vaccination programs (Table 4). The estimated impact was clearly higher for the age group between 6–13 years of age compared to children 4.25–6 years of age. No covariate adjustments were applied in the model used to assess the impact of vaccination.

**Table 4:** Association of 2aP preschool booster with reduced incidence of pertussis among children in 1995–2002 and 2012–2019 — before-and-after comparison.

Group	1995–2002 (reference)			2012–2019 (target)			Impact of preschool booster (95% CI)
	Cases	Follow-up (years)	Incidence per 100 000 person-years	Cases	Follow-up (years)	Incidence per 100 000 person-years	
Children born October 1990–1998 (reference) or October 2007–2015 (target) — overall impact							
Children aged ≥ 4.25 years	871	2 079 861	41.9	289	1 970 635	14.7	65 (60–69)
4.25 – < 6 years old	225	796 641	28.2	109	758 859	14.4	49 (36–60)
6 – < 8 years old	324	684 864	47.3	88	648 982	13.6	71 (64–77)
8 – < 13 years old	322	598 357	53.8	92	562 794	16.4	70 (62–76)
Children aged 4.25 – < 9.25 years	730	1 780 573	41.0	239	1 690 197	14.1	66 (60–70)
Children born in April 1991–2002 (reference) or born in April 2008–2019 (target) — indirect impact							
Children aged ≤ 3.75 years	503	1 809 703	27.8	288	1 710 350	16.8	39 (30–48)
≤ 2 years old	367	948 477	38.7	216	888 077	24.3	37 (26–47)
>2 – ≤ 3.75 years old	136	861 225	15.8	72	822 272	8.8	45 (26–59)
Target years 2012–2015*	503	1 809 703	27.8	117	898 862	13.0	53 (43–62)
Target years 2016–2019*	503	1 809 703	27.8	171	811 488	21.1	24 (10–36)

Compared to the whole reference period in children aged ≤ 3.75 years.

Frequencies of 1 or 2 are masked following the data privacy policy of Findata.

Data sources: Digital and Population Data Services Agency, National Infectious Disease Register.

Indirect impact of the booster vaccination was estimated as a reduction of 39% (95% CI 30–48%) in the incidence rates among children under 3.75 years of age, not yet eligible to receive the booster dose according to the vaccination program. Impact estimates did not significantly differ by age. The impact estimate for the years 2012–2015 was higher (53%; 95% CI 43–62%) than for the years 2016–2019 (24%; 95% CI 10–36%)*.*

### Other analyses

In the additional analysis of the effectiveness of the full 3-dose (2 + 1) primary vaccination series without booster against any laboratory-confirmed pertussis, the effectiveness estimates were high at 92% (95% CI 85–96%) for 0–3 years of age, and 81% (95% CI 51–93%) for children over 4 years old (Table 5). Effectiveness estimates in time intervals since vaccination were also high and showed only a small trend of waning (Table 5). Of the primary doses, 74% were 3aP, 23% 2aP, and 3% were aP vaccines with tradename not known.

**Table 5:** Absolute effectiveness of the full primary series against laboratory-confirmed pertussis and adenovirus among children born between January 1, 2011 – Sep 1, 2015, in 2011–2019 — cohort design.

Outcome	Without any primary or booster doses (ref)			With full primary series and no booster dose			VE (95% Cl)	
	N	Follow-up (years)	Cases, n (incidence per 100 000 person-years)	N	Follow-up (years)	Cases, n (incidence per 100 000 person-years)	Unadjusted	Adjusted
Laboratory-confirmed pertussis	266 870	73 288	91 (124.2)	–	–	–	–	–
<1 year since last vaccination	─	─	58.8	236 586	235 951	10 (4.2)	85 (-60–99)	84 (-64–99)
1 – < 3 years since last vaccination	─	─	76.5	235 728	460 797	32 (6.9)	94 (88–97)	93 (87–97)
3 – < 7 years since last vaccination	─	─	110.5	108 799	43 319	10 (23.1)	81 (50–92)	81 (50–92)
at 0–3 years old	–	–	88.9	245 495	695 546	42 (6.0)	93 (86–96)	92 (85–96)
at ≥4 years old	–	–	89.6	198 938	55 151	11 (20.0)	81 (52–93)	81 (51–93)
Laboratory-confirmed adenovirus (negative control)	266 870	73 308	94 (128.2)	–	–	–	–	–
at 0–3 years old	–	–	184.6	245 082	692 128	1 355 (195.8)	–9 (-62–26)	–2 (-51–32)
at ≥4 years old	–	–	172.7	197 518	54 711	55 (100.5)	–84 (-518–45)	–78 (-501–47)

Frequencies of 1 or 2 are masked following the data privacy policy of Findata.

Data sources: Digital and Population Data Services Agency, National Infectious Disease Register, National Vaccination Register, Care Register for Health Care, The Social Insurance Institution (KELA) Benefits register.

Results from other exploratory and sensitivity analyses are depicted in S2 Appendix.

## Discussion

### Key results

This is the first study to provide real-world evidence of aP vaccine effectiveness in Finland, and specifically the first to evaluate the real-world effectiveness of the 2aP vaccine as a preschool booster globally.

In the primary cohort analysis, the DT2aP-IPV preschool booster was found to be effective against laboratory-confirmed pertussis with a vaccine effectiveness of 51% (95% CI: 27–67%). When restricting the analysis to PCR/culture-confirmed cases, VE was clearly higher at 86% (95% CI 67–94%). Results by time since vaccination suggested that serological tests performed within one-year post-vaccination may have introduced false positives, thereby lowering VE estimates. Importantly, effectiveness remained consistent during the first 8 years following vaccination. When stratified by previous vaccination status, the absolute effectiveness of the preschool booster combined with the 3-dose (2 + 1) primary vaccination series was 88% (95% CI 74–95%), indicating high protection.

### Interpretation

The primary analysis comparing children with and without the preschool booster indicated moderate effectiveness against any laboratory-confirmed pertussis. Analyses comparing the full vaccination series (3-dose (2 + 1) primary series and preschool booster) and the 3-dose (2 + 1) primary series with unvaccinated showed high absolute vaccine effectiveness, consistent with previous literature [2,18]. In a German case-cohort study, absolute VE for the full 5-dose series (3 + 1 primary series and a preschool booster at age of 5) was 93% and 88% for four doses (received in the first 14 months) [19], corresponding to roughly 40% relative effectiveness of the booster, closely matching our findings.

We observed high VE against culture- or PCR-confirmed pertussis, consistent with prior research, where outcomes were mainly PCR/culture-confirmed [2,18]. VE was consistently higher against PCR/culture-confirmed cases than against serology-based ones, not only in the first year but across all time intervals. Similar findings were reported in a study on adult aP boosters [20]. Culture and PCR are diagnostically more specific for infection than serological tests even after the first year since vaccination and with a high cut-off point, due to false positives in antibody tests by high persisting antibodies from past infections, vaccinations and subclinical antibody responses, which could bias the serology-based estimates toward the null and explain the difference in the estimates. Further, since the target populations for these diagnostic tests are likely different (PCR is used in more acute settings vs serology is applied in prolonged cases), the prior probability of disease in the serologically tested population might be lower, potentially leading to more false positives by serology, even if the diagnostic specificity of serology would be comparable to PCR. The difference in the estimates may also indicate greater vaccine protection against severe disease, since PCR and culture are more used in hospital settings, whereas serology is used more in primary care for milder cases.

As shown in the results (Table 3), we found sustained effectiveness for 8 years after vaccination, contradicting the assumption that rapid waning drives pertussis resurgence. Comparable long-lasting protection has been observed in some older and recent studies [21–23]. Data from the Netherlands with a similar vaccination program indicate a decline around ages 9–11 years [24]. By contrast, a 2015 meta-regression suggested significant waning within a few years after vaccination, although most included studies lacked vaccine-naïve comparison groups [25]. Modeling studies have shown that higher infection risk in school-aged children can be explained by contact patterns and suboptimal vaccination coverage rather than waning [26,27]. Although our data showed sustained effectiveness over eight years, the confidence intervals do not exclude a gradual decline.

Another interesting finding in the results of VE by time since vaccination was the low estimates for the period of <1y since vaccination. We think this is mostly due to false positives caused by vaccine-induced antibodies in the serological tests. [14,28,29] Nevertheless, the first-year estimate was slightly lower in the estimates by PCR/culture as well, but this was not statistically significant so could have been due to chance alone as the number of cases was quite low. Further, there seems to be substantial residual protection from the 2 + 1 primary series, which could also explain some of the low VE estimates during the first year after vaccination.

The before-and-after comparison suggests that introducing the preschool booster substantially reduced pertussis incidence. The observed reduction among children too young for the booster suggests decreased transmission, consistent with findings from the Netherlands [30] and other epidemiological studies [31,32]. However, our impact estimates may have been influenced by the different primary series vaccines, vaccination schedules, additional adolescent and adult boosters between the two periods, along with changes in diagnostics and other possible temporal shifts in pertussis epidemiology.

Our study carries implications for future research and health policy. Although we observed long-lasting protection, our study period ended before the COVID-19 pandemic. Recent pertussis epidemics have shown increased incidence among adolescents [33] and a reduced proportion of pertactin-deficient strains [34], warranting further investigation of the preschool and adolescent booster effectiveness during the recent epidemics. Future studies should also account for misclassification in serology-based outcomes. Based on our findings of durable protection, no major changes to current Finnish or comparable vaccination programs—such as additional boosters—can be recommended. However, based on our results showing high and durable protection from the 3-dose (2 + 1) primary series alone, the preschool booster might be scheduled a bit too early from the perspective of pertussis protection. In many European countries, the preschool booster is scheduled later at 5–7 years of age [35], such as the Netherlands, where the preschool booster was postponed from the age of 4 to age of5 in 2025 [36]. Overall, these results are generalizable to other countries using similar aP vaccination programs for children.

### Strengths and limitations

This study has several important strengths. First, the nationwide population-based design, which included nearly all eligible children, ensured negligible loss to follow-up through comprehensive registers, minimized selection bias, and enhanced generalizability. Second, a large sample size reduced random error and, together with extensive covariate data, enabled controlling for many potential confounders in the adjusted Cox regression model. Third, sensitivity analyses using alternative definitions of exposure, outcomes, and follow-up produced minimal changes in the results, indicating good internal validity. The use of adenovirus infection as a negative control outcome further suggested limited residual confounding. Fourth, the Finnish register data are comprehensive and highly reliable. Nearly all childhood vaccinations are administered through the public system and reported to the NVR; municipalities with incomplete reporting were excluded. Outcome ascertainment was strengthened by using primarily laboratory-confirmed cases, with mandatory reporting of all positive results to the NIDR.

Nevertheless, several limitations should be acknowledged. The number of pertussis cases in our primary cohort was low, leading to wide confidence intervals and potential random error, substantially affecting estimates of relative effectiveness by number of previously received doses. However, estimates for the primary and most secondary objectives remained statistically significant. Given the very high pertussis vaccine uptake in the study population, the small unvaccinated group likely differed from vaccinated individuals in ways that could affect VE estimates and not be fully measured or controlled, such as healthcare-seeking behavior. One potentially important confounder was primary vaccination status, which was unavailable for the entire cohort because NVR records began in 2009, and the first two years (2009–2010) could be unreliable. If the booster-vaccinated children in the primary cohort were also more likely to receive and complete the 2 + 1 primary vaccination series, they would have a better protection from pertussis already before the preschool booster, and this could lead to an overestimation of the booster’s effect. In the subsample where primary vaccination information was known, among children with exactly three primary doses (2 + 1 schedule), the estimated relative effectiveness of the preschool booster was only 11% (95% CI −103–61%), but the analysis was substantially underpowered due to low number of children with only 3 primary doses. Further, the estimate for ≥1 year since vaccination was comparable to the estimate of the primary analysis, suggesting that bias from false positive outcomes during the first year after vaccination had a greater influence in this analysis, as the shorter follow-up period increased the relative contribution of events occurring during the first year.

False positives from imperfect diagnostic tests are another important limitation. Most of the diagnostics in our study population relied on serology, which has limited diagnostic specificity for infection, even with high cut-off point, and especially within 1 year of vaccination, as the tests cannot distinguish between infection-induced and vaccine-induced antibodies. [14,28,29] Many positive serological tests during this period likely caused differential misclassification, lowering first-year effectiveness estimates. We therefore stratified results by time since vaccination (<1 year and ≥1 year), which reduced bias but also statistical power. Additional false positives may have arisen from high persisting antibody titers after past infections, which also could have been subclinical or simply anamnestic responses to exposure to pertussis without transmissible infection. No data was available on the number of paired samples, but these were presumed to comprise only a few percent of all serological tests based on enquiries from the biggest laboratories. PCR and culture are more specific but were used infrequently, possibly due to the timing of sample collection, and limited availability and knowledge of the primary care physicians on the use of PCR diagnostics. Assuming non-differential misclassification, imperfect test specificity would bias effectiveness estimates toward the null.

Some pertussis cases likely remain undiagnosed. However, if under-ascertainment of the outcome is non-differential with respect to exposure, low outcome sensitivity should only slightly bias results toward the null. Differential testing by vaccination could occur, especially in severe cases, which would, in turn, overestimate VE. Conversely, if vaccinated individuals seek health care and get tested more often, this could bias the effectiveness estimate towards negative VE.

Some potential mechanisms could have biased VE estimates over time. First, if estimated VE improved over calendar years due to factors such as greater diagnostic specificity, later-year estimates could appear inflated. Although test-type distributions remained stable, serological assays evolved: Finnish laboratories transitioned during the 2010s from in-house tests to PT-IgG–based methods recommended by ECDC [13,14,37]. These newer assays are likely to be more specific, possibly reducing misclassification and slightly inflating later VE estimates, compared to earlier ones. Second, residual confounding may have changed over time because booster vaccination was modeled as a time-dependent exposure, and some children received the booster later than scheduled. Consequently, children contributing person-time to the unvaccinated group early in follow-up may have differed systematically from those remaining unvaccinated later. In particular, if children with greater residual protection from earlier pertussis vaccinations or otherwise lower underlying risk of pertussis were more likely to transition to the vaccinated group over time, VE may have been overestimated in later years. Conversely, if children who remained unvaccinated were less likely to seek healthcare and undergo testing, pertussis cases may have been underdetected, leading to a downward bias of the VE estimate.

Third, differential depletion of susceptibles may also bias VE estimates towards negative values over time [38]. This mechanism could have contributed to the negative point estimate −383% (95% CI −1292 to −68%) among children followed beyond 8 years. However, explaining an estimate of this magnitude would require a large burden of unobserved infections and strong differential natural immunity between vaccination groups.

Given that the negative estimate was based on very few cases, it should be interpreted with caution. As such a sudden change in VE from positive to clearly negative seems implausible, the estimate is more likely to reflect combination of statistical random error and potential residual bias rather than true negative effectiveness. Still, the result limits interpretation of duration of protection beyond 8 years since the last dose.

The before-after comparison is limited by the use of two different time periods, during which factors other than the implementation of a preschool booster may also have influenced pertussis epidemiology. As there is no parallel comparison population without an implementation of a vaccination program, the natural trends in pertussis epidemiology are not known. Differences in the diagnostic practices between the two study periods may have influenced the observed incidence of pertussis. Multiplex PCR panels became available in 2009 [39], increasing PCR-based diagnostics from 16% in 1995–2002 to 25% in 2011–2019. Nevertheless, despite improved diagnostics, the incidence of pertussis was clearly lower in the later period among children both younger and older than the scheduled age for the preschool booster. Changes in serological assays may likewise have affected the observed incidence, although the comparative sensitivity and specificity of the older and current tests are not known. In addition, changes in the physicians’ testing activity may have influenced the results. However, data on the number of pertussis laboratory tests performed were unavailable, as only positive test results are reported to the NIDR.

Another limitation in the before-and-after comparison is uncertainty about the effectiveness of the wP vaccines in the earlier period. No effectiveness studies have been conducted with the Finnish wP vaccines and estimates from previous studies on effectiveness of other wP vaccines vary widely. [2] While wP vaccines of European origin and aP vaccines administered in the primary series have long been assumed to be similarly effective, part of the observed impact could reflect higher effectiveness of the current primary vaccination series. In addition, dTap booster doses at the age of 14 (from 2005), for conscripts (from 2012), and at the age of 25 (from 2018) may have influenced the results by reducing household transmission. However, previous studies suggest adolescent boosters have little impact on infant cases in settings with demographics comparable to those of Finland. [40–42] Further, transmission models and studies imply low transmission between adolescents and infants. [43,44] Also, adult booster coverage remains low [45], and the late implementation compared with the end of our study period means that impact of the adult booster is likely minimal.

## Conclusion

DT2aP-IPV as a preschool booster appears to extend and sustain the protection conferred by the primary series against pertussis from school entry into adolescence. These findings support the continued use of the 2aP preschool booster in Finland.

## Supporting information

S1 File Study Protocol. (DOCX)

S1 Appendix Definitions for chronic diseases as a covariate. (DOCX)

S2 Appendix Results from other exploratory and sensitivity analyzes. (DOCX)

S1 Checklist STROBE checklist Completed STROBE checklist. The STROBE Statement checklist is reproduced under the terms of the Creative Commons Attribution 4.0 International (CC BY 4.0) License. See the STROBE Initiative for further information.
(DOCX)
