# Supplementary material for: Effectiveness and impact of the 2-component acellular pertussis vaccine as a preschool booster in Finland – A register-based study
Source: PLOS Glob Public Health. 2026 Jul 29;6(7):e0006800. doi: 10.1371/journal.pgph.0006800 (PMC13419238; doi:10.1371/journal.pgph.0006800)
Supplement: S1 File — (DOCX) [file pgph.0006800.s001.docx]

Protocol code **PER000108** FVR – Finnish Vaccine Research

**Name of the study: Effectiveness and impact of a 2-component acellular pertussis vaccine as a preschool booster in Finland – A register-based study**

**Finnish name of the study: Leikki-iässä annetun hinkuyskärokotteen tehokkuus ja vaikuttavuus Suomessa - rekisteripohjainen tutkimus**

**EU PAS Register number:** EUPAS1000000107

Version 2.0, date June 30, 2025

Adjusted in July 14, 2026 by changing Table and Figure names according to PLOS GPH requirements for Supporting information

**Investigator institute/Sponsor:** FVR-Suomen rokotetutkimus Oy (FVR – Finnish Vaccine Research Ltd) <https://fvr.fi/en/>

**FVR study team, all affiliated at the Real-Word Evidence (RWE) unit :**

| Palmu Arto | Principal investigator, chief research officer, scientific evaluation | arto.palmu@fvr.fi |
| --- | --- | --- |
| Syrjänen Ritva | Project manager, senior researcher, scientific evaluation | ritva.syrjanen@fvr.fi |
| Hanna Rinta-Kokko | Senior data analyst, scientific evaluation, statistical analyses | hanna.rinta-kokko@fvr.fi |
| Kimmo Niinimäki | Data analyst | kimmo.niinimaki@fvr.fi |
| Nieminen Heta | Senior researcher, scientific evaluation | heta.nieminen@fvr.fi |
| Isosaari Elina | Clinical team leader | elina.isosaari@fvr.fi |
| Kyrölä Maila | Study nurse, Findata contact person | maila.kyrola@fvr.fi |

**Funding:** SANOFI PASTEUR S.A.

Primary contact point:

- Juan Vargas, Medical Evidence Generation, Lead, 14 Espace Henri Vallee, Lyon, 69007, France. Juan.vargas@sanofi.com

History of Protocol Versions

| Version | Date | Comments |
| --- | --- | --- |
| 1.0 | 3 July 2023 | Initial version |
| 2.0 | 30 June 2025 | - EU PAS Register number was added - Wrong term ‘total impact’ has been changed to ‘impact’ or ‘overall impact’ - Wrong wordings in the definitions in sections 3b, 3ei, 3dii and 6d have been corrected   Wrong wordings struck through, new wordings with italics |

**Abbreviations**

2aP vaccine Pertussis vaccine containing 2 vaccine antigens

3aP vaccine Pertussis vaccine containing 3 vaccine antigens

aP Acellular pertussis vaccine

AvoHILMO Outpatient visits register (Register of Primary Health Care Visits)

CI Confidence interval

DTaP-IPV-Hib Full-dose pentavalent diphtheria, tetanus, acellular pertussis, poliomyelitis (inactivated) and *Hemophilus influenzae* type B conjugate combination vaccine

DTaP-IPV Full dose tetravalent diphtheria, tetanus, acellular pertussis, and poliomyelitis (inactivated) combination vaccine

dtap Low-dose diphtheria, tetanus, and acellular pertussis combination booster vaccine

DTwP Trivalent diphtheria, tetanus, and whole-cell pertussis combination vaccine

DVV Digital and Population Data Services Agency

Findata The data permit authority for the social and health care sector

FVR FVR – Finnish Vaccine Research

HILMO Hospital discharge register (Care Register for Health Care)

ICD-9 International Statistical Classification of Diseases and Related Health Problems, 9th Revision

ICD-10 International Statistical Classification of Diseases and Related Health Problems, 10th Revision

ICPC-2 International Classification of Primary Care, 2nd edition

NIDR National Infectious Diseases Register

NVP National Vaccination program

NVR National Vaccination Register (Part of AvoHILMO)

PIC Personal identity code

PCR Polymerase chain reaction

RR Relative risk

SAP Statistical Analysis Plan

RWE Real-world evidence

THL Finnish Institute for Health and Welfare

wP Whole cell pertussis vaccine

**Table of Contents**

[1. Introduction 5](#_Toc139278624)

[2. Study objectives 8](#_Toc139278626)

[a. Primary objective 8](#_Toc139278627)

[b. Secondary objectives 8](#_Toc139278628)

[c. Exploratory objectives 9](#_Toc139278629)

[3. Description of the research methods 9](#_Toc139278630)

[a. Study design 9](#_Toc139278631)

[b. The data sources 11](#_Toc139278634)

[c. Study population 13](#_Toc139278635)

[d. Study period and follow-up 14](#_Toc139278641)

[e. Exposures 15](#_Toc139278642)

[f. Outcome definitions and endpoints 16](#_Toc139278643)

[4. Sample size 17](#_Toc139278644)

[5. Data management, quality control and methods in assembling the study data 19](#_Toc139278645)

[6. Methods for data analysis 20](#_Toc139278646)

[a. Effectiveness of preschool vaccination 20](#_Toc139278647)

[b. Impact of preschool vaccination 21](#_Toc139278648)

[c. Descriptive Statistics 21](#_Toc139278649)

[d. Sensitivity analyses 22](#_Toc139278650)

[7. Limitations and strengths of the study design, data sources, and analytic methods 23](#_Toc139278651)

[a. Strengths 23](#_Toc139278652)

[b. Limitations 23](#_Toc139278653)

[8. A description of plans for protecting human subjects 24](#_Toc139278654)

[9. Communication plan 24](#_Toc139278655)

[10. Archiving of protocol 24](#_Toc139278656)

[11. The proposed study tasks, milestones, and timeline 25](#_Toc139278657)

[a. Investigator institute 25](#_Toc139278658)

[b. Study manager 25](#_Toc139278659)

[c. FVR study team, scientific, RWE unit 25](#_Toc139278660)

[d. Stakeholders and collaboration parties 25](#_Toc139278661)

[e. Study coordination and documentation 25](#_Toc139278662)

[f. Study milestones and timelines 26](#_Toc139278663)

[12. References 26](#_Toc139278664)

# Introduction

*Bordetella pertussis* is a Gram-negative, aerobic coccobacillus, which causes a highly contagious disease, pertussis or whooping cough in humans (Mattoo 2005). Before the pertussis vaccine was introduced in the first half of the 20^th^ century, it was one of the ‘deadly scourges of childhood’, a severe disease causing hospitalizations with intensive care treatment and deaths because of breathing difficulties and apnea in young infants. In older children, adolescents and adults, pertussis classically causes repeated paroxysmal fits of forceful coughing for several weeks. Complications of pertussis include pneumonia, otitis media, seizures, and encephalopathy. Similar disease, although of less severity, is sometimes also caused by other *Bordetella species including B. parapertussis*, *B. bronchiseptica* and *B. holmesii* (1).

Before the vaccination era, the incidence of observed pertussis cases was 157/100,000 in US and 230/100,000 in UK, but it was realized that this is a clear underestimation, and the true incidence was considerably higher (2). In addition to endemic baseline disease, pertussis caused epidemics at 3-5 years’ intervals, which is still currently the case. With the widespread introduction of whole-cell pertussis vaccines in the 1940’s and early 1950’s, the rate of reported pertussis fell dramatically and was 0.5/100,00 in US and 1.0/100,00 in UK for the 7-year period between 1976 and 1982 (2).

**Pertussis disease in Finland**

Before pertussis vaccinations started in Finland in 1952, about 10 000–20 000 pertussis cases were reported yearly and 100–400 subjects died. In 1970’s, pertussis was very rare (3,4).

The serological diagnostic methods based on IgA and IgM antibodies were introduced in early 1980’s in Finland. (4). The polymerase chain reaction (PCR) test was introduced nationally in 1993 and its availability throughout the country has been good since late 1990’s. The diagnosis in young children is mostly based on PCR or traditional culture, while serology is more important in older children and adults, and the majority of all cases are based on serology (4, 5). According to the Communicable Diseases Act, pertussis is a monitored disease in Finland, and all laboratories in the country are obliged to send the information of detections into The National Infectious Disease Register (NIDR) since 1995. The current national recommendation on how to clinically diagnose pertussis and when to obtain a sample does not give detailed instructions (6).

It is not known, how much the improved diagnostics contributed to the increase of average observed pertussis cases in 1980’s from less than 500 cases per year to almost 1,000 cases per year in late 1990’s, with large epidemics in 1983, 1990-1991, and 2003-2004 (3,4).

**Vaccination Schedules in Finland**

Since 1970’s, the National vaccination program (NVP) included a combination of diphtheria, tetanus, and two-strain whole-cell pertussis vaccine (wP), produced by the National Public Health Institute (KTL), the predecessor to Finnish Institute for Health and Welfare (THL). It was offered to all children at 3, 4, 5 and 24 months of age in public well-baby clinic network, free of charge (Table A).

In 2005, KTL stopped the vaccine production and the NVP switched from the more reactogenic whole-cell pertussis vaccine to an acellular pertussis vaccine (aP). Already in 2003, a low-dose acellular booster (fourth dose) in combination with low-dose diphtheria and tetanus boosters (dtap) was introduced for children at 6 years of age (7,8). In 2005, vaccination schedule in NVP was changed and expanded. Vaccination schedule of 3, 5 and 12 months, already used in Scandinavian countries, was introduced. The acellular pertussis vaccine was a part of pentavalent combination vaccine with diphtheria, pertussis, tetanus, inactivated polio and *Hemophilus influenzae* type B antigens (DTaP-IPV-Hib). In 2005, the timing of the fourth dose was changed from 6 years to 4 years of age, and the vaccine was always the tetravalent combination with diphtheria, tetanus, and inactivated polio vaccines (DTaP-IPV). DTaP-IPV vaccine was also given to those children who needed a catch-up vaccine at 6 years of age in 2005-2010. In addition, a low-dose dtap vaccine (fifth dose) was introduced for children 14 -15 years of age in 2005. For children who had not received the booster dose at 6 years of age, dtap vaccine (fourth dose) was offered at 11-13 years of age.

**Table A. The National Vaccination Programs in Finland**

| Age | -2002 | 2003-2004 | 2005-2010 | | 2011- |
| --- | --- | --- | --- | --- | --- |
| 3 mo | DTwP | DTwP | DTaP-IPV-Hib | Transition period, with transitional and catch-up doses, scheduled according to how many doses the child had already received in the old vaccination program | DTaP-IPV-Hib |
| 4 mo | DTwP | DTwP |  |  |  |
| 5 mo | DTwP | DTwP | DTaP-IPV-Hib |  | DTaP-IPV-Hib |
| 12 mo |  |  | DTaP-IPV-Hib |  | DTaP-IPV-Hib |
| 14-18 mo |  |  | (DTaP-IPV-Hib) |  |  |
| 20-24 mo | DTwP | DTwP | (DTaP-IPV) |  |  |
| 4 y |  |  | DTaP-IPV |  | DTaP-IPV |
| 6 y |  | dtap | (DTaP-IPV) |  |  |
| 11-13 y |  |  | (dtap^1^) |  |  |
| 14-15 y |  |  | dtap |  | dtap |
| Conscripts |  |  |  | | dtap (2012-) |
| 25y and healthcare staff^2^ | |  |  | | dtap (2018-) |

The vaccinations in parenthesis represent the transitional and catch-up vaccinations not included in the new vaccination program. ^1^For children who had not received the dtap vaccine at 6 years of age.^2^Taking care of infants less than one year of age, with revaccination every 5 years.

At the NVP switch, transitional and catch-up vaccinations were offered at different ages, depending on how many doses the child had received that far (9). In 2005-2008, 2-component vaccines were used. From 2009, mainly 3-component vaccines were used, as tetravalent preschool doses until end of 2010 and as pentavalent infant doses for several years thereafter, whereafter 2-component vaccines were used again. The NVP was further developed, with adult booster doses added to conscripts (2012) and young adults at 25-years of age and health care workers taking care of infants less than one year of age (2018). One of the most important goals of pertussis vaccination is to protect the vulnerable young infants by vaccinating their close contacts (cocoon strategy). Vaccination of pregnant women has never been included in the NVP in Finland.

The proportion of children who have received pertussis vaccine of all children (vaccination coverage) has traditionally been high in Finland. Before establishment of the National Vaccination Register (NVR), vaccination coverage has been monitored by biannual review of vaccination records of well-baby clinics. For children born 1988-1992, records of all children (about 2,000 per birth cohort) in sampled well-baby clinics (32-34) were checked and in children born 1995-2001, records of children randomly selected from the Finnish population system (about 1,000 per birth cohort) were checked. The coverage of the first 3 vaccine doses varied between 97.8 to 99.9 and the coverage of the fourth dose (at 24 months of age) varied between 94.7 to 98.5 (10-13).

NVR was first established in 2009, and since 2011, all public health care providers have comprehensively sent the vaccination records to it ongoingly. Monitored by NVR (14), the coverage of the two first DTaP-IPV-Hib doses has varied from 96 to 99% in children born 2013-2019, and the coverage of the third dose has been 88-95%. The vaccination coverage for DTaP-IPV between 2 to 7 years of age has been

- - in birth cohort 2009: 97% (range 92-98% by county, N=18)
  - in birth cohort 2010: 97% (range 95-98%)
  - in birth cohort 2011: 95% (range 93-97%)
  - in birth cohort 2012: 88% (range 81-94% by county, N=19)
  - in birth cohort 2013: 87% (range 83-92%)
  - in birth cohort 2014; 94% (range 90-97%)

The importance of pertussis vaccination is obvious when examining the pertussis epidemiology in Sweden. The vaccination coverage with wP was high, until the pertussis vaccine was withdrawn 1979 due to concerns about the safety and efficacy (15). During the era of no vaccination, the incidence of pertussis increased significantly, until it rapidly and strongly decreased after the inclusion of an acellular pertussis vaccine into the vaccination program in 1996 (16). Similar re-emergence of pertussis after temporary suspension of vaccination and clear decline after the introduction of an acellular vaccine into the vaccination program occurred in Japan (17).

In a meta-analysis of clinical trials and observational studies, the overall effectiveness of wP vaccines from three manufacturers after complete 3-dose series was 94% (95% Confidence Interval (CI) 88-97%) and the overall efficacy of 3- and 5-component aP after complete 3-dose series was 84% (95% CI 81-87%). The efficacy/effectiveness in reviewed studies varied according to the study design, number and schedule of doses evaluated, duration of follow-up, the method of case ascertainment and case definition (18). Despite relatively good effectiveness, vaccines do not effectively prevent transmission and the effectiveness starts waning a few years after vaccination, so the bacterium continues to circulate and causes disease and epidemics (19, 20).

Actually, the number of pertussis cases has increased in many European countries during the last decade, as reported by the European Centre for Disease Prevention and Control (ECDC), although the awareness of the pertussis burden and changes in diagnostic practices may play a role in this pattern. In Finland, the number pertussis cases have remained relatively stable after the introduction of the new vaccination program in 2005 (21). With introduction of booster vaccinations after the primary infant series in many countries, the disease burden has shifted to older age-groups, (21, 22) which raises the question about the duration of the vaccine protection and need of additional booster vaccinations in adolescents and adults including pregnant women, to ensure herd protection to the newborns and youngest infants most vulnerable to severe disease. The impact of the vaccination alone is not straightforward to measure, since in the presence of disease, the role of natural boosting of immunization is unknown.

**Study Rationale**

In Finland, we have a possibility to address the epidemiological patterns of pertussis and relate it to vaccination and changes in the vaccination programs with secondary use of real-word data from population-based national health registers, some of which have gathered data for decades. The registers are based on legally binding or otherwise established systems for health care providers in the whole country to provide comprehensive information on e.g., laboratory diagnostics, hospitalizations, clinical diagnoses, and recently also on vaccinations. The information is also largely individually linkable using a personal identity code, given to all permanent residents of Finland. The national health insurance, centralized public health care system, good access to care, skilled staff, and the trust of the population form a unique setting for studies.

Although acellular vaccines are widely used there is no direct effectiveness data on 2aP vaccines when used as school-entry boosters, although ecological data coming from various Scandinavian countries showed the vaccine provided an adequate protection at population-level (23).

In this study, we aim to assess the epidemiological patterns of pertussis in children less than 14 years of age in 1995-2019 and to evaluate especially the effects of the preschool booster dose, scheduled at 4 years of age in the current NVP. We use the cohort design to estimate the direct effectiveness of the preschool booster in children with individual information of the vaccination available in the NVR and comprehensively linkable with laboratory-confirmed pertussis, hospitalizations, and clinical diagnoses. This allows a generalizable estimate with reasonably large sample size. To assess the ~~total~~ impact of the preschool vaccination, we use the before-after comparison between the old program without a preschool booster and current program including the booster and examine the effect of the booster in children above the age of 4 years and the indirect effect in younger children. This design is used because for the old program, the information of laboratory-confirmed pertussis by time, age, sex, and area are available with overall estimates of vaccination coverage but no individual vaccination information.

Overall, important information would be gained with the study regarding the performance of 2aP vaccines when used a school-entry boosters in a country using only acellular-vaccines with multiple boosters across life. This information could help finetune vaccination schedules and increase confidence in vaccination.


# Study objectives

## Primary objective

The primary objective of the study is to evaluate the direct effectiveness of a 2-component acellular pertussis (2aP) vaccine administered as a preschool booster at 4 years of age against laboratory-confirmed pertussis during a follow-up of 5 years.

## Secondary objectives

The secondary objectives of the study are

1. to evaluate the effectiveness of a 2aP vaccine administered as a preschool booster against laboratory-confirmed pertussis by time since vaccination (waning of effectiveness)

2. to evaluate the effectiveness of a 2aP vaccine administered as a preschool booster against laboratory-confirmed pertussis temporally related with inpatient hospitalization.

3. to assess the impact of a 2aP vaccine administered as a preschool booster by comparing the pertussis incidence rates between the old vaccination program without preschool booster (1995-2002) and the current vaccination program with preschool booster (2012-2019) in defined time intervals.

4. to evaluate the effectiveness of a 2aP vaccine administered as a preschool booster against laboratory-confirmed pertussis by previous vaccination status

## Exploratory objectives

The exploratory objectives of the study are

1. to describe the patterns of laboratory-confirmed pertussis disease in children under 14 years of age

a. by time, age-group, sex, geographic area, type of the specimen and diagnostic method

b. by inpatient hospitalization

c. by recurrence of the infection

d. by vaccination status (different combinations of 0-4 doses) and by age

2. to describe the median age at each pertussis vaccination and the proportion of fully vaccinated cases (3 primary acellular pertussis vaccine doses and a preschool booster)

3. to describe the cumulative incidence of laboratory-confirmed pertussis by calendar year per age group

4. to describe the indirect impact of the preschool booster in younger age-groups (less than 4 years of age) accounting for changes in the vaccination calendar

5. to evaluate the effectiveness of a 2aP vaccine administered as a preschool booster against laboratory-confirmed pertussis related with inpatient hospitalization with clinical diagnoses compatible with pertussis.

6. to evaluate the epidemiology of pertussis diagnosed clinically in outpatient care or inpatient hospitalizations, and the effectiveness and impact of a 2aP vaccine administered as a preschool booster against clinically diagnosed pertussis.

7. to describe the epidemiology of laboratory-confirmed *B. parapertussis,* if available, and clinically diagnosed diseases caused by it.

# Description of the research methods

## Study design

This is a nationwide population-based register study on pertussis incidence in Finland. We will describe the epidemiology of pertussis and the pertussis vaccination programs in children less than 14 years of age in calendar years 1995-2019. The study will consist of different study designs and methods to determine the various planned objectives:

- we will estimate the direct effectiveness of the preschool pertussis booster vaccine dose scheduled at 4 years of age in children born 2007-September 2015 with a cohort design which will cover the years 2011-2019, see Figure A.
- we will estimate the ~~total~~ impact of the preschool vaccination by before-after comparison of the incidence rates of laboratory-confirmed pertussis between the period of the old vaccination program without a preschool booster vaccination (years 1995-2002) and the current vaccination program with a preschool booster vaccination at 4 years of age (years 2012-2019), see Figure B.
- we will address several exploratory objectives with descriptive methods, i.e., incidences, frequencies, tables, and figures.


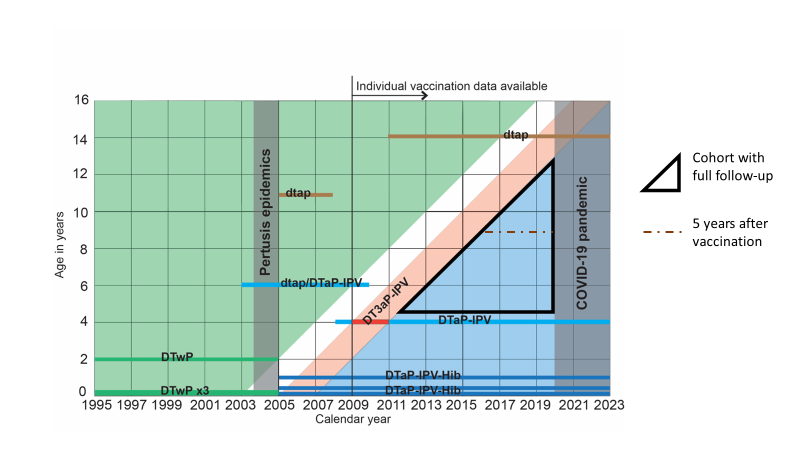


**Figure A.** Cohort design for direct effect of the 2-component acellular pertussis preschool booster dose in 2011-2019. The triangle represents the children followed from the age of 4.25 years to Dec31, 2019 in the cohort analysis. DTwP, combination vaccine containing whole-cell pertussis component. DTap-IPV (+/- Hib), combination vaccine containing acellular pertussis components. 3aP, 3-component acellular pertussis vaccine. dtap, low-dose combination vaccine containing acellular pertussis components.


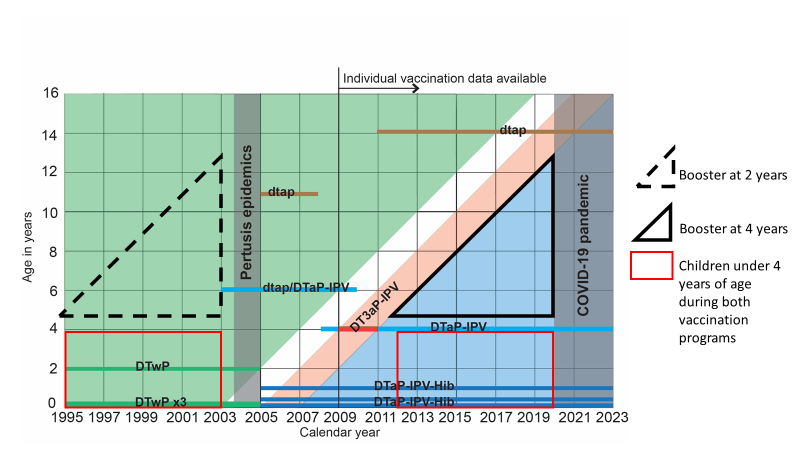


**Figure B.** Before-after comparison for ~~total~~ impact of the current vaccination program including a 2-component acellular pertussis preschool booster dose (2012-2019), as compared to the old vaccination program without a preschool booster dose (1995-2002). The black triangles represent the children from the age of 4.25 years, followed until the end of each period and included in the assessment of the direct (+ indirect) effect of the vaccination program. The red squares represent the children less than 4 years of age during both periods, included in the assessment of the indirect effect of the vaccination program. DTwP, combination vaccine containing whole-cell pertussis component. DTap-IPV (+/- Hib), combination vaccine containing acellular pertussis components. 3aP, 3-component acellular pertussis vaccine. dtap, low-dose combination vaccine containing acellular pertussis components.


## The data sources

The study population for the whole study period will be defined with the published population statistics provided by Statistics Finland, based on the data from the Finnish Population Information System, maintained by the Digital and Population Data Services Agency (DVV).

The data will be extracted from the following nationwide registers:

- **The Digital and Population Data Services Agency (DVV)** maintains a Population Information System, a computerized national register that contains basic information about Finnish citizens and foreign citizens residing in Finland on a permanent or temporary basis. Personal data recorded in the system includes, e.g., the date of birth and death (if applicable), municipality of residence and place of residence/address. The data includes PIC and can be linked with other individual register data.
  - The register will be used
- for defining the population and follow up of children included in the cohort analysis (born 2007-Sep 30, 2015), i.e., dates of birth, immigration to Finland, emigration from Finland and death, if available
- for defining the *foreign background* ~~birth country~~ of the children in the cohort analysis *(birth country of* their parents *and the native language of the children)*
- **The National Vaccination Register (NVR)** is based on vaccination records in the **Register of Primary Health Care Visits (AVOHILMO)**, both maintained by THL. NVR includes all vaccinations administered in the Finnish public health care, which is in charge of delivering the vaccines included in the National Vaccination Program (NVP). The register was first established in 2009, but the data is comprehensive since 2011. Also, vaccinations delivered at private health care are increasingly reported. The data includes PIC and can be linked with other individual register data.
  - The register will be used for collecting individual vaccinations against pertussis of the study children born 2007 and later, from birth until Dec 31, 2019 (e.g., administration date, vaccine, trade name, batch number).
- **T****he National Infectious Disease Register (NIDR),** maintained by THL since 1995 is a population-based and laboratory-based surveillance system. All Finnish clinical microbiology laboratories are obliged to notify all detections of *B. pertussis.* The data includes e.g., date and place of sampling, type of the specimen (e.g., nasopharyngeal, serum), diagnostic method, (e.g., culture, serology, polymerase chain reaction, PCR), birth date or age, sex, and municipality of residence of the child.  *S*ince 2004, the data includes PIC and can be linked with other individual register data.
  - The register will be used
    - - for collecting detections for *B. pertussis* in the study children in 1995-2019
      - for collecting detections for *B. parapertussis* in the study children (if available) in 1995-2019
      - for collecting detections for adenovirus, to be used as negative control outcome in the study children in 1995-2019.
- **The Care Register for Health Care (HILMO),** a hospital discharge register maintained by THL since 1994, containing all inpatient hospitalizations in Finland. Since 1998, the register also contains information on outpatient visits in municipal hospitals. The data includes e.g., the hospital, admission and discharge dates and diagnoses recorded with ICD-9 codes (up to 1996) and ICD-10 codes (thereafter). The data includes PIC (revealing the birth date and sex of the child) and can be linked with other individual register data.
  - The register will be used
    - for collecting the inpatient hospitalizations (admission and discharge dates, diagnoses compatible with pertussis) of all study children in 2004-2019
      - for the exploratory analyses of laboratory-confirmed pertussis by hospitalization in all study children 2004-2019
      - for assessing the effectiveness of vaccination against laboratory-confirmed pertussis temporally related to hospitalization with and without diagnoses compatible with pertussis in 2011-2019
      - to collect data on previous hospitalizations as background information for the cohort study, in 2010-2018
    - for collecting pertussis related ICD-9 codes (033*) and ICD-10 codes (A37*) during the whole study period 1995-2019
      - for assessing the effectiveness and impact of vaccination against clinically diagnosed pertussis and to describe its epidemiology
      - for exploratory analyses of epidemiological trends for all *Bordetella* species.
- **The Register of Primary Health Care Visits (AvoHILMO)** maintained by THL contains data on outpatient primary health care visits in public health care since 2011. Currently, also most private health care providers are covered. The data includes e.g., date, place and time of the visit/contact and reason/diagnoses (recorded with ICD-10 codes or ICPC-2 codes). The data includes PIC and can be linked with other individual register data.
  - The register will be used
    - for collecting ICD-10 codes (A37*), and ICPC-2 codes (R71) for pertussis 2011-2019
      - for assessing the effectiveness of vaccination against clinically diagnosed pertussis
      - for exploratory analyses of epidemiological trends for *B. pertussis* and other *Bordetella* species.
    - for collecting the dates of the first and last visit of the children in the cohort study to primary health care between 3.5 to 13 years of age, and to the well-baby clinic services between 3 to 6 years of age, during calendar years 2011 to 2019, to verify the vital status and residence in Finland and to ensure access to care for sensitivity analysis*.*

**The Social Insurance Institutions (KELA) benefits registers** include data on decisions of reimbursement of health care costs of the individual citizens covered by the national health insurance. The data includes PIC and can be linked with other individual register data.

- - The register will be used for collecting special reimbursement decisions for medicines to treat chronic diseases for defining baseline morbidity (e.g., diabetes, lung diseases, diseases with immunosuppressive treatment) in children included in the cohort study.
- **The Medical Birth Register,** maintained by THL (former KTL) since 1987 with several further reforms includes versatile data on deliveries and births in Finland. The data includes PIC and can be linked with other individual register data.
  - The register will be used
    - for collecting background factors of children born in Finland and included in the cohort study, e.g., gestational age at birth, birth weight, previous live births of the mother (as surrogate for older siblings)
    - for collecting neonatal deaths for defining details of the cohort study population

## Study population

The total population for the study will be all permanent residents less than 14 years of age in Finland 1995-2019 according to the Finnish Population Information System.

### ***Population for the cohort study design***

The population eligible for the cohort analysis will consist of children born from January 2007 through September 2015, recorded by DVV as permanent residents in Finland. The annual birth cohorts range between 55,000 to 61,000 live births (mean 58,885; total 516,000).

Exclusion criterium for the primary analysis (primary objective)

- living in municipalities with inadequate vaccination reporting to NVR for the study years

Eligibility criteria for sub-populations for secondary and exploratory analyses in the cohort study (secondary and exploratory objectives)

- - for exploring the effect of previous vaccinations, only children born 2011-September 2015 will be included, since the information of primary (infant) doses of pertussis vaccination is available.

Eligibility criteria for planned sensitivity analyses include

- - children born 2009-September 2015, with less comprehensive data on primary pertussis vaccinations for years 2009-2010
  - children without detected previous pertussis
  - children born in Finland, with information of background factors available only in the Medical Birth Register.

Also, other eligibility criteria may be defined for sensitivity analyses. These will be refined in a separate Statistical Analysis Plan (SAP).

###

### ***Population for the before-after comparison design***

The population for the before-after analysis will consist of two separate Finnish child populations, recorded by DVV as permanent residents in Finland:

- children born October 1990-1998 followed from the age of 4.25 years until Dec 31, 2002, and children born 1995-2002 followed until age of 3.75 years
- children born October 2007-2015 followed from the age of 4.25 years until Dec 31, 2019, and children born 2012-2019 followed until age of 3.75.

### ***Population for the descriptive analyses***

The population for the descriptive analyses will consist of those children in the total study population, who have the information of interest available in registers.

- E.g., pertussis detections in NIDR are available since 1995. These can be individually linked with hospitalizations from HILMO for all children in 2004-2019, outpatient clinical diagnoses are available in AvoHILMO for all children in 2011-2019 and all pertussis vaccination doses are available in NVR for children born 2011-2019.


## Study period and follow-up

### ***Follow-up period in the cohort study design***

The follow-up period will be from the age of 4.25 years until the first outcome event of interest, any pertussis vaccination after the 2aP preschool booster dose or after 6 years of age, (emigration from Finland and death, if available), or Dec 31, 2019, whichever comes first. The follow-up period will range from 0 to 8.75 years, median 4.375 years per subject assuming constant birth cohorts (estimated total follow-up 2.3 million person-years).

- For the primary objective, the follow-up period will last until the first outcome event of interest, age 9.25, or any other pertussis vaccination after the preschool booster dose or after 6 years of age, (emigration from Finland, death, if available), or Dec 31, 2019, whichever comes first. The follow-up period will range from 0 to 4.75 years, median 3.46 years per subject assuming constant birth cohorts (estimated total follow-up 1.8 million person-years).
- For the secondary objective 2.b.1 (waning of effectiveness after preschool vaccination), the follow-up will be stratified to shorter follow-up periods


### ***Study period for the before-after comparison design***

The before-after comparison will comprise two periods, 1995-2002 and 2012-2019, representing vaccination programs with and without a preschool booster dose, respectively.

The reasons for excluding years 2003-~~2010~~ *2011* from the analyses:

- - years 2003-2008 were excluded because of transitional stage between the two vaccination programs, with catch-up pertussis vaccinations at different ages. Especially, an extra low-dose acellular pertussis booster dose was offered to children at 6 years of age since 2003 or at 11-13 years of age since 2005. In addition, exceptional pertussis epidemics occurred during the years 2003-2004.
  - years 2009-2010 were excluded, because a 3-component acellular pertussis vaccine was used in the NVP, instead of a 2-component vaccine.
  - year 2011 was excluded to make the compared time periods comparable, e.g., equal time, matched by calendar time and age
  - years 2020 and onwards were excluded because of COVID-19 pandemic mitigation measures, strongly affecting the occurrence of respiratory tract infections.

### ***Study periods for the descriptive analyses***

The study periods and child cohorts for the exploratory analyses will depend on the study question, consisting of all children in the total study population, or those with information of the interest available in registers.

- E.g., time, age, sex and area for laboratory-confirmed pertussis and study population are available for all children for the whole study period 1995-2019, hospitalizations linkable to pertussis detections are available for all age groups in 2004-2019, and information of all pertussis vaccination doses are available in 2011-2019 for children born 2011-2019.

## Exposures

### ***Exposures for the cohort analysis***

The exposure in the cohort analyses is the preschool 2aP pertussis vaccination (in combination with diphtheria, tetanus, and inactivated poliomyelitis vaccines, DT2aP-IPV), used as time-dependent variable.

- The child is defined to be vaccinated when more than 14 days have elapsed since the date of an individual record in the NVR of administration and the pertussis vaccine of interest
  - definition for the preschool vaccination is that a 2aP vaccine has been administered at 3 to 6 (less than 7) years of age.
- the definitions for the previous (primary) vaccinations (doses 1-3) are that the first, second and/or third dose of pertussis vaccine have been administered before 3 years of age.
- The child is defined to be unvaccinated *for the vaccine of interest***,** as far as no individual record of a pertussis vaccine of interest exists in the NVR
  - The age limits for different vaccine doses are defined as for vaccinated children.


### ***Exposures for the before-after comparison***

For the comparison of two vaccination programs, the impact of the vaccination program is assessed, consisting of both direct and indirect effects in those vaccinated and indirect effect in those not vaccinated. In this analysis, no individual vaccination data is used. Considering the high vaccination coverage, the children are assumed to have received their age-scheduled doses.

The NVP for pertussis during 1995-2002 included wP vaccine doses (in combination with diphtheria and tetanus vaccines) at 3, 4, 5 and 24 months of age without a preschool pertussis booster vaccination. The NPV for pertussis during 2012-2019 included 2aP vaccine (DT2aP-IPV) doses at 3, 5 and 12 months of age, with a 2aP booster dose at 4 years of age. These NVPs are assumed to be fully followed and considered as exposures in this analysis.

### ***Exposures for the descriptive analyses***

Depending on the study question, the exposures for descriptive analyses are defined as in the cohort analysis, the before-after comparison, or not needed at all.

## Outcome definitions and endpoints

The variables to be used in the analyses are summarized in chapter ‘The data sources’. The detailed technical definitions of variables used for data application from the registers and their transformation into the analysis variables will be included in a Data Management section in a separate SAP.

### ***Outcome case definitions***

- Laboratory-confirmed pertussis is a case of pertussis registered in the NIDR based on detection of *B. pertussis* e.g., by PCR, culture, or serology.
- The pertussis detection is defined to be temporally related with the hospitalization, if the date of the pertussis detection in NIDR is within 14 days before to 7 days after any inpatient hospital admission, according to HILMO.
- The pertussis detection is defined to be clinically related with the hospitalization, if the date of the pertussis detection in NIDR is within 14 days before to 7 days after the inpatient hospital admission, and a clinical diagnosis of pertussis (ICD-10 codes A37 with any further digit 0/1/8/9), acute respiratory tract infection (J00–J22, J40-J47) or severe complication of lower respiratory tract infections (J80–84, J85.1, J86) is recorded during the hospitalization, according to HILMO.
  - Codes for diseases caused by other *Bordetella* species than *B. pertussis* are included considering a probable coding error in the presence of laboratory-confirmed pertussis.
- Clinical diagnosis of pertussis is defined as a record of ICD-9 code 0330A/B, or 0339A/B, ICD-10 code A37.0 or A37.9, or ICPC-2 code (R71) in HILMO or AvoHILMO, irrespective of laboratory confirmation.

### ***Endpoints for the cohort analysis***

The primary endpoint (objective 2.a) is the first occurrence of laboratory-confirmed pertussis in the cohort study population aged 4.25-9.25 years during the cohort follow-up.

The endpoints for secondary (2.b) objectives in the cohort analyses are

- the first occurrence (after the age 4.25 years) of laboratory-confirmed pertussis within follow-up up to 2 years after the age 4.25 years, within 2-year periods thereafter and during the whole follow-up (2.b.1)
  - if needed because of small sample size, the stratified follow up periods may be longer, e.g., 3 years
  - the first occurrence (after the age 4.25 years) of laboratory-confirmed pertussis temporally related with inpatient hospitalization (2.b.2)
  - the first occurrence (after the age 4.25 years) of laboratory-confirmed pertussis (2.b.4) in children born 2011 or later by previous vaccinations
  - No doses vs. full series, i.e., 3 primary doses + preschool booster
  - 1-3 primary doses vs. 1-3 primary doses + preschool booster
  - 1-3 primary doses vs. 3 primary doses + preschool booster
  - 3 primary doses vs. 3 primary doses + preschool booster

The endpoints for exploratory (2.c) objectives in the cohort analysis are

- - the first occurrence (after the age 4.25 years) of laboratory-confirmed pertussis clinically related with inpatient hospitalization (2.c.5)
  - the first occurrence (after the age 4.25 years) of any clinical diagnosis of pertussis (2.c.6)

### ***Endpoints for the before-after comparison design***

The endpoints in this analysis are (in 1995-2002 and 2012-2019, defined populations) (number, incidence, cumulative incidence)

- the records of laboratory-confirmed pertussis, by time, age, sex, and geographic area in NIDR (2.b.3, 2.c.4)
- all clinical diagnoses of pertussis, irrespective of laboratory confirmation and in those with no laboratory confirmation (2.c.6)

### **Endpoints *for the descriptive analyses***

The endpoints for the descriptive analyses will depend on the study question and data availability in the registers (number, incidence, cumulative incidence, when relevant)

- the records of laboratory-confirmed pertussis in 1995-2019, grouped by time, age, sex, geographic area, type of the specimen (e.g., nasopharyngeal, serum) and diagnostic method (e.g., culture, PCR, serology) (2.c.1.a, 2.c.3), or linked as individual information related with
  - hospitalization, in 2004-2019 (2.c.1.b)
    - relationship with hospitalization will be defined as in the cohort analysis
  - recurrence of pertussis infection after recovery from previous one, in 2004-2019 (2.c.1.c)
- the records of individual pertussis vaccinations in NVR from birth until Dec 31, 2019 in children born 2011 (in sensitivity analysis, 2009) or later (e.g., administration date, vaccine, trade name, batch number) (2.c.1.d, 2.c.2)
- the records of laboratory-confirmed pertussis in NIDR by vaccination status according to NVR in 2012-2019, in children born 2011 or later, at one year of age and yearly thereafter, by vaccination status at the time of sampling (2.c.1.d)
  - fully vaccinated (4 doses)
  - partially vaccinated (between 1 and 3 doses)
  - no booster (3 primary doses but no booster)
  - unvaccinated (no doses)
- all clinical diagnoses of pertussis, recorded as ICD-9 codes (033*), ICD-10 codes (A37*), and ICPC-2 code (R71) in HILMO (1995-2019) and in AvoHILMO (2011-2019), irrespective of laboratory confirmation and in those with no laboratory confirmation (2.c.6)
- the records of positive detections for *B. parapertussis* NIDR in 1995-2019 (2.c.7)

# Sample size

The sample size is calculated for the primary endpoint, effectiveness of the preschool pertussis booster to prevent laboratory-confirmed pertussis by cohort design in subjects 4.25 to 9.25 years of age.

The calculation is based on the following assumptions:

- the vaccine effectiveness for the booster dose is 50% (not taking into account any infant doses)
- the vaccination coverage is 93% (source: Vaccination coverage in children, Finland. <https://www.thl.fi/roko/vaccreg/atlas/public/atlas-en.html?show=infantbc>). Thus, ratio of vaccinated to unvaccinated is 0.93:0.07 = 13
- the incidence of laboratory-confirmed pertussis in the NIDR for years 2011-2019 for children 5 to 9 years of age is 12.6 per 100,000 per year.
- The estimated incidence for the unvaccinated will vary according to the assumptions for VE and vaccination coverage, see Table B below.
- the mean follow-up period is 3.46 years for the 516 000 subjects in the cohort
- the total follow-up time is roughly 1.8 million person-years
- power 80/90%
- alfa 5%
- the loss of follow-up will be minimal (deaths, emigrations)

Results are presented in Table B.

**Table B.** The required number of vaccinated and unvaccinated children and the size of the total cohort with varying levels of vaccine effectiveness against laboratory-confirmed pertussis (30-90%) and power (80/90%). Two-sided confidence interval is 5%, median follow-up 3.46 years and the proportion of vaccinated in the total cohort 93%.

| Power (%) | Mean FUP | Vaccination coverage rate (%) | VE (%) | Incidence, unvaccinated (/100 000 person-years) | N  total | N vaccinated | N unvaccinated |
| --- | --- | --- | --- | --- | --- | --- | --- |
| 80 | 3.46 | 93 | 30 | 17.48 | 1717000 | 1596810 | 120190 |
| 80 | 3.46 | 93 | 40 | 20.6 | 767600 | 713868 | 53732 |
| **80** | **3.46** | **93** | **50** | **23.55** | **390772** | **363418** | **27354** |
| 80 | 3.46 | 93 | 60 | 28.51 | 195258 | 181590 | 13668 |
| 80 | 3.46 | 93 | 70 | 36.1 | 104714 | 97384 | 7330 |
| 80 | 3.46 | 93 | 80 | 49.22 | 47050 | 43756 | 3293 |
| 80 | 3.46 | 93 | 90 | 77.3 | 18443 | 17152 | 1291 |
| 90 | 3.46 | 93 | 30 | 17.48 | 2437221 | 2266616 | 170605 |
| 90 | 3.46 | 93 | 40 | 20.6 | 1080658 | 1005012 | 75646 |
| 90 | 3.46 | 93 | 50 | 23.55 | 547619 | 509286 | 38333 |
| 90 | 3.46 | 93 | 60 | 28.51 | 283903 | 264030 | 19873 |
| 90 | 3.46 | 93 | 70 | 36.1 | 140000 | 130200 | 9800 |
| 90 | 3.46 | 93 | 80 | 49.22 | 66056 | 61432 | 4624 |
| 90 | 3.46 | 93 | 90 | 77.3 | 27300 | 25389 | 1911 |

To reach power of 80% and two-sided confidence level of 95% with vaccine effectiveness 50% and 3.46 years of follow-up, 363,418 vaccinated and 27,354 unvaccinated children would be needed. With the estimated total cohort size of 516,000 children, the power is adequate to show statistically significant difference between vaccinated and unvaccinated children with vaccine effectiveness of at least 50%.

Figure C shows the required total number of study subjects with different levels of vaccination coverage.

**
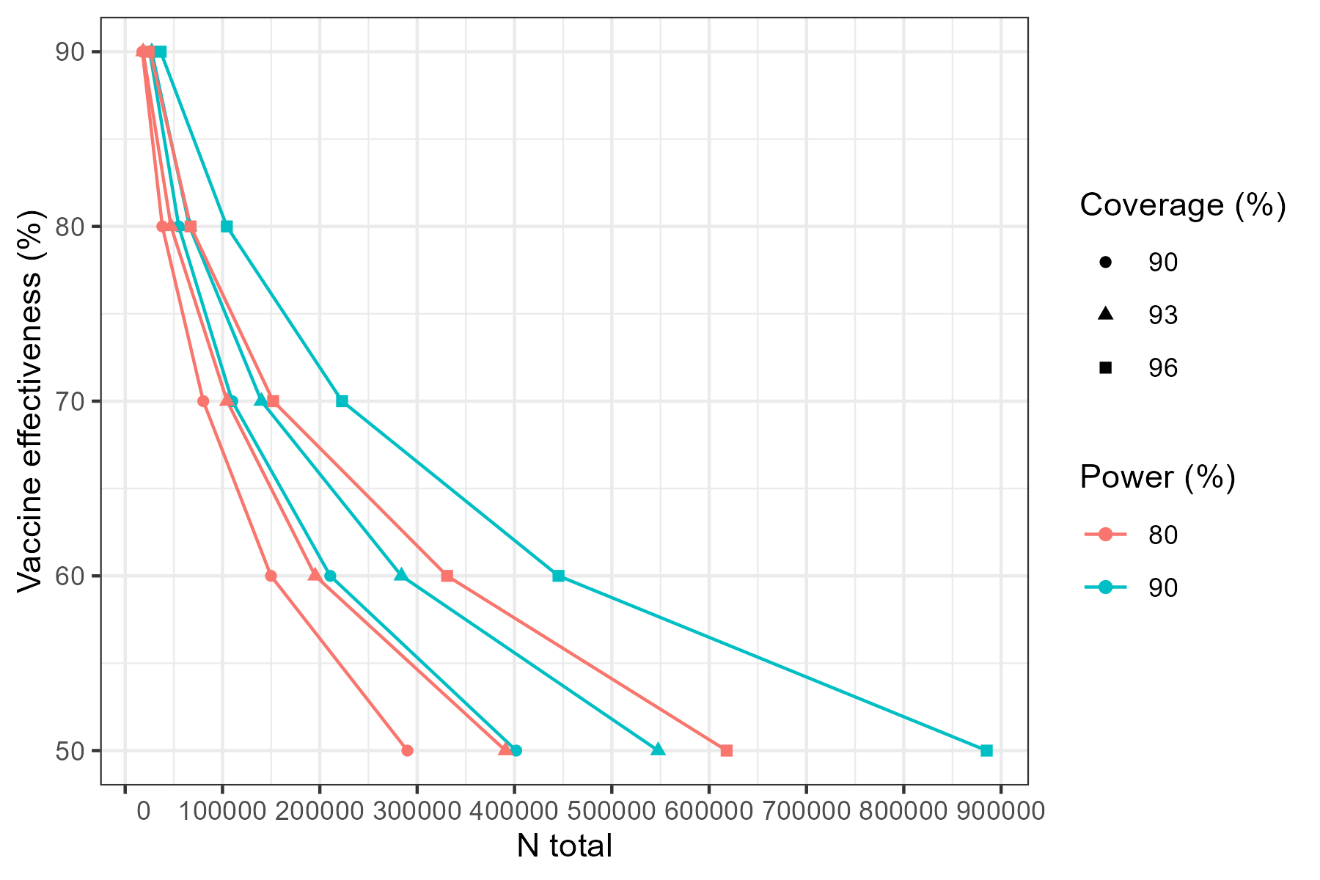
**

**Figure C.** Total sample size with varying levels of vaccine effectiveness against laboratory confirmed pertussis (50-90%), power (80/90%) and the proportion of vaccinated in the total cohort (90-96%). Two-sided confidence interval is 5% and median follow-up 3.46 years. Vertical line at the 516,000 subjects.

# Data management, quality control and methods in assembling the study data

For the individual data access from the registers (see section Data sources), the data will be applied from the Finnish Social and Health Data Permit Authority Findata according to its requirements. Findata extracts the data from controllers of the original registers, pseudonymizes the data and delivers it into a secure operating environment registered and overseen by the National Supervisory Authority for Welfare and Health (Valvira). The data can be processed and analyzed in the secure operating environment by investigators registered as users.

Clear technical definitions of variables to be extracted from the registers must be used for the Findata application, based on descriptions published by the register controllers. The technical definitions will be included in a Data Management section in a separate SAP. The register data sets transferred by the Findata into the secure operating environment will form the source data in this study. Findata is responsible for building the source datasets with the variables from the multiple sources.

The analysis data sets, i.e., variables to be used in the analyses will be derived by the FVR data analyst from these source data according to detailed specifications, described in the SAP. The PICs will not be available, but the pseudonymization codes will allow linking of individual information from different registers.

Statistical programs will be provided by the owner of the secure operating environment. R program will be used for compiling the datasets and performing the analyses. Programming codes will be saved and version-controlled for full audit trail. Other statistical programs (e.g., SPSS) may be used for data exploration.

Both the source data and the analysis data sets will be checked for accuracy (e.g. checking frequencies in subgroups, searching for inconsistencies). For example, if the vaccination coverages of infant doses are locally or temporally lower than expected, the children residing in these municipalities will be exluded from the cohort analysis.

Small frequencies may need to be masked based on data privacy policies. Only aggregate result outputs can be delivered from the secure operating environment and shared to study collaborators not registered as users of the secured operating environment. Findata will check the anonymization of the data before its release.

When all planned analyses have been performed, both the source data sets and the analysis data sets will be transferred to the archive mode of the secure operating environment. The study data and the codes to transform the variables in the original register data into variables to be used in the analyses will be maintained for at least 5 years after final report or first publication of study results.

# Methods for data analysis

A detailed SAP will be finalized before the analyses.

## Effectiveness of preschool vaccination

Effectiveness will be determined by the cohort method.

The incidence of laboratory-confirmed pertussis will be compared between those unvaccinated and vaccinated with the 2aP preschool booster dose. The children will be followed from the age of 4.25 years until the first outcome event, any pertussis vaccination after the preschool vaccination or after 6 years of age, (emigration from Finland, death, if available), or Dec 31, 2019, whichever comes first, (total follow-up of about 2 million person-years).

- As the primary objective, the follow-up will last until the age of 9.25 years. Stratified secondary analyses by time since vaccination will be performed as needed, and as sample size allows.
- Other secondary and exploratory analyses comprise assessing the effectiveness against laboratory-confirmed pertussis temporally related with hospitalization (with and without a clinical diagnosis compatible for pertussis) and against clinically diagnosed pertussis (irrespective of laboratory confirmation and in those without laboratory confirmation), if sample size allows.

In this time-to-event framework, vaccine effectiveness (VE) is defined as 1-relative risk (RR), analyzed with e.g., Cox regression. Only the first event of interest per child will be considered.

The VE estimate will be adjusted for age, sex, area, and calendar time.

Additionally, other background factors will be examined, and the relevant ones used in the analyses, if data availability and sample size allow

- - number of previous pertussis vaccine doses in NVR (available for children born 2011 and later and less comprehensively for children born 2009-2010). Specifically, the following sub-analyses will be considered:
    - No doses vs. full series, i.e., 3 primary doses + preschool booster (absolute effectiveness)
    - 1-3 primary doses vs. 1-3 primary doses + preschool booster
    - 1-3 primary doses vs. 3 primary doses + preschool booster (relative effectiveness)
    - 3 primary doses vs. 3 primary doses + preschool booster

Children who have received more than 3 primary doses will be excluded from these analyses

- - chronic diseases according to the records of special reimbursement decisions for medicines to treat chronic diseases (e.g., diabetes, lung diseases, diseases with immunosuppressive treatment) in KELA
  - the number of previous inpatient hospitalizations (one year before start of the follow-up and yearly thereafter) in HILMO
  - foreign background (birth country and birth country of the parents)

The precision of the VE estimates will be expressed with respective 95% confidence intervals (CI).

## Impact of preschool vaccination

To determine the impact of the vaccination program, incidence rate ratios will be calculated by comparing incidence and cumulative incidence between years without the use of a preschool pertussis booster (1995-2002) with those after the 2aP-preschool booster was introduced (in 2012-2019) in children followed form 4.25 years of age until the end of the respective study period.

Cumulative incidence will be estimated based on the number of reported cases per age group divided by the respective population: Incidence= #Cases_Age_x-yearx_/#Population_Age_x-yearx_

As an exploratory objective, the incidence rates of laboratory-confirmed pertussis in children aged less than 3.75 years will be compared between years 1995-2002 and 2012-2019, if sample size allows. The indirect effect is expected to develop about one year after the 4-year booster vaccination with 2aP vaccine.

The impact will be expressed as 1 - incidence rate ratio (IRR) and estimated with Poisson regression. The follow-up times will be matched for age and season by design.

The precision will be expressed with respective 95% CIs.

## Descriptive Statistics

Demographics by preschool booster vaccination status (including median age at vaccination) will be described.

Number/proportion of cases and incidence of laboratory-confirmed pertussis in the whole Finnish population less than 14 years of age will be presented

- by calendar year, age group, sex, area, season, and diagnostic method for the whole study period 1995-2019
- by hospitalization (2004-2019)
- by recurrence of the infection (after recovery from previous one) (2004-2019).

The patterns of laboratory-confirmed pertussis disease at one year of age and yearly thereafter, in children born 2011 or later will be described for 2012-2019 by vaccination status:

- - - fully vaccinated (4 doses)
    - partially vaccinated (between 1 and 3 doses)
    - no booster (3 primary doses but no booster)
    - unvaccinated (no doses)

The median age at each pertussis vaccination and the proportion of fully vaccinated cases (3 primary acellular pertussis vaccine doses and a preschool booster) will be described.

Pertussis cumulative incidence by calendar year per age and age group will be described in the study population for the whole study period:

- Cumulative incidence will be estimated based on the number of reported cases per age group divided by the respective population: Incidence= #Cases_Age_x-yearx_/#Population_Age_x-yearx_

The epidemiology of laboratory-confirmed and clinically diagnosed infection caused by *B. parapertussis* (if relevant data is available), and as a negative control outcome, laboratory-confirmed adenovirus will be described for the whole study period.

The distribution of clinical diagnoses of pertussis, set on outpatient visits and during hospitalizations will be described for the whole study period.

## Sensitivity analyses

- The cohort analysis will be repeated in children born in Finland by using data from the Medical Birth Register
  - Additional data will comprise the records of gestational age at birth, birth weight, previous live births of the mother (as surrogate for older siblings) in the Medical Birth Register
- The cohort analysis will be repeated with population for which the vital status, residence in Finland and ensured access to care during the follow-up has been verified using
  - the infant deaths from the Medical Birth Register and
  - the dates of the first and last visit to primary health care between 3.5 to 13 years of age, and to the well-baby clinic services between 3 to 6 years of age from AvoHILMO, during calendar years 2010 to 2019.
- The effectiveness of the preschool pertussis vaccine by previous vaccination status will be repeated in all children born in 2009 or later.
- The effectiveness of the preschool pertussis vaccine will be assessed in children without detected laboratory-confirmed pertussis *and without laboratory-confirmed pertussis* with clinically diagnosed pertussis before the follow-up
- The cohort analysis and the before-after comparison will be repeated using adenovirus detected in the NIDR as a negative control outcome, to validate the methods
- The cohort analysis will be repeated in children born March 1, 2007 to Sept 30, 2015, with follow-up starting at 3.75 years of age

# Limitations and strengths of the study design, data sources, and analytic methods

## Strengths

The data used in this study is derived from Finnish national registers, which are nationwide, complete, real-time, linkable, and affordable. They are based on the health care system, which offers a national health insurance and public health care with easy access to all citizens. The personnel are skilled and the citizen’s trust to health care professionals is high. Some of the registers are defined by law and transfer of data is obligatory. Others have been established for decades ago, developed and validated continuously. The new ‘Secondary use law’ automates the data confidentiality and security and guarantees certified and validated environment for data processing.

Because the registers are based on real-time population data, loss to follow-up principally occurs only if the person emigrated out of Finland. Because the registers cover the whole country and represent real-word data, the generalizability is high. Also, the sample size is relatively high. The analysis methods are established and widely used.

## Limitations

Like all observational studies, the results are affected by confounding and effect modifiers that cannot be controlled in a reliable way, unlike in randomized, controlled, and blinded clinical trials. The inclusion of the whole population levels off this caveat but there may be differences between the vaccinated and unvaccinated populations.

The registers are based on recording events. Lack of an event is interpreted as no event and potential missing data cannot be recognized, which ultimately could lead to a lower power of the study. Missing vaccination record could ultimately lead to a lower vaccine effectiveness. However, misclassification in exposure is unlikely, since the vast majority of childhood vaccinations are historically and continuously administered in the public well-baby clinic network, and the vaccination coverage based on the NVR is high, so unrecognized vaccinations are likely to be few. Pertussis cases may remain un-diagnosed and the outcomes underestimated. This is expected to be non-differential because the underestimation of the outcome is likely to be symmetric in relation to the exposure. As pertussis is by law a monitored communicable disease, all laboratories must report detections in the NIDR, and all hospitals report visits into HILMO. However, differences in the diagnostic practices are possible between the two study periods in the before-after comparison. For example, the use of the sensitive PCR diagnostics has been more frequent in the last study period. This will be estimated with the negative control outcome, for which adenovirus was chosen because there is no vaccine used against it, data with numbers comparable to pertussis is available in NIDR, and the incidence has been relatively stable over the study years. The diagnostic practices must be considered carefully when interpreting the results.

Another limitation of the study is the availability of the full vaccination schedule. A vaccination register is fully in place since 2011 as previously mentioned although data are partly available since 2009. Nonetheless, based on administrative data reported by WHO and also directly by the Finnish government, there were very high vaccination coverage estimates for at least one dose. Therefore, the determination of effectiveness will be mostly based on pertussis-primed children. To further clarify the effectiveness compared to fully unvaccinated, an analysis is planned only in children for whom the full vaccination status is known, which would allow to determine the real absolute effectiveness of infants fully unvaccinated vs those that have received the full primary schedule and the booster, and also a measure of the effectiveness of those who received at least one dose as infants but no preschool booster compared to those who received at least one dose as infants and the preschool booster dose.

# A description of plans for protecting human subjects

The register data permit will be obtained by FVR from the Finnish Social and Health Data Permit Authority Findata according to its regulations and according to the Finnish ‘Act on the Secondary Use of Social and Health Data’ (Laki sosiaali- ja terveystietojen toissijaisesta käytöstä, 552/2019)’, which regulates the processing of personal data collected during the provision of social and health care as well as personal data collected for the purpose of steering, supervision, researching and collecting statistics within the social and health care sector.

After the data permission, Findata will transfer individual level data according to the protocol and the data application into a certified secure operating environment. The National Supervisory Authority for Welfare and Health (Valvira) verifies, registers, and oversees these secure operating environments and is responsible for ensuring that the environments satisfy the applicable information security and data protection requirements.

Only aggregated data and results (tables and figures) can be obtained outside the secure operating environment, and only after anonymity check by the Findata.

Because the study does not involve intervening in the physical or mental integrity of a person, it does not fall in the scope of medical research. Therefore, no review of an Independent Ethics Committee nor an informed consent of the subject is needed. A Data privacy notice will be made publicly available.

# Communication plan

The study will be registered into the European Union electronic Register of Post-Authorisation Studies (EU PAS Register) or similar.

The results will be published in a peer-reviewed journal, as agreed in the study contract between the investigator organization and the funding party. Authorship will follow guidelines established by the International Committee of Medical Journal Editors (<http://www.icmje.org/>).

Because this is a study based on secondary use of data from existing registers, the results will not be communicated to the Ethics Committee or a regulatory authority.

# Archiving of protocol

The protocol and other relevant documents of the study will be archived in the electronic archives of FVR for for at least 5 years after final report or first publication of study results.

# The proposed study tasks, milestones, and timeline

## Investigator institute

FVR – Suomen rokotetutkimus Oy/FVR-Finnish Vaccine Research Ltd (former legal name: Finnish Vaccine Research Center Finvac Ltd).

The management of FVR is based on its Quality and Risk Management System (QRMS). It is built through Management System for the daily processes and procedures to ensure consistent high-quality. QRMS is documented, developed, and audited in a planned manner.

## Study manager

A single point of contact for any study related matters

Ritva Syrjänen, MD, PhD, Senior Researcher

FVR – Finnish Vaccine Research

RWE-unit

Finn-Medi I | Biokatu 6 | 33520 Tampere | Finland

+358 40 505 5765

[ritva.syrjanen@fvr.fi](mailto:ritva.syrjanen@fvr.fi)

## FVR study team, scientific, RWE unit

Ritva Syrjänen, Senior researcher, study manager, scientific evaluation

Arto Palmu, Principal investigator, chief research officer, scientific evaluation

Hanna Rinta-Kokko, Senior data analyst, scientific evaluation, statistical analyses

Kimmo Niinimäki, Data analyst, scientific evaluation, statistical analyses

Heta Nieminen, Senior researcher, scientific evaluation

Elina Isosaari, Clinical team leader

Maila Kyrölä, Study nurse, Findata contact person

## Stakeholders and collaboration parties

- Findata - the Finnish social and health data permit authority
- Controllers of the original health registers
- The provider of the certified secure operating environment

## Study coordination and documentation

The FVR administration and legal units will manage the study agreements and financial documentation.

The study manager will be responsible for coordination and documentation of the study project, including

- coordination of the protocol finalization
- coordination of the register permissions and data collection
- coordination of the statistical analyses
- the FVR study team organization and education, including study team changes
- the timeline tracking of the study, with an electronic project planner used in FVR
- communication plan
- risk management plan
- study documentation and archiving of the documents
- coordination with other potential stakeholders/third parties
- coordination of any other relevant ad hoc activities for the project.

## Study milestones and timelines

The register data will be applied from Findata during early Q3/2023. Depending on data availability, i.e., Findata processes and the controllers of the original registers, the data is expected to be available early 2024. The first analyses will be available for review by September 2024. The final study report is expected by the first quarter of 2025.

# References

1. Mattoo S and Cherry JD. Molecular pathogenesis, epidemiology, and clinical manifestations of respiratory infections due to Bordetella pertussis and other Bordetella subspecies. Clin Microbiol Rev. 2005 Apr;18(2):326-82.
2. Cherry JD. The epidemiology of pertussis and pertussis immunization in the United Kingdom and the United States: a comparative study. Curr Probl Pediatr. 1984 Feb;14(2):1-78.
3. Huovila R: Hinkuyskä ja sen esiintyminen Suomessa vuosina 1920–1978. Dissertation. Helsinki university 1981. In Finnish
4. Mertsola J. Hinkuyskän torjunta Suomessa 2017-2021. 2017. Working paper 10/2017, The Finnish Institute for Health and Welfare (THL). <https://urn.fi/URN:ISBN:978-952-302-834-0>. In Finnish.
5. Hulkko T, Lyytikäinen O, Kuusi M et al. Tartuntataudit Suomessa 1995-2009. Report 17/2010, The Finnish Institute for Health and Welfare (THL). <https://urn.fi/URN:NBN:fi-fe201205085420>. In Finnish
6. Lower respiratory tract infections (children). Current Care Guidelines. Working group set up by the Finnish Medical Society Duodecim and the Finnish Cardiac Society. Helsinki: The Finnish Medical Society Duodecim, 2023 (referred June 7, 2023). Available online at: www.kaypahoito.fi
7. Nohynek H, Pekkanen E, Hulkko T, et al. Rokottajan käsikirja 2003. Guideline of the National Public Health Institute (KTL), Duodecim 2003. In Finnish.
8. THL website ‘Milloin eri rokotukset ovat alkanet Suomessa’ (When different vaccines have been introduced in Finland). April 4, 2022. Accessed May 31, 2023. <https://thl.fi/fi/web/infektiotaudit-ja-rokotukset/tietoa-rokotuksista/kansallinen-rokotusohjelma/milloin-eri-rokotukset-ovat-alkaneet-suomessa->. In Finnish.
9. Nohynek H, Hulkko T, Rapola S, et al. Rokottajan käsikirja 2005. Guideline of the National Public Health Institute (KTL), Duodecim 2005. In Finnish.
10. Heinäsmäki T, Koskenniemi E, Haapakoski J, et al. Rokotusohjelman kattavuus Suomessa: vuonna 1995 syntyneiden lasten kahden ensimmäisen ikävuoden yleisen rokotusohjelman toteutuminen. Suom. Lääkäril. 2000; 55:1719-21. In Finnish.
11. Joensuu J, Koskenniemi E, Hulkko T, et al. Pikkulasten rokotusohjelman toteutuminen Suomessa. Suom. Lääkäril. 2002; 57:2470-10. In Finnish.
12. Joensuu J, Koskenniemi E, Hulkko T, et al. Pikkulasten rokotusohjelman toteutuu edelleen hyvin. Suom. Lääkäril. 2005; 60:3359-62. In Finnish.
13. Leino T, Koskenniemi E, Saranpää P-R, et al. Rokotuskattavuus edelleen huippuluokkaa. Suom. Lääkäril. 2007; 62: 739-43. In Finnish.
14. THL website, interactive map ‘Rokotuskattavuus’ (Vaccination coverage). Accessed Oct 27, 2022. <https://www.thl.fi/roko/vaccreg/atlas/public/atlas.html?show=infantbc>. In Finnish.
15. Romanus V, Jonsell R and Bergquist S-O. Pertussis in Sweden after the cessation of general immunisation in 1979. Pediatr Infect Dis J. 1987;6:364-71.
16. Olin P, Gustafsson L, Barreto L, et al. Declining pertussis incidence in Sweden following the introduction of acellular pertussis vaccine. Vaccine. 2003 May 16;21(17-18):2015-21.
17. Aoyama T. Acellular pertussis vaccines developed in Japan and their application for disease control. J Infect Dis. 1996 Nov;174 Suppl 3:S264-9.
18. Fulton TR, Phadke VK, Orenstein WA, et al. Protective Effect of Contemporary Pertussis Vaccines: A Systematic Review and Meta-analysis. Clin Infect Dis. 2016 May 1;62(9):1100-1110.
19. Bolotin S, Harvill ET and Crowcroft NS. What to do about pertussis vaccines? Linking what we know about pertussis vaccine effectiveness, immunology and disease transmission to create a better vaccine. Pathog Dis. 2015 Nov;73(8):ftv057.
20. Paradowska-Stankiewicz I, Rumik A, Bogusz J, Zbrzeźniak J, Rastawicki W, Śmietańska K, Vargas-Zambrano JC, Macina D. Duration of protection against Bordetella pertussis infection elicited by whole-cell and acellular vaccine priming in Polish children and adolescents. Vaccine. 2021 Oct 1;39(41):6067-6073.
21. European Centre for Disease Prevention and Control (ECDC).The Surveillance Atlas for Infectious Diseases (pertussis), <https://atlas.ecdc.europa.eu/public/index.aspx?Dataset=27&HealthTopic=38>. Accessed May 23, 2023
22. Zepp F, Heininger U, Mertsola J, et al. Rationale for pertussis booster vaccination throughout life in Europe. Lancet Infect Dis. 2011 Jul;11(7):557-70.
23. Huoi C, Vargas-Zambrano J, Macina D, Vidor E. A combined DTaP-IPV vaccine (Tetraxim®/Tetravac®) used as school-entry booster: a review of more than 20 years of clinical and post-marketing experience. Expert Rev Vaccines. 2022 Sep;21(9):1215-1231.
