# Supplementary material for: Effectiveness and impact of the 2-component acellular pertussis vaccine as a preschool booster in Finland – A register-based study
Source: PLOS Glob Public Health. 2026 Jul 29;6(7):e0006800. doi: 10.1371/journal.pgph.0006800 (PMC13419238; doi:10.1371/journal.pgph.0006800)
Supplement: S1 Appendix — (DOCX) [file pgph.0006800.s002.docx]

# S1 Appendix: Chronic diseases according to the Social Insurance Institutions (KELA) benefits register

13.12.2024, Jussi Halme and Ritva Syrjänen

Children are considered to have a chronic condition, if they have a decision of reimbursement of health care costs based on EITHER a reimbursement code (medicines/diseases) OR the ICD-10 diagnosis (diseases). More information: <https://www.kela.fi/yhteistyokumppanit-laakekorvaukset-laakkeiden-korvausoikeudet> (in Finnish).

1. **Asthma, COPD and other chronic lung diseases**

Reimbursement codes: 203, 304, 344

ICD-10 diagnoses: J41-J47, Z90.2, E84.0

**2. Immunodeficiency**

Reimbursement codes: 120, 127, 129, 134, 157, 202, 281, 293, 303, 313, 326, 340, 3006, 122

ICD-10 diagnoses: D61, D70, D80-84, T86, Z94

**3. Diabetes mellitus**

Reimbursement codes: 103, 177, 215

ICD-10 diagnoses: E10-14

**4. Poor nutrition and premature birth**

Reimbursement codes: 504, 601, 602, 107

ICD-10 diagnoses: E40-E46, E55, F50, P07, D51.0, D51.3, D51.9, D52.0, D53

**5. Cancer**

Reimbursement codes: 128, 130, 150, 117, 175, 180, 189, 316, 324

ICD-10 diagnoses: C00-97, D05.1, (except C44)

**6. Severe renal disease**

Reimbursement codes: 137, 138, 190, 196, 306, 320, 321

ICD-10 diagnoses: I12-13, I15.1, N00-05, N07, N08, N11, N14, N18, N19, N25.0, Z49

**7. Inflammatory bowel diseases**

Reimbursement code: 208

ICD-10 diagnoses: K50-51

**8. Adrenal insufficiency**

Reimbursement code: 105

ICD-10 diagnoses: E25.0, E27, E31.0, E89.6

**9. Other Endocrinologic condition**

Reimbursement codes: 101, 104, 106, 124, 133, 503

ICD-10 diagnoses: E23.0, E89.3

**10. Severe heart disease**

Reimbursement code: 201

ICD-10 diagnoses: I11-13, I15, I20-28, I41-43, I50, P29

**11. Neurological conditions**

Reimbursement codes: 108, 109,

ICD-10 diagnoses: G20-26, G70-72, G80-83, G47.3, I60-69, G35, G12

**12. Severe mental disorders**

Reimbursement code: 112

ICD-10 diagnoses: F20-29

**13. Down syndrome and severe disability**

ICD-10 diagnoses: Q90, F73
