# Supplementary material for: Effectiveness and impact of the 2-component acellular pertussis vaccine as a preschool booster in Finland – A register-based study
Source: PLOS Glob Public Health. 2026 Jul 29;6(7):e0006800. doi: 10.1371/journal.pgph.0006800 (PMC13419238; doi:10.1371/journal.pgph.0006800)
Supplement: S2 Appendix — (DOCX) [file pgph.0006800.s003.docx]

**S2 Appendix: Results from other exploratory and sensitivity analyses**

Epidemiological time trends of pertussis in our study, depicted in Table A, show that cases were distributed evenly throughout the seasons. Diagnostics were mostly conducted by serology (antibody tests): 84% during 1995−2002, 69% during 2003−2010 and 74% during 2011−2019. Recurrent cases were very rare. Only 4 laboratory-confirmed recurrent cases and 11−12 recurrent cases by clinical diagnosis were found. Of all laboratory-confirmed cases, 11% were related to hospitalization during 2003−2010 and 18% during 2011-2019. However, in the population for our primary cohort analysis (children 4.25-9.25 years of age) hospitalizations with pertussis were very rare, and only 4 such cases were observed. Register of Primary Health Care Visits (AvoHilmo) was launched in 2011, which is why there are more overall clinical diagnoses found during this period.

| **Table A. Time trends of pertussis among all study children aged <14 years in 1995–2019** | | | | | |  |  |  |  |
| --- | --- | --- | --- | --- | --- | --- | --- | --- | --- |
| **Characteristic** | **Laboratory-confirmed pertussis, N** | | | | |  | **Clinically diagnosed pertussis, N** | | |
|  | **Total study population** | | |  | **Primary analysis cohort** |  | **Total study population** | | |
|  | **1995–2002** | **2003–2010** | **2011–2019** |  | **2011–2019** |  | **1995–2002** | **2003–2010** | **2011–2019** |
| **Total** | 2 905 | 2 785 | 1 290 |  | 225 |  | 658 | 659 | 928 |
| **Age group** |  |  |  |  |  |  |  |  |  |
| 0–3 | 531 | 586 | 368 |  | – |  | 432 | 453 | 514 |
| 4–8 | 1 031 | 925 | 371 |  | 213 |  | 118 | 112 | 206 |
| 9–13 | 1 343 | 1 274 | 551 |  | 12 |  | 108 | 94 | 208 |
| **Sex** |  |  |  |  |  |  |  |  |  |
| Female | 1 618 | 1 528 | 653 |  | 111 |  | 347 | 338 | 465 |
| Male | 1 276 | 1 249 | 637 |  | 114 |  | 311 | 321 | 463 |
| Missing | 11 | 8 | 0 |  | – |  | – | – | – |
| **Region** |  |  |  |  |  |  |  |  |  |
| Eastern Finland | 547 | 477 | 175 |  | 26 |  | 96 | 141 | 157 |
| Inland Finland | 447 | 442 | 223 |  | 48 |  | 109 | 75 | 119 |
| Northern Finland | 406 | 325 | 114 |  | 12 |  | 122 | 121 | 132 |
| Southern Finland | 1 054 | 1 004 | 518 |  | 102 |  | 244–245 | 206 | 359 |
| Western Finland | 451 | 537 | 260 |  | 37 |  | 85 | 116 | 161 |
| Missing | – | – | – |  | – |  | 1–2 | 0 | 0 |
| **Season** |  |  |  |  |  |  |  |  |  |
| Autumn | 844 | 704 | 311 |  | 53 |  | 204 | 187 | 285 |
| Spring | 558 | 653 | 313 |  | 55 |  | 120 | 131 | 182 |
| Summer | 674 | 632 | 285 |  | 59 |  | 170 | 143 | 187 |
| Winter | 829 | 796 | 381 |  | 58 |  | 164 | 198 | 274 |
| **Specimen** |  |  |  |  |  |  |  |  |  |
| Pharyngeal or nasopharyngeal | 177 | 376 | 158 |  | 18 |  | – | – | – |
| Pharyngeal or nasopharyngeal and other | 14 | 18 | 6 |  | 0 |  | – | – | – |
| Pharyngeal or nasopharyngeal and serum or plasm | 4 | 8 | 1–2 |  | 0 |  | – | – | – |
| Serum or plasm | 2 426 | 1 933 | 961 |  | 196 |  | – | – | – |
| Serum or plasm and blood and other | 4 | 6 | 8 |  | 1–2 |  | – | – | – |
| Other specimen | 222 | 409 | 130–131 |  | 7–9 |  | – | – | – |
| Missing | 58 | 35 | 25 |  | 1–2 |  | – | – | – |
| **Diagnostic method** |  |  |  |  |  |  |  |  |  |
| Ag-detection | 0 | 0 | 0 |  | 0 |  | – | – | – |
| Serology (antibodies) | 2 433 | 1 932 | 961 |  | 196–197 |  | – | – | – |
| Culture | 84 | 86 | 42 |  | 5 |  | – | – | – |
| Culture and PCR or other NAT identification | 31 | 48 | 30 |  | 3 |  | – | – | – |
| PCR or other NAT identification | 339 | 705 | 245 |  | 19 |  | – | – | – |
| Other method | 15 | 14 | 12 |  | 1–2 |  | – | – | – |
| Missing | 3 | 0 | 0 |  | 0 |  | – | – | – |
| **Recurrent** |  |  |  |  |  |  |  |  |  |
| No | 0 | 2 058 | 1 286 |  | 223–224 |  | 651 | 656 | 926–927 |
| Yes | 0 | 0 | 4 |  | 1–2 |  | 7 | 3 | 1–2 |
| Missing | 2 905 | 727 | 0 |  | 0 |  | 0 | 0 | 0 |
| **Temporally related with inpatient hospitalization** | |  |  |  |  |  |  |  |  |
| No | – | 2 504 | 1 097 |  | 221 |  | – | – | – |
| Yes | – | 281 | 193 |  | 4 |  | – | – | – |
| **Temporally related with inpatient hospitalization with a compatible clinical diagnosis** | | | |  |  |  |  |  |  |
| No | – | 2 609 | 1 142 |  | 223–224 |  | – | – | – |
| Yes | – | 176 | 148 |  | 1–2 |  | – | – | – |
| **Diagnosed in hospital setting only** |  |  |  |  |  |  |  |  |  |
| No | – | – | – |  | – |  | 0 | 0 | 395 |
| Yes | – | – | – |  | – |  | 658 | 659 | 533 |
| **Diagnosed in primary care setting only** |  |  |  |  |  |  |  |  |  |
| No | – | – | – |  | – |  | 658 | 659 | 560 |
| Yes | – | – | – |  | – |  | 0 | 0 | 368 |
| **Diagnosed during inpatient hospitalization** |  |  |  |  |  |  |  |  |  |
| No | – | – | – |  | – |  | 268 | 307 | 695 |
| Yes | – | – | – |  | – |  | 390 | 352 | 233 |
| Characteristics assessed at the time of pertussis case occurrence. Frequencies of 1 or 2 are masked following data privacy policies. | | | | | | | | | |
| Data sources: National Infectious Disease Register, Care Register for Health Care | | | | | | | | | |

No large differences were found in demographics between cases and non-cases in the cohort design. Northern Finland was somewhat underrepresented in pertussis cases. (Table B).

**Table B. Demographics and age at laboratory-confirmed pertussis for all study children by design in 1995–2019**

| Characteristics | Total study population (1995–2019) | Before-after design | | Cohort design (2011–2019), primary objective | | |
| --- | --- | --- | --- | --- | --- | --- |
|  |  | Reference (1995–2002)  With laboratory-confirmed pertussis | Target (2012–2019)  With laboratory-confirmed pertussis |  | | |
|  | With laboratory-confirmed pertussis |  |  | With laboratory-confirmed pertussis | Without laboratory-confirmed pertussis | All children |
| Number of children (N) | 6 980 | 871 | 289 | 225 | 466 015 | 466 240 |
| Sex (n, %) |  |  |  |  |  |  |
| Female | 3 799 (54.4) | 491 (56.4) | 145 (50.2) | 111 (49.3) | 227 546 (48.8) | 227 657 (48.8) |
| Male | 3 162 (45.3) | 378–379 (43.4–43.5) | 144 (49.8) | 114 (50.7) | 238 469 (51.2) | 238 583 (51.2) |
| Missing | 19 (0.3) | 1–2 (0.1–0.2) | 0 (0.0) | 0 (0.0) | 0 (0.0) | 0 (0.00) |
|  |  |  |  |  |  |  |
| Region of residence (n, %) |  |  |  |  |  |  |
| Southern Finland | 2 576 (36.9) | 296 (34.0) | 128 (44.3) | 101 (44.9) | 186 879 (40.1) | 186 980 (40.1) |
| Western Finland &  Åland | 1 248 (17.9) | 140 (16.1) | 47 (16.3) | 36 (16.0) | 74 644 (16.0) | 74 680 (16.0) |
| Inland Finland | 1 112 (15.9) | 136 (15.6) | 66 (22.8) | 49 (21.8) | 70 184 (15.1) | 70 233 (15.1) |
| Eastern Finland | 1 199 (17.2) | 192 (22.0) | 23 (8.0) | 26 (11.6) | 65 492 (14.1) | 65 518 (14.1) |
| Northern Finland | 845 (12.1) | 107 (12.3) | 25 (8.7) | 13 (5.8) | 68 816 (14.8) | 68 829 (14.8) |
| Missing | 0 (0.0) | 0 (0.0) | 0 (0.0) | 0 (0.0) | 0 (0.0) | 0 (0.0) |
|  |  |  |  |  |  |  |
| Age at laboratory-confirmed pertussis (years) | |  |  |  |  |  |
| Mean | 7.3 | 6.9 | 6.5 | 5.9 | – | 5.9 |
| Median | 8.0 | 7.0 | 6.0 | 6.0 | – | 6.0 |
| Range | 0.0–13.0 | 4.0-x | 4.0–11.0 | 4.0–9.0 | – | 4.0–9.0 |
| Standard deviation | 4.2 | 1.9 | 2.1 | 1.6 | – | 1.6 |
| Interquartile range | 4.0–11.0 | 5.0–8.0 | 5.0–8.0 | 4.0–7.0 | – | 4.0–7.0 |
| Assessed at the start of follow-up in the cohort designs and at the time of laboratory-confirmed pertussis in others. Frequencies of 1 or 2 are masked following the data privacy policy of Findata. Data sources: Digital and Population Data Services Agency, National Infectious Disease Register | | | | | | |

Primary and booster vaccinations were mostly administered according to the NIP schedule (Table C). The vaccination coverage was high: 92% of children received the complete primary series, and 93% were booster vaccinated (irrespective of the previous doses) among the children eligible for all doses during the study period. Table C is also discussed in the manuscript text.

| **Table C. Age and proportion of children born ≥2011 at each vaccination dose in 2011–2019** | | | | | |
| --- | --- | --- | --- | --- | --- |
|  |  |  |  |  |  |
|  | First primary dose | Second primary dose | Third primary dose | Preschool booster (any number (0–3) of primary doses and booster) | Preschool booster with full primary series (exactly 3 primary doses and booster) |
| Age (years) at vaccination |  |  |  |  |  |
| Mean | 0.27 | 0.46 | 1.04 | 4.14 | 4.13 |
| Median | 0.25 | 0.43 | 1.01 | 4.06 | 4.06 |
| Standard deviation | 0.10 | 0.13 | 0.11 | 0.26 | 0.25 |
| Interquartile range | 0.25–0.27 | 0.42–0.44 | 1.00–1.04 | 4.01–4.16 | 4.01–4.16 |
| Proportion (n, %) of pertussis cases with the dose | | | | | |
| All cases (N = 86) | 79 (91.7%) | 79 (91.9%) | 76 (88.4%) | 71 (82.6%) | 69–70 (80.8%) |
| Proportion (n, %) of children with the dose, born before 1 October 2015 (eligible for all doses) | | | | | |
| All children (N = 249 152) | 244 973 (98.3%) | 242 584 (97.4%) | 230 400 (92.5%) | 232 422 (93.3%) | 217 557 (87.3%) |
| Vaccination schedule for pertussis doses in the Finnish vaccination program: 3, 5, 12, and 48 months (0.25, 0.42, 1, and 4 years). Frequencies of 1 or 2 are masked following the data privacy policy of Findata. Data sources: Digital and Population Data Services Agency, National Vaccination Register | | | | | |
|  | | | | | |

Results from analysis by previous vaccinations (Table D) are discussed in the manuscript. Median follow-up in all analyses was 2.4 years and maximum 4.8 years. Sensitivity analysis extending the study period to start already from Jan 1, 2009 (Table E) produced very similar results, suggesting that vaccination records from the years 2009 and 2010 are also reliable.

| **Table D. Effectiveness of 2aP preschool booster against laboratory-confirmed pertussis by previous vaccination status among children over 4.25 years old and born January 1, 2011–September 30, 2015, in 2011–2019 — Cohort study** | | | | | | | | | | | | |
| --- | --- | --- | --- | --- | --- | --- | --- | --- | --- | --- | --- | --- |
|  | |  |  |  |  |  |  |  |  |  |  | |
| Outcome | | No primary doses and no 2aP preschool booster (reference) | | |  | 3 primary doses with 2aP preschool booster | | |  | VE (95% Cl) | | |
|  |  | N | Follow-up (years) | Cases, n (incidence per 100 000 person-years) |  | N | Follow-up (years) | Cases, n (incidence per 100 000 person-years) |  | Unadjusted | Adjusted | |
| Laboratory-confirmed pertussis | | 2 763 | 5 424 | 7 (129.1) |  | 217 448 | 513 904 | 69 (13.4) |  | 89 (76 to 95) | 88 (74 to 95) | |
| <1 year since vaccination | | – | – | – |  | 217 439 | 168 952 | 37 (21.9) |  | 79 (39 to 93) | 79 (41 to 92) | |
| ≥1 year since vaccination | | – | – | – |  | 178 595 | 344 951 | 32 (9.3) |  | 94 (85 to 97) | 93 (83 to 97) | |
|  | | 3 primary doses Without 2aP preschool booster (reference) | | |  | 3 primary doses with 2aP preschool booster | | |  |  | | |
| Laboratory-confirmed pertussis | | 37 615 | 35 597 | 7 (19.7) |  | 217 448 | 513 904 | 69 (13.4) |  | 21 (-77 to 65) | 11 (-103 to 61) | |
| <1 year since vaccination | | – | – | – |  | 217 439 | 168 952 | 37 (21.9) |  | –42 (-311 to 51) | –52 (-319 to 45) | |
| ≥1 year since vaccination | | – | – | – |  | 178 595 | 344 951 | 32 (9.3) |  | 57 (-7 to 83) | 51 (-28 to 81) | |
|  | 1-3 primary doses Without 2aP preschool booster (reference) | | | |  | 1-3 primary doses with 2aP preschool booster | | |  |  | |  |
| Laboratory-confirmed pertussis | 40 893 | | 39 150 | 8 (20.4) |  | 230 478 | 538 263 | 71 (13.2) |  | 26 (-58 to 65) | 17 (-80 to 62) | |
| <1 year since vaccination | – | | – | – |  | 230 377 | 178 333 | 38 (21.3) |  | –63 (-386 to 46) | –72 (-380 to 38) | |
| ≥1 year since vaccination | – | | – | – |  | 187 700 | 359 930 | 33 (9.2) |  | 64 (17 to 84) | 59 (2 to 83) | |
| Frequencies of 1 or 2 are masked following the data privacy policy of Findata. Data sources: Digital and Population Data Services Agency, National Infectious Disease Register, National Vaccination Register, Care Register for Health Care, The Social Insurance Institution (KELA) Benefits register | | | | | | | | | | | | |

| **Table E. Effectiveness of 2aP preschool booster against laboratory-confirmed pertussis by previous vaccination status among children over 4.25 years old and born Jan 1, 2009–Sep 30, 2015, in Apr 2009 – Dec 2019 — cohort study** | | | | | | | | | | |
| --- | --- | --- | --- | --- | --- | --- | --- | --- | --- | --- |
|  |  |  |  |  |  |  |  |  |  |  |
| **Outcome** | **No primary doses and no 2aP preschool booster (reference)** | | |  | **3 primary doses with 2aP preschool booster** | | |  | **VE (95% Cl)** | |
|  | **N** | **Follow-up (years)** | **Cases, n (incidence per 100 000 person-years)** |  | **N** | **Follow-up (years)** | **Cases, n (incidence per 100 000 person-years)** |  | **Unadjusted** | **Adjusted** |
| **Laboratory-confirmed pertussis** | 3 640 | 9 137 | 11 (120.4) |  | 312 666 | 1 053 271 | 138 (13.1) |  | 89 (79 to 94) | 88 (78 to 94) |
| <1 year since vaccination | − | − | − |  | 312 651 | 250 591 | 51 (20.4) |  | 82 (52 to 93) | 82 (53 to 93) |
| ≥1 year since vaccination | – | – | – |  | 273 626 | 802 680.1 | 87 (10.8) |  | 91 (84 to 95) | 91 (83 to 95) |
|  | **3 primary doses Without 2aP preschool booster (reference)** | | |  | **3 primary doses with 2aP preschool booster** | | |  |  | |
| **Laboratory-confirmed pertussis** | 51 917 | 54 390 | 10 (18.4) |  | 312 666 | 1 053 271 | 138 (13.1) |  | 20 (-55 to 59) | 14 (-67 to 56) |
| <1 year since vaccination | – | – | – |  | 312 651 | 250 591 | 51 (20.4) |  | –41 (-273 to 47) | –47 (-275 to 43) |
| ≥1 year since vaccination | – | – | – |  | 273 626 | 802 680 | 87 (10.8) |  | 48 (-6 to 75) | 44 (-17 to 74) |
|  | **1-3 primary doses Without 2aP preschool booster (reference)** | | |  | **1-3 primary doses with 2aP preschool booster** | | |  |  |  |
| **Laboratory-confirmed pertussis** | 55 957 | 60 074 | 12 (20.0) |  | 328 319 | 1 092 288 | 142 (13.0) |  | 29(-31 to 61) | 24(-41 to 59) |
| <1 year since vaccination | – | – | – |  | 328 191 | 262 196 | 52 (19.8) |  | –53 (-301 to 42) | –58 (-298 to 37) |
| ≥1 year since vaccination | – | – | – |  | 285 329 | 830 092 | 90 (10.8) |  | 57 (17 to 77) | 54 (10 to 76) |
| Frequencies of 1 or 2 are masked following data privacy policies. | | | | | | | | | | |
| Data sources: Digital and Population Data Services Agency, National Infectious Disease Register, National Vaccination Register, Care Register for Health Care, The Social Insurance Institution (KELA) Benefits register | | | | | | | | | | |

An additional analysis not excluding children living in the municipalities with inadequate vaccination data resulted in a lower VE at 42% (95% CI 15 to 61%), indicating that some vaccinations were probably missing from the data. Preplanned sensitivity analyses excluding previous laboratory-confirmed and clinically diagnosed pertussis cases or extending the follow-up to start earlier produced similar estimates to the primary cohort analysis. (Table F)

| **Table F. Effectiveness of 2aP preschool booster against laboratory-confirmed pertussis in 2011–2019 — cohort design** | | | | | | | | | | |
| --- | --- | --- | --- | --- | --- | --- | --- | --- | --- | --- |
|  |  |  |  |  |  |  |  |  |  |  |
| **Outcome/Analysis** | **Without 2aP preschool booster (reference)** | | |  | **With 2aP preschool booster** | | |  | **VE (95% Cl)** | |
|  | **N** | **Follow-up (years)** | **Positive for B. pertussis, n (incidence per 100 000 person-years)** |  | **N** | **Follow-up (years)** | **Positive for B. pertussis, n (incidence per 100 000 person-years)** |  | **Unadjusted** | **Adjusted** |
| Additional analysis: Laboratory-confirmed pertussis among children aged 4.25–9.25 years, municipalities with inadequate reporting not excluded | 95 773 | 136 4267 | 33 (24.2) |  | 461 475 | 1 616 730 | 210 (13.0) |  | 44 (19 to 61) | 42 (15 to 61) |
| Sensitivity analysis: Laboratory-confirmed pertussis among children aged 4.25–9.25 years who did not have a laboratory-confirmed nor clinically diagnosed pertussis before the start of follow-up | 85 307 | 103 585 | 31 (29.9) |  | 436 384 | 1 509 945 | 193 (12.8) |  | 55 (34 to 70) | 51 (28 to 67) |
| Time split | 85 307 | 103 585 | 31 (29.9) |  | – | – | – |  | – | – |
| <1 year since vaccination | – | – | – |  | 436 144 | 354 172 | 59 (16.7) |  | 37 (-17 to 66) | 33 (-24 to 63) |
| ≥1 year since vaccination | – | – | – |  | 390 308 | 1 155 773 | 134 (11.6) |  | 64 (45 to 77) | 61 (39 to 75) |
| Sensitivity analysis: Laboratory-confirmed pertussis among children aged 3.75–9.25 years | 478 126 | 277 265 | 58 (20.9) |  | 434 513 | 1 559 402 | 212 (13.6) |  | 48 (23 to 65) | 44 (18 to 62) |
| Time split | 478 126 | 277 265 | 58 (20.9) |  | – | – | – |  | – | – |
| <1 year since vaccination | – | – | – |  | 434 513 | 412 734 | 79 (19.1) |  | 27 (-22 to 56) | 23 (-29 to 54) |
| ≥1 year since vaccination | – | – | – |  | 387 950 | 1 146 668 | 133 (11.6) |  | 64 (44 to 77) | 61 (39 to 75) |
| Additional sensitivity analysis: Clinically diagnosed pertussis among children aged 4.25–9.25 years who did not have lab-confirmed nor clinically diagnosed pertussis before start of follow-up | 85 307 | 116 248 | 16 (13.8) |  | 436 383 | 1 809 045 | 148 (8.2) |  | 41 (2 to 64) | 40 (-1 to 64) |
| Time split | 85 307 | 116 247 | 16 (13.8) |  | – | – | – |  | – | – |
| <1 year since vaccination | – | – | – |  | 436 143 | 354 190. | 26 (7.3) |  | 59 (6 to 83) | 59 (4 to 83) |
| ≥1 year since vaccination | – | – | – |  | 390 339 | 1 454 855 | 122 (8.4) |  | 27 (-32 to 60) | 26 (-37 to 60) |
| Frequencies of 1 or 2 are masked following data privacy policies. | | | | | | | | | | |
| Data sources: Digital and Population Data Services Agency, National Infectious Disease Register, National Vaccination Register, Care Register for Health Care, Register of Primary Health Care Visits, The Social Insurance Institution (KELA) Benefits register | | | | | | | | | | |

In a sensitivity analysis of preschool booster vaccine effectiveness against laboratory-confirmed pertussis by age, the adjusted estimates ranged from 36% (95% CI -108 to 80%) at 7 years of age to 78% (95% CI 37 to 93%) at 10 years of age. Confidence intervals were wide and no trend of waning effectiveness could be seen at least before 12 years of age. (Table G)

| **Table G. Effectiveness of 2aP preschool booster against laboratory-confirmed pertussis by age among children over 4.25 years old and born between Jan 1, 2007 – Sep 1, 2015, in 2011–2019 — cohort study** | | | | | | | | | | |
| --- | --- | --- | --- | --- | --- | --- | --- | --- | --- | --- |
|  |  |  |  |  |  |  |  |  |  |  |
| **Outcome** | **Without 2aP preschool booster (reference)** | | |  | **With 2aP preschool booster** | | |  | **VE (95% Cl)** | |
|  | **N** | **Follow-up (years)** | **Positive for B. pertussis, n (incidence per 100 000 person-years)** |  | **N** | **Follow-up (years)** | **Positive for B. pertussis, n (incidence per 100 000 person-years)** |  | **Unadjusted** | **Adjusted** |
|  |  |  |  |  |  |  |  |  |  |  |
| Laboratory-confirmed pertussis | 85 421 | 116 370 | 38 (32.7) |  | – | – | – |  | – | – |
| at 4 years old | – | – | (31.7) |  | 427 532 | 299 065 | 49 (16.4) |  | 45 (-7 to 72) | 41 (-16 to 70) |
| at 5 years old | – | – | (34.1) |  | 395 423 | 366 855 | 26 (7.1) |  | 79 (55 to 90) | 77 (51 to 89) |
| at 6 years old | – | – | (26.9) |  | 344 563 | 318 320 | 45 (14.1) |  | 47 (-34 to 79) | 43 (-42 to 77) |
| at 7 years old | – | – | (22.1) |  | 292 689 | 266 584 | 35 (13.1) |  | 40 (-96 to 82) | 36 (-108 to 80) |
| at 8 years old | – | – | (33.5) |  | 240 956 | 214 745 | 27 (12.6) |  | 62 (-27 to 88) | 59 (-35 to 88) |
| at 9 years old | – | – | (30.6) |  | 188 497 | 161 968 | 30 (18.5) |  | 40 (-149 to 86) | 36 (-167 to 85) |
| at 10 years old | – | – | (87.6) |  | 135 227 | 109 516 | 19 (17.4) |  | 80 (42 to 93) | 78 (37 to 93) |
| at 11 years old | – | – | (35.3) |  | 83 777 | 59 303 | 10 (16.9) |  | 56 (-242 to 94) | 52 (-273 to 94) |
| at 12 years old | – | – | (0) |  | 34 269 | 14 247 | 5 (35.1) |  | -Inf (-Inf to -Inf) | -Inf (-Inf to -Inf) |
| Frequencies of 1 or 2 are masked following data privacy policies. | | | | | | | | | | |
| Data sources: Digital and Population Data Services Agency, National Infectious Disease Register, National Vaccination Register, Care Register for Health Care, The Social Insurance Institution (KELA) Benefits register | | | | | | | | | | |

For clinical diagnoses, the overall impact was estimated as a reduction of 44% (95% CI 25 to 59%) in the incidence rates of children over 4.25 years of age, and the indirect impact for children under 3.75 years of age was estimated as 19% (95% CI 6 to 30%) (Table H).

| **Table H. Impact of 2aP preschool booster against pertussis clinically diagnosed at hospital setting among children in 1995–2002 and 2012–2019 — before-after comparison** | | | | | | | |
| --- | --- | --- | --- | --- | --- | --- | --- |
|  |  |  |  |  |  |  |  |
|  | **1995–2002 (reference)** | | | **2012–2019 (target)** | | | **Impact of preschool booster (95% CI)** |
|  | **n** | **Follow-up (years)** | **Incidence per 100 000 person-years** | **n** | **Follow-up (years)** | **Incidence per 100 000 person-years** |  |
| **Overall impact against clinically diagnosed pertussis** | | | |  |  |  |  |
| Children aged ≥ 4.25 years | 127 | 2 079 861 | 6.1 | 67 | 1 970 635 | 3.4 | 44 (25 to 59) |
| 4.25 – < 6 years old | 38 | 796 641 | 4.8 | 22 | 758 859 | 2.9 | 39 (-2 to 65) |
| 6 – <8 years old | 44 | 684 864 | 6.4 | 26 | 648 982 | 4.0 | 38 (0 to 62) |
| 8 – < 13 years old | 45 | 598 357 | 7.5 | 19 | 562 794 | 3.4 | 55 (25 to 74) |
| Children aged 4.25 – < 9.25 years | 107 | 1 780 573 | 6.0 | 55 | 1 690 197 | 3.3 | 46 (25 to 61) |
| **Indirect impact against clinically diagnosed pertussis** | | | |  |  |  |  |
| Children aged ≤ 3.75 years | 428 | 1 809 703 | 23.7 | 329 | 1 710 350 | 19.2 | 19 (6 to 30) |
| ≤ 2 years old | 404 | 948 477 | 42.6 | 304 | 888 077 | 34.2 | 20 (7 to 31) |
| >2 – ≤3.75 years old | 24 | 861 225 | 2.8 | 25 | 822 272 | 3.0 | –9 (-92 to 38) |
| Target years 2012–2015 | 428 | 1 809 703 | 23.7 | 140 | 898 862 | 15.6 | 34 (21 to 46) |
| Target years 2016–2019 | 428 | 1 809 703 | 23.7 | 189 | 811 488 | 23.3 | 2 (-17 to 17) |
| Frequencies of 1 or 2 are masked following data privacy policies. | | | | | | | |
| Data sources: Digital and Population Data Services Agency, Care Register for Health Care, National Infectious Disease Register | | | | | | | |

Incidence rates of adenovirus are demonstrated in Fig A. Of the adenovirus cases, the percentage of cases diagnosed with PCR or culture increased from 12% to 36% between the periods 1995-2002 and 2011-2019, which is much larger increase than seen in pertussis diagnostics. Therefore, we did not consider it relevant to use adenovirus as a negative control for the impact against pertussis.


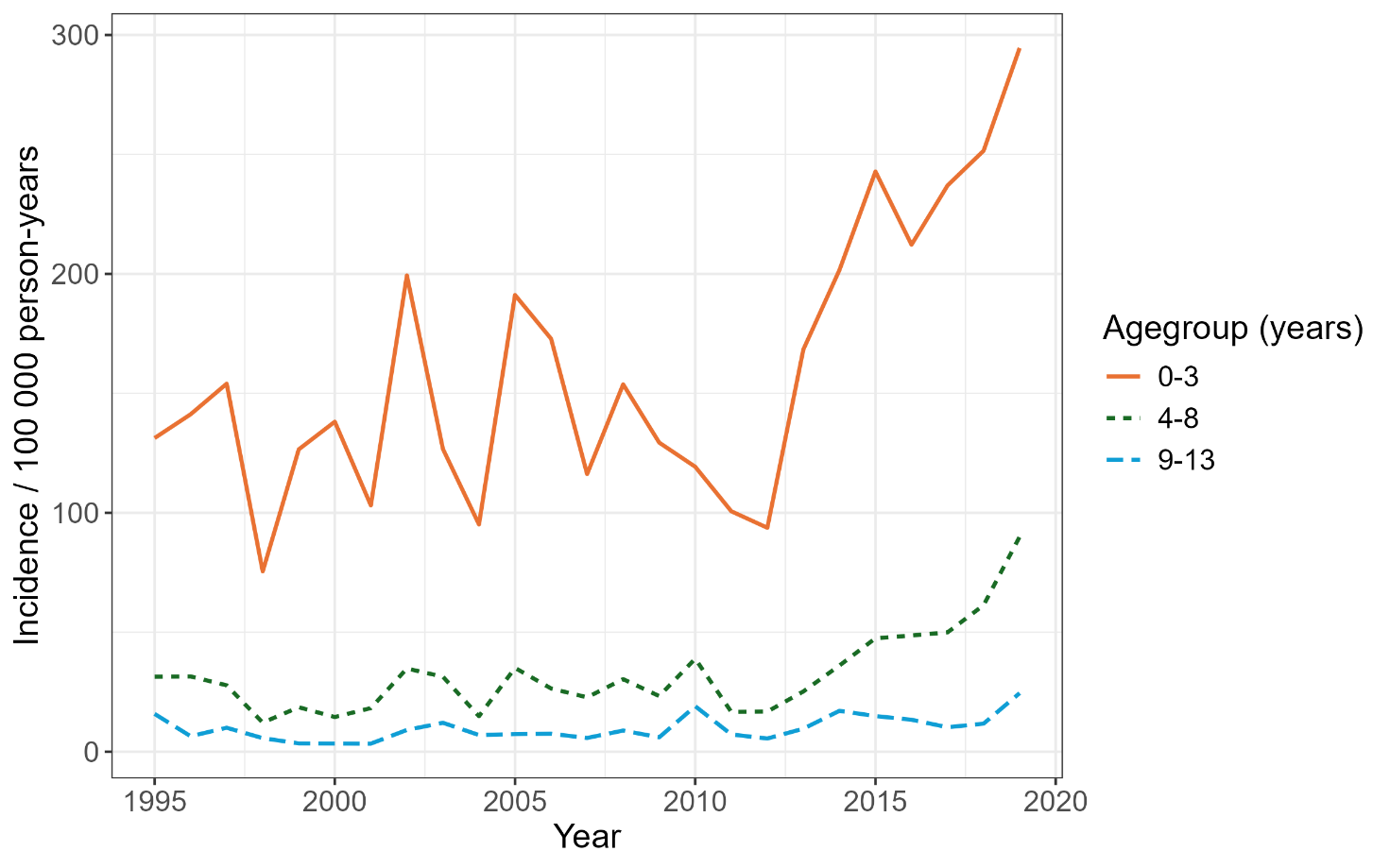


**Fig A. Incidence of laboratory confirmed adenovirus among all study children born 1995–2019 by age-group (years)**

Data sources: Digital and Population Data Services Agency, National Infectious Disease Register


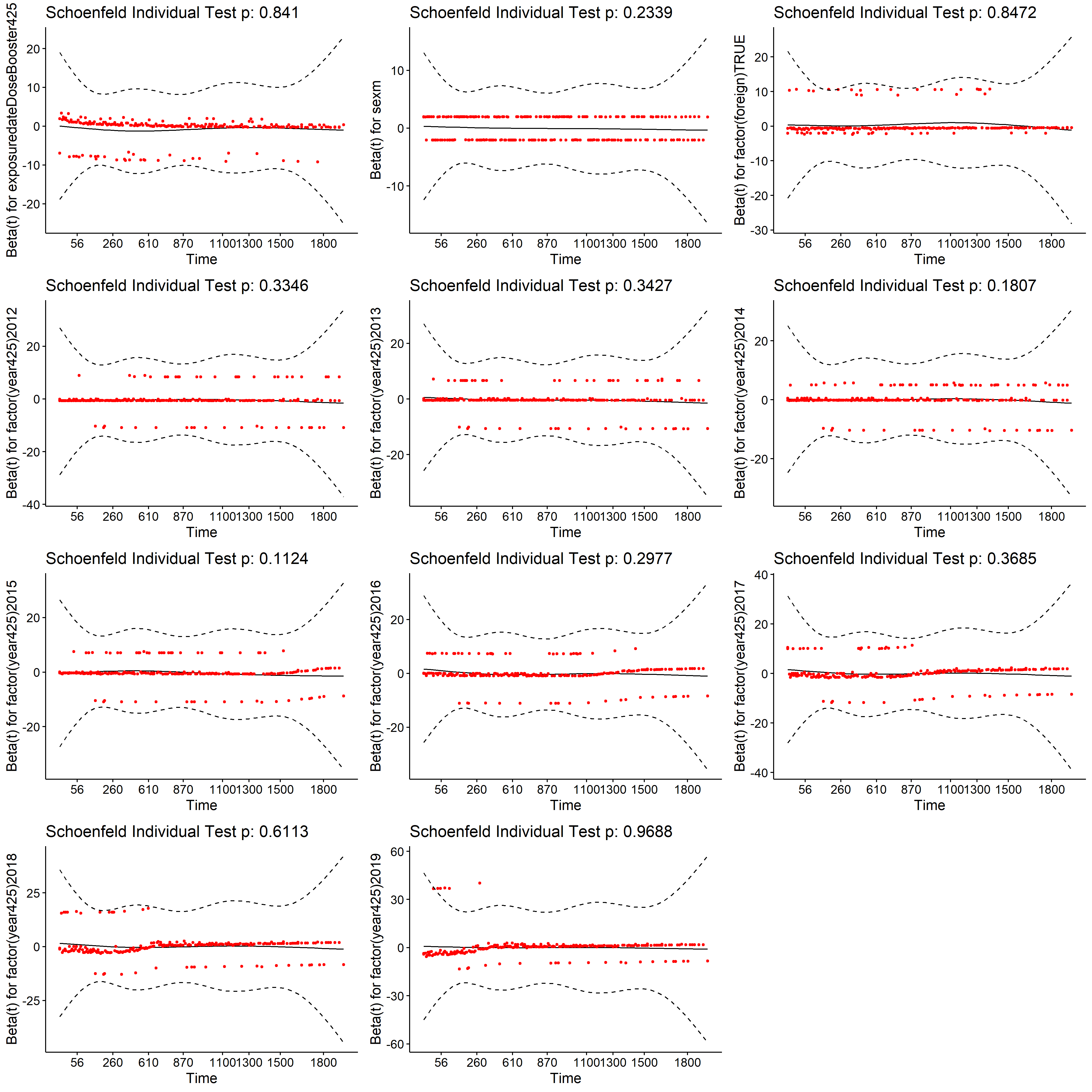


**Fig B. Schoenfeld individual tests of proportional hazards for categorical covariates**
