# Supplementary material for: Effectiveness and impact of the 2-component acellular pertussis vaccine as a preschool booster in Finland – A register-based study
Source: PLOS Glob Public Health. 2026 Jul 29;6(7):e0006800. doi: 10.1371/journal.pgph.0006800 (PMC13419238; doi:10.1371/journal.pgph.0006800)
Supplement: S1 Checklist — The STROBE Statement checklist is reproduced under the terms of the Creative Commons Attribution 4.0 International (CC BY 4.0) License. See the STROBE Initiative for further information. (DOCX) [file pgph.0006800.s004.docx]

# S4 STROBE Checklist

**Title and abstract**

***1 (a) Indicate the study’s design with a commonly used term in the title or the abstract***

Page 1: ’Effectiveness and impact of the 2-component acellular pertussis vaccine as a preschool booster in Finland – A register-based study’

***(b) Provide in the abstract an informative and balanced summary of what was done and what was found***

Page 3: ‘The 2aP preschool booster seems to provide moderate and sustained protection against pertussis compared to children who did not receive the booster and high protection when combined with the 2+1 primary series compared to children who received no pertussis vaccinations. Effectiveness was notably higher against PCR/culture-confirmed cases throughout the follow-up, likely due to lower specificity of serology and differences in diagnostic practices by disease manifestations. These findings support the continued use of the 2aP preschool booster in Finland. ’

**Introduction**

***Background/rationale***

***2 Explain the scientific background and rationale for the investigation being reported***

Page 3-4: ‘Pertussis, caused by *Bordetella pertussis,* remains a leading cause of vaccine-preventable childhood morbidity and mortality worldwide, despite widespread vaccination [1]. Most high-income countries transitioned from whole-cell (wP) to acellular (aP) pertussis vaccines in the late 1990s and early 2000s, mainly driven by safety and tolerability considerations. Although pertussis vaccines provide good short-term protection against disease [2], *B. pertussis* continues to circulate, and epidemics still occur. During the last decades, even countries with high vaccine coverage have experienced a resurgence of pertussis incidence [3]. Previous studies have suggested that immunity following aP vaccination wanes over time and that aP vaccines have limited ability in preventing transmission [2]. Nevertheless, as more evidence is generated in this area, more recent empirical and modeling studies have challenged this view, suggesting a longer duration of protection [4].

Understanding the effectiveness and duration of protection conferred by pertussis vaccines is essential for optimizing vaccination strategies, including timing of booster doses and other measures to protect the most vulnerable to the disease.’

***Objectives***

***3 State specific objectives, including any prespecified hypotheses***

Page 4: ‘Primary objective of this study was to evaluate the effectiveness of a 2aP vaccine administered as a preschool booster at 4 years of age against laboratory-confirmed pertussis during a follow-up of 5 years. ‘

**Methods**

***Study design***

***4 Present key elements of study design early in the paper***

Page 4: ‘This was a nationwide population-based register study in Finland.’, ‘Cohort study: Cohort design was used to estimate the effectiveness of 2aP preschool pertussis booster vaccine against pertussis outcomes. We followed-up children turning 4.25 years old during 2011–2019. (Fig 1)’

***Setting***

***5 Describe the setting, locations, and relevant dates, including periods of recruitment, exposure, follow-up, and data collection***

Page 4:’ This was a nationwide population-based register study in Finland. The study population comprised all permanent residents aged under 14 years between 1995 and 2019, as identified from the Finnish Population Information System. We used routinely collected data from several national health and administrative registers linked via unique personal identity code assigned to each resident. Detailed descriptions of the data sources are provided in Supporting Information (S1 Study protocol). Data was extracted and then delivered to the study team in 2024 by the Finnish Social and Health Data Permit Authority, Findata.’

***Participants***

***6 (a) Give the eligibility criteria, and the sources and methods of selection of participants. Describe methods of follow-up***

Page 5-7: ‘The population eligible for the cohort analysis consisted of children born from January 2007 through September 2015, recorded by the Digital and population data services agency (DVV) as permanent residents in Finland. Permanent residency in Finland was defined as having no sign of living abroad between age 3 years and the start age of the follow-up. Each subject had to be alive at the start age of the follow-up to be included in the analysis cohort.’

‘The follow-up period for each child started from the age of 4.25 years and lasted until the first outcome event of interest, any pertussis vaccination other than 2aP, any pertussis vaccination after the 2aP preschool booster dose or after 6 years of age, emigration from Finland, death, or Dec 31, 2019, whichever came first. Competing risks were not considered.’

***Variables***

***7 Clearly define all outcomes, exposures, predictors, potential confounders, and effect modifiers. Give diagnostic criteria, if applicable***

Page 7: ‘Exposure in the cohort analyses was the preschool 2aP pertussis vaccination (in combination with diphtheria, tetanus, and inactivated poliomyelitis vaccines, DT2aP-IPV(Tetravac®), and occasionally also with the Hib-component(Pentavac®), used as time-dependent variable. ‘

Page 10: ‘Primary outcome laboratory-confirmed pertussis was defined as a case of pertussis registered in the National infectious disease register (NIDR) based on detection of *B. pertussis*, by polymerase chain reaction (PCR), culture, serology or other methods.’

Page 11: ‘Table 1. Definitions of demographic and covariate variables related to a study subject.’

***Data sources/ measurement***

***8* For each variable of interest, give sources of data and details of methods of assessment (measurement). Describe comparability of assessment methods if there is more than one group***

Page 6: ‘To define the exposure, information was retrieved from NVR, in which all vaccinations administered in public primary health care and increasingly also in private health care have been recorded since 2009–2011. NVR was used to collect all individual pertussis vaccinations (administration date, vaccine, trade name) of the study children born 2007 and later, until December 31, 2019.’

Page 10: ‘Primary outcome laboratory-confirmed pertussis was defined as a case of pertussis registered in the National infectious disease register (NIDR) based on detection of *B. pertussis*, by polymerase chain reaction (PCR), culture, serology or other methods.’

Page 11: Table1: example from a covariate:

| **Variable** | **Definition** | **Data source** | **Analysis/role** |
| --- | --- | --- | --- |
| Number of inpatient hospitalizations | Number of inpatient hospitalizations between 14 and 14+365 days before index date and daily with a lag of 14 days during follow-up. Categories for the number of inpatient hospitalizations: 0 (reference), 1-4, ≥5. | HILMO | Covariate in cohort analyses |

***Bias***

***9 Describe any efforts to address potential sources of bias***

Examples:

Page 12: ‘The regression model adjusted for sex, region of residence, foreign background, number of chronic diseases, number of inpatient hospitalizations and cohort entry year, using age as the time scale.’

Page 13: ‘Children residing in municipalities during a period when the number of reported infant DTaP-IPV-Hib vaccinations per capita was temporally lower than expected, likely due to issues in the data transfer to NVR, were excluded from the cohort analysis.’

Page 14: ‘Several preplanned sensitivity analyses were conducted to validate the study results. First, adenovirus infection detected in the NIDR was used as a negative control outcome, to assess residual confounding’

Potential sources of bias are also addressed extensively in Discussion, especially in ‘’Strengths and Limitations’’.

Page 27: ‘First, the nationwide population-based design which included nearly all eligible children, ensured negligible loss to follow-up through comprehensive registers, minimized selection bias and enhanced generalizability. Second, a large sample size reduced random error and, together with extensive covariate data, enabled controlling for many potential confounders in adjusted Cox regression model. Third, sensitivity analyses using alternative definitions of exposure, outcomes, and follow-up produced minimal changes in the results, indicating good internal validity. The use of adenovirus infection as a negative control outcome further suggested limited residual confounding. Fourth, the Finnish register data are comprehensive and highly reliable. Nearly all childhood vaccinations are administered within the public system and reported to the NVR; municipalities with incomplete reporting were excluded. Outcome ascertainment was strengthened by using primarily laboratory-confirmed cases, with mandatory reporting of all positive results to the NIDR’.

***Study size***

***10 Explain how the study size was arrived at***

Page 12: ‘The number of children eligible during our study period determined the sample size. Sample size calculations for the primary endpoint, laboratory-confirmed pertussis, are provided in S1 Study protocol.’

***Quantitative variables***

***11 Explain how quantitative variables were handled in the analyses. If applicable, describe which groupings were chosen and why***

From page 11: Demographics and covariates:

| Number of chronic diseases | Number of relevant chronic diseases. Factor with levels 0 (reference), 1, ≥2. Determined at index date and daily during follow-up: reimbursement decisions effective at and/or after index date were considered. Thirteen different categories were defined based on reimbursement codes and attached diagnoses codes (S2 Appendix): lung disease, severe disorder of the immune system, diabetes, undernutrition, actively treated cancer, severe kidney disease, inflammatory bowel disease, adrenal insufficiency, other endocrinological disease, severe heart disease, neurological condition, severe mental disorders and Down syndrome and severe disability | The Social Insurance Institution (KELA) Benefits register | Covariate in cohort analyses |
| --- | --- | --- | --- |
| Number of inpatient hospitalizations | Number of inpatient hospitalizations between 14 and 14+365 days before index date and daily with a lag of 14 days during follow-up. Categories for the number of inpatient hospitalizations: 0 (reference), 1-4, ≥5. | HILMO | Covariate in cohort analyses |

***Statistical methods***

***12 (a) Describe all statistical methods, including those used to control for confounding***

Page 12: ‘The effect measure of interest was VE quantified as (1 - hazard ratio (HR)) * 100%. Cox regression was used for estimation, treating vaccination as a time-dependent variable. The regression model adjusted for sex, region of residence, foreign background, number of chronic diseases, number of inpatient hospitalizations and cohort entry year, using age as the time scale.’

***(b) Describe any methods used to examine subgroups and interactions***

Page 13: ‘Follow-up among the vaccinated was stratified by time since receipt of the booster vaccination: <1, 1–<2, 2–<4, 4–<6, 6–<8, and ≥8 years. For each interval, the hazard of laboratory-confirmed pertussis was compared between children of the same age who received the preschool booster and those who did not.’

***(c) Explain how missing data were addressed***

Page 13: ‘Missing data are usually not considered in the Finnish register data. If a person lacked a record, such as a positive laboratory finding, diagnosis, prescription or visit, he/she was considered not to have the laboratory-confirmed infection, exposure, outcome, comorbidity, treatment or contact with a professional in question. Some variables occasionally had missing values.

Children residing in municipalities during a period with the number of reported infant DTaP-IPV-Hib vaccinations per capita was temporally lower than expected, likely due to issues in data transfer to NVR, were excluded from the cohort analysis…’

***(d) If applicable, explain how loss to follow-up was addressed***

Not addressed as due to the nationwide population-based design with comprehensive register follow-up, there was practically no loss to follow-up.

***(e) Describe any sensitivity analyses***

Page 14: ‘Several preplanned sensitivity analyses were conducted to validate the study results. First, adenovirus infection detected in the NIDR was used as a negative control outcome, to assess residual confounding.’

**Results**

***13* Participants a) Report numbers of individuals at each stage of study—e.g. numbers potentially eligible, examined for eligibility, confirmed eligible, included in the study, completing follow-up, and analyzed***

Page 15:

**Fig 3. Selection of children into the primary analysis and effectiveness analysis by previous vaccination status, after exclusions**

1. ***Give reasons for non-participation at each stage***

See above

***14* Descriptive data (a) Give characteristics of study participants (e.g. demographic, clinical, social) and information on exposures and potential confounders***

Page 18: ‘In the primary cohort analysis, the children with foreign background were overrepresented among those without the preschool booster (Table 4).’

***(b) Indicate number of participants with missing data for each variable of interest***

not applicable, see 12c about handling missing data.

*(c)* ***Summarize follow-up time (e.g., average and total amount)***

Pae 16: ‘During the 1 615 237 person-years of follow-up (median 3.5, maximum 5 years) in primary cohort analysis, 225 laboratory-confirmed pertussis cases were identified.’

***15* Outcome data: Report*** ***numbers of outcome events or summary measures over time***

Page 17:


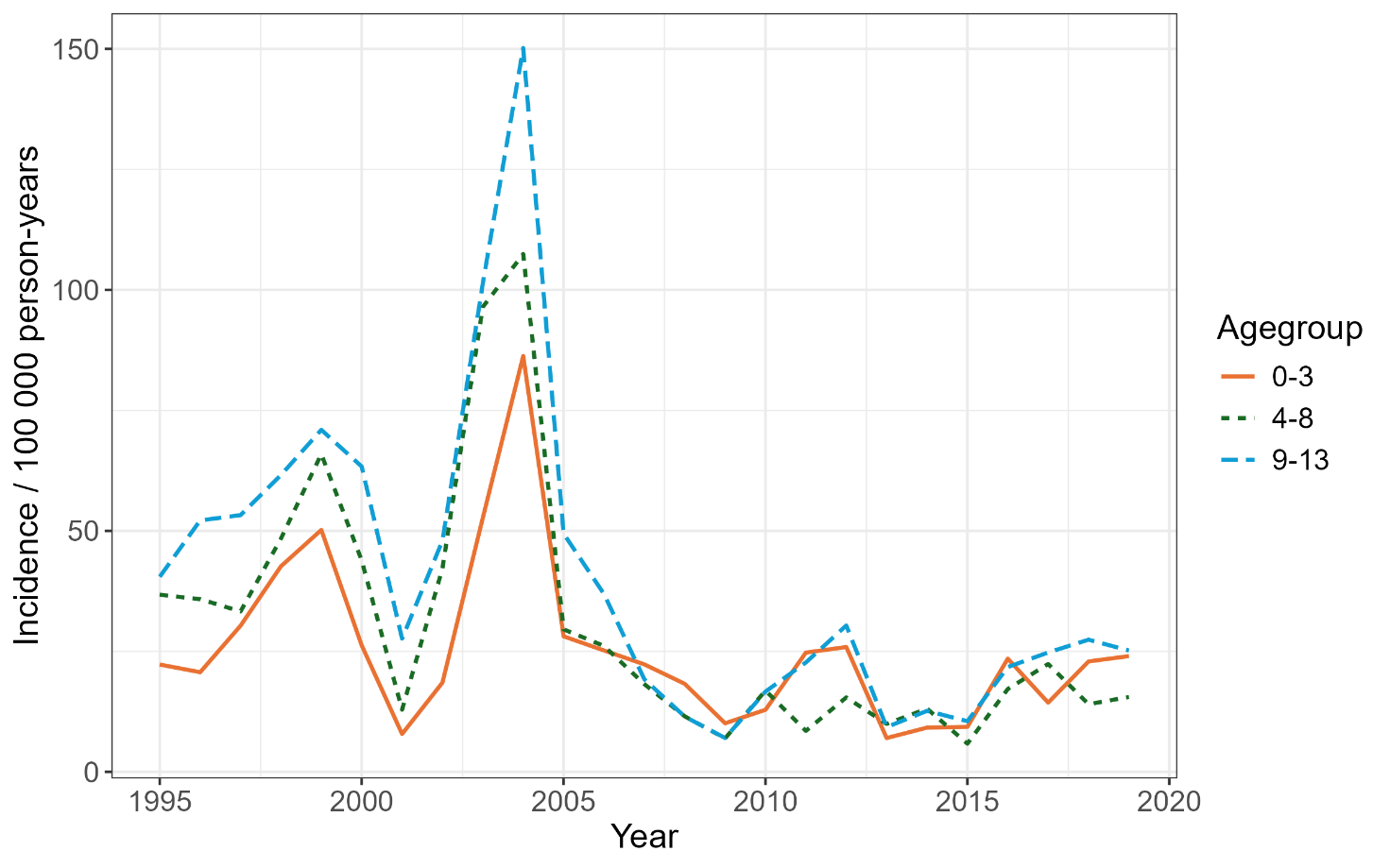


**Fig 4. Incidence of laboratory-confirmed pertussis among all study children during 1995–2019 by age-group (years).**

***16 Main results: (a) Give unadjusted estimates and, if applicable, confounder-adjusted estimates and their precision (e.g., 95% confidence interval). Make clear which confounders were adjusted for and why they were included***

Page 19: ‘The vaccine effectiveness estimates reported in the text are adjusted for age, sex, region of residence, foreign background, number of chronic diseases, number of inpatient hospitalizations and year of entering the study cohort, unless otherwise stated. These potential confounders available in the national registers were chosen because they were expected to reflect the children’s susceptibility for illness and affect their level of infection pressure. The unadjusted estimates can be found from the tables.’ ‘Effectiveness of the preschool booster dose against any laboratory-confirmed pertussis in the primary analysis of children 4.25–9.25 years of age was 51% (95% confidence interval, 95% CI: 27 to 67%) (Table 6).’

***(b) Report category boundaries when continuous variables were categorized***

Not applicable.

***(c) If relevant, consider translating estimates of relative risk into absolute risk for a meaningful time period***

Absolute risks could not be calculated from the cox regression models hazard ratio estimates and would have required a different approach.

***17 Other analyses: Report other analyses done—e.g. analyses of subgroups and interactions, and sensitivity analyses***

Page 23: ‘In the additional analysis of the effectiveness of the full 3-dose (2+1) primary vaccination series without booster against any laboratory-confirmed pertussis, the effectiveness estimates were high at 92% (95% CI 85 to 96%) for 0–3 years of age, and 81% (95% CI 51 to 93%) for children over 4 years old (Table 8). Effectiveness estimates in time intervals since vaccination were also high and showed only a small trend of waning (Table 8).’

**Discussion**

***18 Key results***

***Summarize key results with reference to study objectives***

Page 24: This is the first study to provide real-world evidence of aP vaccine effectiveness in Finland, and specifically the first to evaluate the real-world effectiveness of the 2aP vaccine as a preschool booster globally. In the primary cohort analysis, the DT2aP-IPV preschool booster was found to be effective against laboratory-confirmed pertussis with a vaccine effectiveness of 51% (95% CI: 27 to 67%)…’

***19 Limitations***

***Discuss limitations of the study, taking into account sources of potential bias or imprecision. Discuss both direction and magnitude of any potential bias***

Page 27: ‘Nevertheless, several limitations should be acknowledged. The number of pertussis cases in our primary cohort was low, leading to wide confidence intervals and potential random error; however, estimates for the primary and most secondary objectives remained statistically significant. As in all observational studies, residual confounding and unmeasured effect modifiers may remain...’

***20 Interpretation***

***Give a cautious overall interpretation of results considering objectives, limitations, multiplicity of analyses, results from similar studies, and other relevant evidence***

Page 25: ‘The primary analysis comparing children with and without the preschool booster indicated moderate effectiveness against any laboratory-confirmed pertussis. Analyses comparing the full vaccination series (3-dose (2+1) primary series and preschool booster) and the 3-dose (2+1) primary series with unvaccinated showed high absolute vaccine effectiveness, consistent with previous literature [2, 18]...’

***21 Generalizability***

***Discuss the generalizability (external validity) of the study results***

Page 23: ‘Overall, these results are generalizable to other countries using similar aP vaccination programs for children.’

**Other information**

***Funding***

***22 Give the source of funding and the role of the funders for the present study and, if applicable, for the original study on which the present article is based***

Not applicable as disclosure of funding is not part of the manuscript in Plos Medicine instructions. Funding information is submitted in another form.
