# Supplementary material for: Effectiveness and impact of the 2-component acellular pertussis vaccine as a preschool booster in Finland – A register-based study
Source: PLOS Glob Public Health. 2026 Jul 29;6(7):e0006800. doi: 10.1371/journal.pgph.0006800 (PMC13419238; doi:10.1371/journal.pgph.0006800)
Supplement: Attachment — Submitted filename: Response to Reviewers.docx [file pgph.0006800.s005.docx]

# Response to Reviewers

Jussi Halme
FVR-Finnish Vaccine Research
[jussi.halme@fvr.fi](mailto:jussi.halme@fvr.fi) │+358 406819219
1.4.2026

Dear editors,

Our research article entitled **‘’Effectiveness and impact of the 2-component acellular pertussis vaccine as a preschool booster in Finland – A register-based study’’** was originally submitted to *PLOS Medicine.* Following editorial assessment, the manuscript was not accepted for publication. However, the editors recommended transfer to *PLOS Global Public Health*.

As no reviewer comments or content-related queries were provided during the initial evaluation, we have not made any changes to the manuscript or supporting materials. The only modification is that the figures are now submitted as separate files, in accordance with the journal’s submission requirements.

We chose to proceed with the transfer as we believe the manuscript aligns well with the scope and aims of *PLOS Global Public Health.* The study addresses infectious disease prevention, and public health policy. By providing real-world evidence on the effectiveness and impact of a pertussis booster strategy within a national immunization program, our work offers insights to researchers, clinicians, and policymakers working to optimize vaccination strategies and control pertussis globally.

We appreciate your consideration of our work.

Sincerely,

Jussi Halme, M.D.
on behalf of all co-authors
