# Supplementary material for: Effectiveness and impact of the 2-component acellular pertussis vaccine as a preschool booster in Finland – A register-based study
Source: PLOS Glob Public Health. 2026 Jul 29;6(7):e0006800. doi: 10.1371/journal.pgph.0006800 (PMC13419238; doi:10.1371/journal.pgph.0006800)
Supplement: Attachment — Submitted filename: Response to reviewers-revision 18.6.2026.docx [file pgph.0006800.s006.docx]

# Response to Reviewers

Jussi Halme
FVR-Finnish Vaccine Research
[jussi.halme@fvr.fi](mailto:jussi.halme@fvr.fi) │+358 406819219
18.6.2026

Dear editors and reviewers,

Thank you for your review and comments. Below we provide answers highlighted in red for each question and comment.

**Journal Requirements:**

1. Please amend your detailed Financial Disclosure statement. This is published with the article. It must therefore be completed in full sentences and contain the exact wording you wish to be published.

i. Please clarify all sources of financial support for your study. List the grants, grant numbers, and organizations that funded your study, including funding received from your institution. Please note that suppliers of material support, including research materials, should be recognized in the Acknowledgements section rather than in the Financial Disclosure. 
ii. State the initials, alongside each funding source, of each author to receive each grant. For example: "This work was supported by the National Institutes of Health (####### to AM; ###### to CJ) and the National Science Foundation (###### to AM)."
iii. State what role the funders took in the study. If the funders had no role in your study, please state: “The funders had no role in study design, data collection and analysis, decision to publish, or preparation of the manuscript.”
iv. If any authors received a salary from any of your funders, please state which authors and which funders.

Answer: We have amended the financial disclosure by adding information about the Sanofi employees: ‘’This study was funded by Sanofi (No. PER000108). <https://www.sanofi.com/en>. Sanofi reviewed the study protocol and the manuscript. Authors SA, AS and JCV are employees of Sanofi and may hold shares and/or stock options.’’

2. In the online submission form, you indicated that “According to the Finnish law, the Act on the secondary use of health and social data, the data from national health registers used as a source of data in this study can only be published as anonymized aggregate results. Accessing individual-level data in scientific research is possible by requesting data from the Finnish Social and Health Data Permit Authority, Findata, responsible for granting permits for the secondary use of health data. Once a permit is granted and data extracted, the pseudonymized data can then be processed and analyzed by investigators in a secure operating environment. Requesting the permit and extraction of the data are subject to costs charged by Findata and register controllers. Detailed instructions for applying data permits and application forms are accessible from the Findata website: <https://findata.fi/en/permits/>. R code for conducting the analyses can be requested from the authors after obtaining relevant permission from Findata.”.

All PLOS journals now require all data underlying the findings described in their manuscript to be freely available to other researchers, either 
1. In a public repository, 
2. Within the manuscript itself, or 
3. Uploaded as supplementary information.

This policy applies to all data except where public deposition would breach compliance with the protocol approved by your research ethics board. If your data cannot be made publicly available for ethical or legal reasons (e.g., public availability would compromise patient privacy), please explain your reasons by return email and your exemption request will be escalated to the editor for approval. Your exemption request will be handled independently and will not hold up the peer review process, but will need to be resolved should your manuscript be accepted for publication. One of the Editorial team will then be in touch if there are any issues.

Answer: We answered to this in a separate email as instructed. However, as PLOS GPH has not responded to any of our emails, the email is also attached below:

We have complemented the data availability statement as you requested. Added sentence highlighted below.

''According to the Finnish law, the Act on the secondary use of health and social data, the data from national health registers used as a source of data in this study can only be published as anonymized aggregate results, **as publishing individual-level patient data would compromise patients privacy**. Accessing individual-level data in scientific research is possible by requesting data from the Finnish Social and Health Data Permit Authority, Findata, responsible for granting permits for the secondary use of health data. Once a permit is granted and data extracted, the pseudonymized data can then be processed and analyzed by investigators in a secure operating environment. Requesting the permit and extraction of the data are subject to costs charged by Findata and register controllers. Detailed instructions for applying data permits and application forms are accessible from the Findata website:  <https://findata.fi/en/permits/>. R code for conducting the analyses can be requested from the authors after obtaining relevant permission from Findata.''

We are requesting an exemption for making individual-level data publicly available for legal reasons  as publishing individual-level patient data would be illegal according to finnish law as it would compromise patients privacy.

3. We do not publish any copyright or trademark symbols that usually accompany proprietary names, eg (R), (C), or TM (e.g. next to drug or reagent names). Please remove all instances of trademark/copyright symbols throughout the text, including Tetravac® and Pentavac®.

Answer: Trademark symbols have now been removed from the manuscript.

4. Please upload a copy of Figure 5, which you refer to in your manuscript. Or, if the figure is no longer to be included as part of the submission please remove all reference to it within the text.

Answer: This was a typo. The text was supposed to refer to figure 3 (current figure 4). This has now been corrected.

**Reviewer #1:** Many thanks for the opportunity to review the manuscript "Effectiveness and impact of the 2-component acellular pertussis vaccine as a preschool booster in Finland – A register-based study" This comprehensive report of vaccine effectiveness of the acellular pertussis vaccine with a pre-school booster or not. The findings of a 2+1 schedule with a pre school booster is reassuring for the protectiveness of children against severe pertussis disease.

Some comments:
- I would suggest reducing the number of tables in the main paper and possibly include some as supplementary material

Answer: We have moved table 5 to s3 appendix and tables 2 and 3 were turned into a flowchart as suggested.

- Tables 1, 2 and 3 could be summarised into a flowchart of the exclusions and inclusions of children in the study that would included in the primary analysis as well as for the effectiveness analysis

Answer: Tables 2 and 3 were turned into a flowchart. Table 1 Definitions of demographic and covariate variables related to a study subject, was considered to not be suitable for a flowchart.

- Table 5 - I do not understand why only those born after 2011 are summarised here? The 2 cohorts are children born 1990-1998 and 2007-2015. Also, is it necessary to show all aspects of age distribution - was age normal or not normally distributed in the cohorts?

Answer: Information about the primary series doses scheduled at 3, 5 and 12 months was available only from 2009 onwards (National vaccination register was founded in 2009), but the first 2 years 2009-2010 have usually been considered not so reliable so they were excluded from this. We added two sentences to the manuscript to clarify this (lines in track changes version: 128-129 and 151-152). Register descriptions can be found in the S1 Study protocol as referenced in the manuscript.

Table 5 is now moved to S3 Appendix

- The discussion could be reduced in length and to concentrate on the main findings

Answer: We removed from the discussion a few paragraphs that we deemed not so relevant to the main findings

- Line 380 - is meant to be figure 3 not figure 5?

Answer: You are correct. This was a typo that has now been corrected.

**Reviewer #2:** The authors of “Effectiveness and impact of the 2-component acellular pertussis vaccine as a preschool booster in Finland – A register-based study” evaluated the effectiveness and duration of protection associated with the preschool DTaP-IPV preschool booster and its population-level impact after implementation in the NIP in Finland. The manuscript is well-written and the findings are interesting. However, I have a few comments:

Lines 83-84: While apparent after reading the paper, it would help to clarify in the introduction that, along with the change to aP-containing vaccine given at 3, 5, and 12 months in 2005, the preschool booster at 4 years of age was added to the NIS in 2005 also. Furthermore, while it is clear from Figure 1 that children did not receive pertussis containing vaccine after 24 months of age until 2003 when the aP containing booster was introduced at age 6 and in 2005 for 11-year-olds, for clarity for those not familiar with the history of the vaccine schedule in Finland, it might be worth noting in the text also. Elomaa A, et al. Pertussis before and after the introduction of acellular pertussis vaccines in Finland. Vaccine 2009;40:5443-5449.

Answer: While you are correct that the vaccination schedule change involving the preschool booster at 4 years of age was implemented starting from 2005, the actual first recipients of the DTaP-IPV booster at 4 years of age were children <4months of age at 1.1.2005. During the transitional phase, children >4 months of age (who had received at least 2 doses of DTwP) at 1.1.2005 got the DTaP booster at the age of 6 as illustrated in the Figure 1. So, in practice the booster at ‘’4 years of age’’ was not started before 2008.

We have now further elaborated this in the introduction and added references, although the primary reference to the transitional phase vaccinations are only printed books in Finnish.

Lines 178-179 might help to clarify throughout the paper that booster versus no booster with varying number of primary doses is a relative VE analysis since absolute VE is also being assessed in this study.

Answer: We have added an elaboration regarding analyses of relative effectiveness for a few places in the manuscript. Specifically, in lines 176 and 540-541 and 552 (track changes version).

Lines 231-245 The before and after preschool aP booster analysis impacted by different time periods so baseline risk of pertussis infection could differ; different serologic assays likely impacting test performance, and different primary vaccine schedules and vaccines used.

Answer: You are correct and those are discussed in the Discussion chapters on Interpretation and on limitations. We added a few more sentences on this matter to the discussion.

Lines 423-424 could move comment about significantly “negative” VE result to the limitations section of the discussion. It may also be helpful to include some discussion about possible sources of “negative” VE that likely reflects the impact of bias rather than true negative biological effectiveness in settings of very low VE including differential depletion of susceptibles (Kahn R, et al. Identifying and Alleviating Bias Due to Differential Depletion of Susceptible People in Postmarketing Evaluations of COVID-19 Vaccines. Am J Epi 2022. <https://doi.org/10.1093/aje/kwac015>), increased healthcare seeking behavior associated with vaccination that result in greater testing and detection of both past and current infection among vaccinees, reducing estimated effectiveness (Griffith GJ, et al. Collider bias undermines our understanding of COVID-19 disease risk and severity. Nat Comm 2020), differential risk-taking behavior (Pelzman effect), etc.

Answer: Thank you for this suggestion. We have moved the comment of negative VE from the results to the limitations as suggested. We have now also described some of the above mentioned mechanisms that could have contributed to the observed negative VE estimate in the paragraphs describing potential biases in the analysis of VE by time since vaccination.

Line 428 Table 6 The authors could consider analysis of laboratory-confirmed pertussis temporally associated with hospitalization or ED admission to increase sample size and precision of the estimate.

Answer: Thank you for this excellent suggestion. Unfortunately, as this was not an objective in our study protocol and we followed the recommended principle of minimizing the extracted data. In the data extraction phase we have only requested HILMO (hospital discharge register)-records coded for inpatient hospitalization/hospital admission, which means that we don’t have ED records if the patient only visited ED and was not admitted to the hospital.

Only ED records (and all other HILMO and Avohilmo records) associated with a pertussis diagnosis were extracted (for the analysis of effectiveness against clinically diagnosed pertussis).

Thus, this suggested analysis cannot be conducted without a new data permit application to Findata and subsequent extraction of data. This would require substantial extra resources and time, which is why we consider this analysis to be unfeasible for this revision.

Line 452 It may be preferable for the Table 2 title to reflect association of 2aP preschool booster with reduced incidence of pertussis among children. There are many other factors that may have contributed to the observed differences, including differences in the primary vaccines and schedule, test methods and testing behaviors, and possible changes in pertussis disease epidemiology during the two study periods.

Answer: We assume you are referring to table 7 (current table 4). We have now changed the table title. Also, we changed a word in the abstract to from ‘’indicating’’ to ‘’suggesting’’ to better reflect the uncertainty around these estimates. These above mentioned limitations are discussed in the Discussion in chapters on interpretation and limitations.

Lines 459-466 Table 8 consider noting that this is an estimate of absolute VE. Nice negative control.

Answer: We added ‘’absolute effectiveness’’ to table 8 (current table 5).

Lines 498-500 While it appears that the specificity of serologic testing is likely the main reason VE is greater when the analysis is limited to cases identified by culture or PCR, this is an important point. What proportion of culture/PCR tests were obtained in the hospital (or ED and hospital) setting(s)?

Answer: Unfortunately, from the NIDR data, it is not possible to find out if the original sample was taken in a hospital/ED setting or primary care. We are neither able to indirectly assess the source of original sample, because as mentioned in the answer above, we have all data only on hospital admissions but not from other HILMO records such as individual ED visits.

Lines 490-494 consider reference. (e.g., Dalby T, et al. Antibody responses to pertussis toxin display different kinetics after clinical Bordetella pertussis infection than after vaccination with acellular pertussis vaccine. J Med Microbiol 2010; 59:1029-1036. doi: 10.1099/jmm.0.020826-0 and/or Guiso N, et al. What to do and what not to do in serological diagnosis of pertussis: recommendations from EU reference laboratories Eur J Clin Micro Infect Dis 2011)

Answer: Thank you for this suggestion, we added references to these articles as well as to the study by Aase et al on the antibody kinetics after the preschool booster in Norwegian children (the same 2aP preschool booster as with our study children).

Lines 563-567 consider adding reference as noted earlier.

Answer: We have now added references to the above mentioned studies/report, as recommended.

Lines 591-592 Given the very high rate of pertussis vaccine uptake in the study population, it is likely that many behaviors differ substantially between vaccinated and unvaccinated cohorts (e.g., health care seeking behaviors, diseases avoidance, etc) that could impact the analysis.

Answer: Yes that is true, we have now further elaborated that in the discussion about residual confounding.

Line 605 aP-containing primary vaccine series may not provide greater protection against pertussis disease than wP-containing primary vaccine series. (Klein NP, et al. Comparative effectiveness of acellular versus whole-cell pertussis vaccines in teenagers. Pediatrics 2013; 131:e1716-22. doi: 10.1542/peds.2012-3836.)

Answer: While short-term effectiveness of wP-containing primary series have been reported to be comparable to aP-containing series, substantial variability has been reported between different historical wP vaccines (most of them not currently in use). (Fulton et al <https://doi.org/10.1093/cid/ciw051>, Wilkinson et al <https://doi.org/10.1016/j.vaccine.2021.04.032> ).

In addition, the duration of protection might not be much different between wP and aP either (Vargas-Zambrano et al <https://doi.org/10.1007/s40121-025-01277-1> ). The study by Klein et al. suggests better protection among wP primed adolescents. However, interpretation may be complicated as the study compared older children primed with wP vaccines to younger children primed with aP vaccines.

As noted in the manuscript, the effectiveness of the historical Finnish wP vaccine is not precisely known. Therefore, it is possible that its effectiveness was lower than that of the current Finnish aP primary vaccination schedule, especially considering the high effectiveness of the aP primary series observed in our study.

This uncertainty is relevant to the interpretation of the preschool booster impact analysis, which assumes that factors other than implementation of the preschool booster did not substantially contribute to the observed changes in pertussis incidence. Consequently, differences between historical wP and current aP primary series effectiveness could particularly influence the indirect impact estimates in children younger than preschool booster age.

Sincerely,

Jussi Halme, M.D.
on behalf of all co-authors
