# Supplementary material for: Effectiveness and impact of the 2-component acellular pertussis vaccine as a preschool booster in Finland – A register-based study
Source: PLOS Glob Public Health. 2026 Jul 29;6(7):e0006800. doi: 10.1371/journal.pgph.0006800 (PMC13419238; doi:10.1371/journal.pgph.0006800)
Supplement: Peer review history

# Author comment: Author response to Decision Letter 0

**DOI:** 10.1371/journal.pgph.0006800.r001

**Transfer Alert**

This paper was transferred from another journal. As a result, its full editorial history (including decision letters, peer reviews and author responses) may not be present.

1 Apr 2026

Attachment Submitted filename: Response to Reviewers.docx

# Peer review: Decision Letter 0

**DOI:** 10.1371/journal.pgph.0006800.r002

**Authors:** Katia Bruxvoort

19 May 2026

PGPH-D-26-00869

Effectiveness and impact of the 2-component acellular pertussis vaccine as a preschool booster in Finland – A register-based study

PLOS Global Public Health

Dear Dr. Halme,

Thank you for submitting your manuscript to PLOS Global Public Health. After careful consideration, we feel that it has merit but does not fully meet PLOS Global Public Health’s publication criteria as it currently stands. Therefore, we invite you to submit a revised version of the manuscript that addresses the points raised during the review process.

Please submit your revised manuscript by Jun 18 2026 11:59PM. If you will need more time than this to complete your revisions, please reply to this message or contact the journal office at globalpubhealth@plos.org. When you're ready to submit your revision, log on to https://www.editorialmanager.com/pgph/ and select the 'Submissions Needing Revision' folder to locate your manuscript file.

Please include the following items when submitting your revised manuscript:

- A letter that responds to each point raised by the editor and reviewer(s). You should upload this letter as a separate file labeled 'Response to Reviewers'.
- A marked-up copy of your manuscript that highlights changes made to the original version. You should upload this as a separate file labeled 'Revised Manuscript with Track Changes'.
- An unmarked version of your revised paper without tracked changes. You should upload this as a separate file labeled 'Manuscript'.

Guidelines for resubmitting your figure files are available below the reviewer comments at the end of this letter.

As the corresponding author, your ORCID iD is verified in the submission system and will appear in the published article. PLOS supports the use of ORCID, and we encourage all coauthors to register for an ORCID iD and use it as well. Please encourage your coauthors to verify their ORCID iD within the submission system before final acceptance, as unverified ORCID iDs will not appear in the published article. *Only*  the individual author can complete the verification step; PLOS staff *cannot*  verify ORCID iDs on behalf of authors.

We look forward to receiving your revised manuscript.

Kind regards,

Katia Bruxvoort, PhD

Academic Editor

PLOS Global Public Health

**Journal Requirements:**

1. Please amend your detailed Financial Disclosure statement. This is published with the article. It must therefore be completed in full sentences and contain the exact wording you wish to be published.

i. Please clarify all sources of financial support for your study. List the grants, grant numbers, and organizations that funded your study, including funding received from your institution. Please note that suppliers of material support, including research materials, should be recognized in the Acknowledgements section rather than in the Financial Disclosure.

ii. State the initials, alongside each funding source, of each author to receive each grant. For example: "This work was supported by the National Institutes of Health (####### to AM; ###### to CJ) and the National Science Foundation (###### to AM)."

iii. State what role the funders took in the study. If the funders had no role in your study, please state: “The funders had no role in study design, data collection and analysis, decision to publish, or preparation of the manuscript.”

iv. If any authors received a salary from any of your funders, please state which authors and which funders.

2. In the online submission form, you indicated that “According to the Finnish law, the Act on the secondary use of health and social data, the data from national health registers used as a source of data in this study can only be published as anonymized aggregate results. Accessing individual-level data in scientific research is possible by requesting data from the Finnish Social and Health Data Permit Authority, Findata, responsible for granting permits for the secondary use of health data. Once a permit is granted and data extracted, the pseudonymized data can then be processed and analyzed by investigators in a secure operating environment. Requesting the permit and extraction of the data are subject to costs charged by Findata and register controllers. Detailed instructions for applying data permits and application forms are accessible from the Findata website: https://findata.fi/en/permits/. R code for conducting the analyses can be requested from the authors after obtaining relevant permission from Findata.”.

All PLOS journals now require all data underlying the findings described in their manuscript to be freely available to other researchers, either

1. In a public repository,

2. Within the manuscript itself, or

3. Uploaded as supplementary information.

This policy applies to all data except where public deposition would breach compliance with the protocol approved by your research ethics board. If your data cannot be made publicly available for ethical or legal reasons (e.g., public availability would compromise patient privacy), please explain your reasons by return email and your exemption request will be escalated to the editor for approval. Your exemption request will be handled independently and will not hold up the peer review process, but will need to be resolved should your manuscript be accepted for publication. One of the Editorial team will then be in touch if there are any issues.

3. We do not publish any copyright or trademark symbols that usually accompany proprietary names, eg (R), (C), or TM (e.g. next to drug or reagent names). Please remove all instances of trademark/copyright symbols throughout the text, including Tetravac® and Pentavac®.

4. Please upload a copy of Figure 5, which you refer to in your manuscript. Or, if the figure is no longer to be included as part of the submission please remove all reference to it within the text.

If the reviewer comments include a recommendation to cite specific previously published works, please review and evaluate these publications to determine whether they are relevant and should be cited. There is no requirement to cite these works unless the editor has indicated otherwise.

Please review your reference list to ensure that it is complete and correct. If you have cited papers that have been retracted, please include the rationale for doing so in the manuscript text, or remove these references and replace them with relevant current references. Any changes to the reference list should be mentioned in the rebuttal letter that accompanies your revised manuscript. If you need to cite a retracted article, indicate the article’s retracted status in the References list and also include a citation and full reference for the retraction notice.

**Additional Editor Comments (if provided):**

[Note: HTML markup is below. Please do not edit.]

Reviewers' comments:

Reviewer's Responses to Questions

**Comments to the Author**

1. Does this manuscript meet PLOS Global Public Health’s publication criteria? Is the manuscript technically sound, and do the data support the conclusions? The manuscript must describe methodologically and ethically rigorous research with conclusions that are appropriately drawn based on the data presented.

Reviewer #1: Yes

Reviewer #2: Yes

**********

2. Has the statistical analysis been performed appropriately and rigorously?

Reviewer #1: Yes

Reviewer #2: Yes

**********

3. Have the authors made all data underlying the findings in their manuscript fully available (please refer to the Data Availability Statement at the start of the manuscript PDF file)?

The PLOS Data policy requires authors to make all data underlying the findings described in their manuscript fully available without restriction, with rare exception. The data should be provided as part of the manuscript or its supporting information, or deposited to a public repository. For example, in addition to summary statistics, the data points behind means, medians and variance measures should be available. If there are restrictions on publicly sharing data—e.g. participant privacy or use of data from a third party—those must be specified.

Reviewer #1: Yes

Reviewer #2: Yes

**********

4. Is the manuscript presented in an intelligible fashion and written in standard English?

PLOS Global Public Health does not copyedit accepted manuscripts, so the language in submitted articles must be clear, correct, and unambiguous. Any typographical or grammatical errors should be corrected at revision, so please note any specific errors here.

Reviewer #1: Yes

Reviewer #2: Yes

**********

5. Review Comments to the Author

Please use the space provided to explain your answers to the questions above. You may also include additional comments for the author, including concerns about dual publication, research ethics, or publication ethics. (Please upload your review as an attachment if it exceeds 20,000 characters)

**Reviewer #1:**  Many thanks for the opportunity to review the manuscript "Effectiveness and impact of the 2-component acellular pertussis vaccine as a preschool booster in Finland – A register-based study" This comprehensive report of vaccine effectiveness of the acellular pertussis vaccine with a pre-school booster or not. The findings of a 2+1 schedule with a pre school booster is reassuring for the protectiveness of children against severe pertussis disease.

Some comments:

- I would suggest reducing the number of tables in the main paper and possibly include some as supplementary material

- Tables 1, 2 and 3 could be summarised into a flowchart of the exclusions and inclusions of children in the study that would included in the primary analysis as well as for the effectiveness analysis

- Table 5 - I do not understand why only those born after 2011 are summarised here? The 2 cohorts are children born 1990-1998 and 2007-2015. Also, is it necessary to show all aspects of age distribution - was age normal or not normally distributed in the cohorts?

- The discussion could be reduced in length and to concentrate on the main findings

- Line 380 - is meant to be figure 3 not figure 5?

**Reviewer #2:**  The authors of “Effectiveness and impact of the 2-component acellular pertussis vaccine as a preschool booster in Finland – A register-based study” evaluated the effectiveness and duration of protection associated with the preschool DTaP-IPV preschool booster and its population-level impact after implementation in the NIP in Finland. The manuscript is well-written and the findings are interesting. However, I have a few comments:

Lines 83-84: While apparent after reading the paper, it would help to clarify in the introduction that, along with the change to aP-containing vaccine given at 3, 5, and 12 months in 2005, the preschool booster at 4 years of age was added to the NIS in 2005 also. Furthermore, while it is clear from Figure 1 that children did not receive pertussis containing vaccine after 24 months of age until 2003 when the aP containing booster was introduced at age 6 and in 2005 for 11-year-olds, for clarity for those not familiar with the history of the vaccine schedule in Finland, it might be worth noting in the text also. Elomaa A, et al. Pertussis before and after the introduction of acellular pertussis vaccines in Finland. Vaccine 2009;40:5443-5449.

Lines 178-179 might help to clarify throughout the paper that booster versus no booster with varying number of primary doses is a relative VE analysis since absolute VE is also being assessed in this study.

Lines 231-245 The before and after preschool aP booster analysis impacted by different time periods so baseline risk of pertussis infection could differ; different serologic assays likely impacting test performance, and different primary vaccine schedules and vaccines used.

Lines 423-424 could move comment about significantly “negative” VE result to the limitations section of the discussion. It may also be helpful to include some discussion about possible sources of “negative” VE that likely reflects the impact of bias rather than true negative biological effectiveness in settings of very low VE including differential depletion of susceptibles (Kahn R, et al. Identifying and Alleviating Bias Due to Differential Depletion of Susceptible People in Postmarketing Evaluations of COVID-19 Vaccines. Am J Epi 2022. https://doi.org/10.1093/aje/kwac015), increased healthcare seeking behavior associated with vaccination that result in greater testing and detection of both past and current infection among vaccinees, reducing estimated effectiveness (Griffith GJ, et al. Collider bias undermines our understanding of COVID-19 disease risk and severity. Nat Comm 2020), differential risk-taking behavior (Pelzman effect), etc.

Line 428 Table 6 The authors could consider analysis of laboratory-confirmed pertussis temporally associated with hospitalization or ED admission to increase sample size and precision of the estimate.

Line 452 It may be preferable for the Table 2 title to reflect association of 2aP preschool booster with reduced incidence of pertussis among children. There are many other factors that may have contributed to the observed differences, including differences in the primary vaccines and schedule, test methods and testing behaviors, and possible changes in pertussis disease epidemiology during the two study periods.

Lines 459-466 Table 8 consider noting that this is an estimate of absolute VE. Nice negative control.

Lines 498-500 While it appears that the specificity of serologic testing is likely the main reason VE is greater when the analysis is limited to cases identified by culture or PCR, this is an important point. What proportion of culture/PCR tests were obtained in the hospital (or ED and hospital) setting(s)?

Lines 490-494 consider reference. (e.g., Dalby T, et al. Antibody responses to pertussis toxin display different kinetics after clinical Bordetella pertussis infection than after vaccination with acellular pertussis vaccine. J Med Microbiol 2010; 59:1029-1036. doi: 10.1099/jmm.0.020826-0 and/or Guiso N, et al. What to do and what not to do in serological diagnosis of pertussis: recommendations from EU reference laboratories Eur J Clin Micro Infect Dis 2011)

Lines 563-567 consider adding reference as noted earlier.

Lines 591-592 Given the very high rate of pertussis vaccine uptake in the study population, it is likely that many behaviors differ substantially between vaccinated and unvaccinated cohorts (e.g., health care seeking behaviors, diseases avoidance, etc) that could impact the analysis.

Line 605 aP-containing primary vaccine series may not provide greater protection against pertussis disease than wP-containing primary vaccine series. (Klein NP, et al. Comparative effectiveness of acellular versus whole-cell pertussis vaccines in teenagers. Pediatrics 2013; 131:e1716-22. doi: 10.1542/peds.2012-3836.)

**********

6. PLOS authors have the option to publish the peer review history of their article (what does this mean?). If published, this will include your full peer review and any attached files.

**Do you want your identity to be public for this peer review?**  If you choose “no”, your identity will remain anonymous but your review may still be made public.

For information about this choice, including consent withdrawal, please see our Privacy Policy.

Reviewer #1: No

Reviewer #2: No

**********

[NOTE: If reviewer comments were submitted as an attachment file, they will be attached to this email and accessible via the submission site. Please log into your account, locate the manuscript record, and check for the action link "View Attachments". If this link does not appear, there are no attachment files.]

**Figure Resubmissions:**

While revising your submission, we strongly recommend that you use PLOS’s NAAS tool (https://ngplosjournals.pagemajik.ai/artanalysis) to test your figure files. NAAS can convert your figure files to the TIFF file type and meet basic requirements (such as print size, resolution), or provide you with a report on issues that do not meet our requirements and that NAAS cannot fix.

After uploading your figures to PLOS’s NAAS tool - https://ngplosjournals.pagemajik.ai/artanalysis, NAAS will process the files provided and display the results in the "Uploaded Files" section of the page as the processing is complete. If the uploaded figures meet our requirements (or NAAS is able to fix the files to meet our requirements), the figure will be marked as "fixed" above. If NAAS is unable to fix the files, a red "failed" label will appear above. When NAAS has confirmed that the figure files meet our requirements, please download the file via the download option, and include these NAAS processed figure files when submitting your revised manuscript.

# Author comment: Author response to Decision Letter 1

**DOI:** 10.1371/journal.pgph.0006800.r003

18 Jun 2026

Attachment Submitted filename: Response to reviewers-revision 18.6.2026.docx

# Editor report: Decision Letter 1

**DOI:** 10.1371/journal.pgph.0006800.r004

**Authors:** Katia Bruxvoort

7 Jul 2026

Effectiveness and impact of the 2-component acellular pertussis vaccine as a preschool booster in Finland – A register-based study

PGPH-D-26-00869R1

Dear Mr Halme,

We are pleased to inform you that your manuscript 'Effectiveness and impact of the 2-component acellular pertussis vaccine as a preschool booster in Finland – A register-based study' has been provisionally accepted for publication in PLOS Global Public Health.

Before your manuscript can be formally accepted you will need to complete some formatting changes, which you will receive in a follow up email. A member of our team will be in touch with a set of requests.

Please note that your manuscript will not be scheduled for publication until you have made the required changes, so a swift response is appreciated.

IMPORTANT: The editorial review process is now complete. PLOS will only permit corrections to spelling, formatting or significant scientific errors from this point onwards. Requests for major changes, or any which affect the scientific understanding of your work, will cause delays to the publication date of your manuscript.

If your institution or institutions have a press office, please notify them about your upcoming paper to help maximize its impact. If they'll be preparing press materials, please inform our press team as soon as possible -- no later than 48 hours after receiving the formal acceptance. Your manuscript will remain under strict press embargo until 2 pm Eastern Time on the date of publication. For more information, please contact globalpubhealth@plos.org.

Thank you again for supporting Open Access publishing; we are looking forward to publishing your work in PLOS Global Public Health.

Best regards,

Katia Bruxvoort, PhD

Academic Editor

PLOS Global Public Health

***********************************************************

Reviewer Comments (if any, and for reference):
